# An additive-noise approximation to Keller–Segel–Dean–Kawasaki dynamics: local well-posedness of paracontrolled solutions

**DOI:** 10.1007/s40072-024-00343-y

**Published:** 2025-01-24

**Authors:** Adrian Martini, Avi Mayorcas

**Affiliations:** 1 Department of Statistics, University of Oxford, Oxford, UK; 2 Department of Mathematical Sciences, University of Bath, Bath, UK

**Keywords:** Singular stochastic partial differential equation, Paracontrolled distributions, Linear fluctuating hydrodynamics, Parabolic-elliptic Keller–Segel model, Dean–Kawasaki equation, Primary 60H17, Secondary 60L40, 92C17

## Abstract

Using the method of paracontrolled distributions, we show the local well-posedness of an additive-noise approximation to the fluctuating hydrodynamics of the Keller–Segel model on the two-dimensional torus. Our approximation is a non-linear, non-local, parabolic-elliptic stochastic PDE with an irregular, heterogeneous space-time noise. As a consequence of the irregularity and heterogeneity, solutions to this equation must be renormalised by a sequence of diverging fields. Using the symmetry of the elliptic Green’s function, which appears in our non-local term, we establish that the renormalisation diverges at most logarithmically, an improvement over the linear divergence one would expect by power counting. Similar cancellations also serve to reduce the number of diverging counterterms.

## Introduction

In this work we are concerned with the local well-posedness of singular stochastic partial differential equations (singular SPDEs) of the kind

1.1 $$\begin{aligned} {\left\{ \begin{array}{ll} \begin{aligned} (\partial _t-\Delta )\rho & =\nabla \cdot (\rho \nabla \Phi _{\rho })+\nabla \cdot (\sigma \varvec{\xi }), & &  \text { in } (0,T]\times \mathbb {T}^2,\\ -\Delta \Phi _{\rho }& =\rho - \langle \rho ,1\rangle _{L^2(\mathbb {T}^{2})},& &  \text { in }(0,T]\times \mathbb {T}^2,\\ \rho |_{t=0}& =\rho _{0}, & &  \text { on }\mathbb {T}^2, \end{aligned} \end{array}\right. } \end{aligned}$$

where $\varvec{\xi }=(\xi ^1,\xi ^2)$ is a two-dimensional vector of i.i.d. space-time white noises, $\mathbb {T}^2=\mathbb {R}^2/\mathbb {Z}^2$ is the two-dimensional torus, $T\in (0,\infty )$ is a time horizon, $\sigma \in C([0,T];\mathcal {H}^2(\mathbb {T}^2))$ is a space-time heterogeneity and $\rho _0$ is some suitable initial data which we specify later. The advection in (1.1) comes from the Keller–Segel model of chemotaxis [37] and the noise stems from the theory of fluctuating hydrodynamics where one would ideally set $\sigma = \sqrt{\rho }$ to obtain the Dean–Kawasaki noise [16, 33]. However, it was shown in [34, 35] that the equation with smooth drift only admits solutions which are empirical measures of the underlying interacting particle system. Hence one does not expect (1.1) with $\sigma = \sqrt{\rho }$ to admit non-atomic solutions.

Motivated by the theory of linear fluctuating hydrodynamics, our main example is instead given by the choice $\sigma = \sqrt{\rho _{\text {det}}}$ where $\rho _{\text {det}}$ solves the deterministic PDE

1.2 $$\begin{aligned} {\left\{ \begin{array}{ll} \begin{aligned} (\partial _t-\Delta )\rho _{\text {det}}& =\nabla \cdot (\rho _{\text {det}}\nabla \Phi _{\rho _{\text {det}}}), & &  \text { in } (0,T]\times \mathbb {T}^2,\\ -\Delta \Phi _{\rho _{\text {det}}}& =\rho _{\text {det}}-\langle \rho _{\text {det}},1\rangle _{L^2(\mathbb {T}^{2})}, & &  \text { in }(0,T]\times \mathbb {T}^2,\\ \rho _{\text {det}}|_{t=0}& =\rho _{0}, & &  \text { on }\mathbb {T}^2. \end{aligned} \end{array}\right. } \end{aligned}$$

This choice will be applied in a follow-up paper, [41], to an additive-noise approximation of the Dean–Kawasaki equation associated to the Keller–Segel model. More precisely, given a *noise intensity* $\varepsilon >0$ and a *correlation length* $\delta =\delta (\varepsilon )>0$, we define the additive-noise approximation $\rho ^{(\varepsilon )}_{\delta }$ as a solution to

1.3 $$\begin{aligned} {\left\{ \begin{array}{ll} \begin{aligned} (\partial _t-\Delta )\rho & =\nabla \cdot (\rho \nabla \Phi _{\rho })+\varepsilon ^{1/2}\nabla \cdot (\sqrt{\rho _{\text {det}}}\varvec{\xi }^{\delta }), & &  \text { in } (0,T]\times \mathbb {T}^2,\\ -\Delta \Phi _{\rho }& =\rho - \langle \rho ,1\rangle _{L^2(\mathbb {T}^{2})},& &  \text { in }(0,T]\times \mathbb {T}^2,\\ \rho |_{t=0}& =\rho _{0}, & &  \text { on }\mathbb {T}^2, \end{aligned} \end{array}\right. } \end{aligned}$$

where $\varvec{\xi }^\delta {:}{=}(\psi _{\delta }*\xi ^1,\psi _{\delta }*\xi ^2)$ denotes a mollified noise and $\psi _{\delta }$ a standard, symmetric mollifier. Under appropriate scaling assumptions on $(\varepsilon ,\delta (\varepsilon ))$, using the theory developed in this paper, we will show in [41] that $(\rho ^{(\varepsilon )}_{\delta (\varepsilon )})_{\varepsilon >0}$ satisfies a law of large numbers with limit $\rho _{\text {det}}$, a central limit theorem with leading-order fluctuations given by the generalized Ornstein–Uhlenbeck process and a large deviation principle. This relates our additive-noise approximation (1.3) to stochastic particle approximations to the Keller–Segel model [18, 19], which were shown to have the same law of large numbers [4–6, 18, 50]—and are conjectured to have the same central limit theorem^1^ —as our approximation (1.3).

### Remark 1.1

We may also consider equations of the form

1.4 $$\begin{aligned} {\left\{ \begin{array}{ll} \begin{aligned} (\partial _t-\Delta )\rho _{\text {det}}& =-\chi \nabla \cdot (\rho _{\text {det}}\nabla \Phi _{\rho _{\text {det}}}), & &  \text { in } (0,T]\times \mathbb {T}^2,\\ -\Delta \Phi _{\rho _{\text {det}}}& =\rho _{\text {det}}-\langle \rho _{\text {det}},1\rangle _{L^2(\mathbb {T}^{2})}, & &  \text { in }(0,T]\times \mathbb {T}^2,\\ \rho _{\text {det}}|_{t=0}& =\rho _{0}, & &  \text { on }\mathbb {T}^2, \end{aligned} \end{array}\right. } \end{aligned}$$

where $\chi \in \mathbb {R}$. In this setting, when one restricts $\rho _0$ to be non-negative and to integrate to 1 (i.e. the density of a probability measure) one recovers the usual parabolic-elliptic Keller–Segel equation, [37], the analysis of which has received much attention [29–31, 47]. The global existence of (1.4) in spatial dimension two depends on the size and sign of $\chi$, [3, 11, 32]. Since we are only concerned with local existence and all of our analysis is agnostic as to the size and sign of $\chi$ we set it to be $-1$ and work with equations of the form (1.1) and (1.2).

In this paper, rather than the particular (1.3), we will treat the general (1.1) where $\sigma$ is an arbitrary function, continuous in time and $\mathcal {H}^2$ in space. Due to the singularity of the noise, defining a suitable notion of solution to (1.1) is non-trivial and we will implement a paracontrolled approach to obtain local well-posedness, [22]. To see why such an approach is necessary we consider the terms of (1.1) under a formal power counting argument. The proper definition of all function spaces used below can be found in Appendix 1.

For any $T>0$, the white noise almost surely takes values in $\mathcal {C}_{\text {par}}^{-2-}([0,T]\times \mathbb {T}^2)$.^2^ Let us assume for now that we can define the product ${\sigma \varvec{\xi }}$ intrinsically and that it is no more regular than the white noise itself. Due to the regularising effect of the heat equation, it follows that the solution  to the linear equation,may be no more regular than $C_T\mathcal {C}^{-1-}$. Here $\mathcal {C}^{\alpha }=\mathcal {C}^{\alpha }(\mathbb {T}^{2})$ denotes the Hölder–Besov space of regularity $\alpha \in \mathbb {R}$ and $C_{T}\mathcal {C}^{\alpha }$ denotes the space of continuous functions on [0, *T*] taking values in $\mathcal {C}^{\alpha }$. Assuming that this regularity is passed to $\rho$ and applying the regularising effect of the elliptic equation we would have $\nabla \Phi _{\rho } \in C_T\mathcal {C}^{0-}$. However, by Bony’s estimate the product *fg* is only a priori well-defined for $f \in \mathcal {C}^{\alpha }$ and $g\in \mathcal {C}^\beta$ with $\alpha +\beta >0$, which does not hold for $\rho \nabla \Phi _{\rho }$.

The theories of regularity structures, paracontrolled distributions, renormalisation groups and various recent extensions and adaptations thereof have revolutionised the study of singular SPDEs [17, 22, 26, 38, 39, 46]. The common thread throughout these theories is to notice that the factors of the ill-defined products are not generic distributions but inherent structure from the noise. This inheritance allows one to define renormalised products, which excise the singular part, allowing one to give meaning to a renormalised equation which is continuous in a finite tuple of *diagrams* built from the noise. The noise, along with these diagrams, is referred to as an enhancement.

The theory of paracontrolled distributions was first developed by M. Gubinelli, P. Imkeller and N. Perkowski in [22]. The central idea is to use harmonic analysis to construct regular commutators, which allow us to decompose the equation into exogenous noise terms and terms that can be constructed as fixed points. Paracontrolled distributions have been successfully applied to analyse a range of singular SPDEs and operators including; the parabolic Anderson model (PAM) [22, 36], the Anderson Hamiltonian [1, 12, 25], the $\Phi ^{4}_{3}$ model [9, 43], the Kardar–Parisi–Zhang equation [24], the stochastic Burgers and Navier–Stokes equations [24, 55] and the stochastic non-linear wave equation [23].

In our case, we find that there exists a deterministic field $f^\delta :[0,T]\times \mathbb {T}^2\rightarrow \mathbb {R}^2$ satisfying the bound,

$$\begin{aligned} \Vert f^{\delta }(t)\Vert _{\mathcal {C}^{-1-}}\lesssim (1\vee \log (\delta ^{-1}))\Vert \sigma \Vert ^{2}_{C_{T}\mathcal {H}^{2}}, \end{aligned}$$

and such that the sequence of solutions $(\rho ^\delta )_{\delta >0}$ each solving,

1.5 $$\begin{aligned} {\left\{ \begin{array}{ll} \begin{aligned} (\partial _t-\Delta )\rho & =\nabla \cdot (\rho \nabla \Phi _{\rho }-f^\delta )+\nabla \cdot (\sigma \varvec{\xi }^{\delta }), & &  \text { in } (0,T]\times \mathbb {T}^2,\\ -\Delta \Phi _{\rho }& =\rho -\langle \rho ,1\rangle _{L^2(\mathbb {T}^{2})}, & &  \text { in }(0,T]\times \mathbb {T}^2,\\ \rho |_{t=0}& =\rho _{0}, & &  \text { on }\mathbb {T}^2, \end{aligned} \end{array}\right. } \end{aligned}$$

converges in probability to a unique limit $\rho$, which we identify as the renormalised solution to (1.1). For a definition of the renormalised solution, see Definition 3.21; for its existence, see Theorem 3.22, Part 1; and for the convergence, see Theorem 3.22, Part 2.

Three points of interest arise from (1.5). Firstly, in the case where $\sigma$ is genuinely heterogeneous the field $f^\delta$ is in general also heterogeneous. This has been observed elsewhere, having been pointed out as a possibility in [26] and seen explicitly in the renormalisation of singular SPDEs on bounded domains, [21]. Secondly, if $\sigma$ is a constant, so that our noise agrees with that of the stochastic Burgers’ equation, then $f^{\delta }$ is zero. In this case the renormalised equation agrees exactly with the singular equation, i.e. the products are not explicitly renormalised when $\delta =0$. This phenomenon has also been observed in [13, 15, 24, 55]. Thirdly, using the informal power counting described above, one might expect the singular product $\rho ^\delta \nabla \Phi _{\rho ^\delta }$ to diverge at the order of $\delta ^{-1}$, since this is the gap in regularity between the singular factors. However, (1.5) shows that this divergence is at most logarithmic. This improvement arises from symmetries in the fundamental solution of the elliptic problem, leading to non-trivial cancellations in our stochastic estimates. In the case of constant $\sigma$ it is exactly these cancellations which show that no explicit renormalisation in (1.5) is necessary.

To demonstrate the underlying principle, let us consider a one-dimensional example. We assume that $u^{\delta }\rightarrow u$ as $\delta \rightarrow 0$ in a space of regularity $-1/2-$. The product rule gives the identity,

1.6 $$\begin{aligned} u^{\delta }\partial _{x}\partial _{x}^{-2}u^{\delta }=\frac{1}{2}\Bigl (\partial _{x}^{2}(\partial _{x}^{-1}u^{\delta }\partial _{x}^{-2}u^{\delta })-\partial _{x}(u^{\delta }\partial _{x}^{-2}u^{\delta })\Bigr ), \end{aligned}$$

where we write $\partial _x^{-1}$ as a shorthand for integration in *x*, with the (arbitrary) normalisation that the primitive is mean-free. While the product on the left hand side, between an object converging in $-1/2-$ and an object converging in $1/2-$ looks ill-posed, the right hand side is in fact classically well-posed; the first term is the second derivative of a product between objects in $1/2-$ and $3/2-$, while the second is the derivative of a product between an object in $-1/2-$ and one in $3/2-$. Hence the anticipated logarithmic divergence of the left hand side is removed by expanding as on the right hand side. This basic observation extends to our higher-dimensional case through the symmetry of the Green’s function for Poisson’s equation. We see that the symmetry alleviates divergences by one order. Linear divergences of $\delta ^{-1}$ are improved to logarithmic, and logarithmic divergences are improved to well-posedness. The heterogeneity $\sigma$ makes these improvements visible, as when $\sigma$ is constant the same symmetries lead to perfect cancellations removing the need for renormalising counterterms all together. Similar observations have also been made in the context of the KPZ equation, [24, Lem. 9.5].

We observe that (1.1) is an example of a singular SPDE involving anisotropic regularity since the regularising effect of the elliptic equation only takes place in the spatial variable. It is for this reason that we choose to work with the theory of paracontrolled distributions, since it naturally treats space and time separately. This is in contrast to the theory of regularity structures which naturally treats space and time simultaneously. While there are examples of works which study anisotropic equations and associated regularity structures, [7, 8, 28], the analysis is somewhat ad hoc and requires a significant amount of bespoke machinery to be developed. It is for this reason that we opt for a paracontrolled approach which more natively applies to the anisotropic regularisation present in (1.1).

**Structure of the Paper:** In the rest of this section we first recall some basic notations and conventions which are used throughout the text. Some of these are accompanied by more rigorous presentations in the appendices. We then present an outline of the general strategy and our main result in Sect. 1.2. Section 2 contains a detailed proof of the existence and regularity of the various stochastic objects which we are required to construct and constitute our enhanced noise. The careful analysis of these stochastic objects and control over the diverging fields is the main contribution of this paper. In Sect. 3 we show the local well-posedness of paracontrolled solutions given a suitable enhancement of the noise. Finally we include three appendices: Appendix 1 recalls some useful and well-known results concerning Besov spaces and paraproducts; Appendix 1 provides various estimates on the so-called shape coefficients which we introduce in Sect. 2 and Appendix 1, contains a number of summation and discrete convolution estimates that we make repeated use of throughout the text.

### Notations and conventions

We write $\mathbb {N}$ for the natural numbers excluding zero, $\mathbb {N}_0 {:}{=}\mathbb {N}\cup \{0\}$ and $\mathbb {N}_{-1} {:}{=}\mathbb {N}_0\cup \{-1\}$. We define the two-dimensional torus by $\mathbb {T}^{2}{:}{=}\mathbb {R}^{2}/\mathbb {Z}^{2}$. Throughout $|\,\cdot \,|$ will indicate the norm $|x|=\big (\sum _{i=1}^2 |x_i|^2\big )^{1/2}$. Occasionally we write $|x|_{\infty }{:}{=}\max _{i =1,2} |x_i |$ to indicate the maximum norm on $\mathbb {T}^2$ or $\mathbb {R}^2$. For $r>0$ we use the notation $B(0,r){:}{=}\{x\in \mathbb {R}^2:|x|<r\}$. From now on we will write $\langle a,b\rangle$ to denote the inner product on any Hilbert space which we either specify or leave clear from the context. For $k,n\in \mathbb {N}$ we denote by $C^k(\mathbb {T}^2;\mathbb {R}^n)$ (resp. $C^\infty (\mathbb {T}^2;\mathbb {R}^n)$) the space of *k*-times continuously differentiable (resp. smooth), 1-periodic functions taking values in $\mathbb {R}^n$. We write $\mathcal {S}'(\mathbb {T}^2;\mathbb {R}^n)$ for the dual of $C^\infty (\mathbb {T}^2;\mathbb {R}^n)$. When the context is clear we will remove the target space so as to lighten notation.

For $f\in C^\infty (\mathbb {T}^2;\mathbb {R})$ (resp. complex sequences $(\zeta (\omega ))_{\omega \in \mathbb {Z}^{2}}$ with $\overline{\zeta (-\omega )}=\zeta (\omega )$ that decay faster than any polynomial) we define its Fourier transform (resp. inverse Fourier transform) by the expression,

$$\begin{aligned} \mathscr {F}f(\omega ) {:}{=}\int _{\mathbb {T}^2}\textrm{e}\hspace{0.3888pt}^{-2\pi \textrm{i}\hspace{0.3888pt}\langle \omega ,x\rangle }f(x)\textrm{d}x,\qquad \mathscr {F}^{-1} \zeta (x) {:}{=}\sum _{\omega \in \mathbb {Z}^2}\textrm{e}\hspace{0.3888pt}^{2\pi \textrm{i}\hspace{0.3888pt}\langle \omega ,x\rangle }\zeta (\omega ). \end{aligned}$$

This is extended componentwise to vector-valued functions, by density to $f\in L^p(\mathbb {T}^2;\mathbb {R}^n)$ for $p\in [1,\infty )$ and by duality to $f\in \mathcal {S}'(\mathbb {T}^2;\mathbb {R}^n)$. Where convenient we use the shorthand $\widehat{f}(\omega ){:}{=}\mathscr {F}f (\omega )$. We define the Sobolev space $\mathcal {H}^{\alpha }(\mathbb {T}^{2};\mathbb {R}^{n})$ of regularity $\alpha \in \mathbb {R}$ as the space of periodic distributions $u\in \mathcal {S}'(\mathbb {T}^{2};\mathbb {R}^{n})$ such that

$$\begin{aligned} \Vert u\Vert _{\mathcal {H}^{\alpha }}{:}{=}\Big (\sum _{\omega \in \mathbb {Z}^{2}}(1+|2\pi \omega |^{2})^{\alpha }|\widehat{u}(\omega )|^{2}\Big )^{1/2}<\infty . \end{aligned}$$

These notions also extend to complex-valued distributions, which we denote by $\mathcal {S}'(\mathbb {T}^{2};\mathbb {C}^{n})$ and $\mathcal {H}^{\alpha }(\mathbb {T}^{2};\mathbb {C}^{n})$.

We often work in the scale of Besov and Hölder–Besov spaces whose definitions and basic properties are recalled in Appendix 1. We let $\varrho _{-1},\varrho _{0}\in C^{\infty }(\mathbb {R}^{2};[0,1])$ be radially symmetric and such that $\text {supp}(\varrho _{-1})\subset B(0,1/2)$, $\text {supp}(\varrho _{0})\subset \{x\in \mathbb {R}^{2}:9/32\le |x|\le 1\}$ and assume for any $x \in \mathbb {R}^2$, that $\sum _{k=-1}^{\infty }\varrho _k(x)=1$, where $\varrho _{k}(x){:}{=}\varrho _{0}(2^{-k}x)$ for each $k\in \mathbb {N}$. This defines a dyadic partition of unity as in Appendix 1. Given $k\ge -1$ we write $\Delta _{k}u{:}{=}\mathscr {F}^{-1}(\varrho _{k}\mathscr {F}u)$ for the associated Littlewood–Paley block and given $\alpha \in \mathbb {R}$, $p,q\in [1,\infty ]$, we define the Besov-norm $\Vert u\Vert _{\mathcal {B}^{\alpha }_{p,q}(\mathbb {T}^2;\mathbb {R}^{n})}{:}{=}\Vert (2^{k\alpha }\Vert \Delta _{k}u\Vert _{L^{p}(\mathbb {T}^2;\mathbb {R}^{n})})_{k\in \mathbb {N}_{-1}}\Vert _{\ell ^q}$ for all $u\in \mathcal {S}'(\mathbb {T}^{2};\mathbb {R}^{n})$. We use $\mathcal {B}^\alpha _{p,q}(\mathbb {T}^2;\mathbb {R}^n)$ to denote the completion of $C^{\infty }(\mathbb {T}^{2};\mathbb {R}^{n})$ under $\Vert \,\cdot \,\Vert _{\mathcal {B}^\alpha _{p,q}(\mathbb {T}^2;\mathbb {R}^n)}$ and use the shorthand $\mathcal {C}^\alpha (\mathbb {T}^2;\mathbb {R}^n){:}{=}\mathcal {B}^\alpha _{\infty ,\infty }(\mathbb {T}^2;\mathbb {R}^n)$. As above, we will often remove the domain and target spaces when the context is clear. For $\alpha \in \mathbb {R}$ and $p,q\in [1,\infty ]$ we use the notation $\mathcal {B}^{\alpha -}_{p,q}{:}{=}\cap _{\alpha '<\alpha } \mathcal {B}^{\alpha '}_{p,q}$.

We define the action of the heat semigroup on $f \in L^1(\mathbb {T}^2;\mathbb {R})$ by the flow,

$$\begin{aligned} {[}0,\infty ) \ni t\mapsto P_t f {:}{=}\mathscr {F}^{-1}(\textrm{e}\hspace{0.3888pt}^{-t|2\pi \,\cdot \,|^{2}} \widehat{f}(\,\cdot \,)) = \mathscr {H}_t*f \end{aligned}$$

where the heat kernel $\mathscr {H}_{t}$ on $\mathbb {T}^2$ is defined by the expressions,

$$\begin{aligned} \mathscr {H}_t(x) {:}{=}\frac{1}{4\pi t}\sum _{n\in \mathbb {Z}^2} \textrm{e}\hspace{0.3888pt}^{-\frac{|x-n|^2}{4t}} \mathbb {1}_{[0,\infty )}(t)= \sum _{\omega \in \mathbb {Z}^2} \textrm{e}\hspace{0.3888pt}^{2 \pi \textrm{i}\hspace{0.3888pt}\langle \omega ,x\rangle } \textrm{e}\hspace{0.3888pt}^{-t|2\pi \omega |^2} \mathbb {1}_{[0,\infty )}(t)\qquad \text {for}~t\ne 0 \end{aligned}$$

and

$$\begin{aligned} \mathscr {H}_{0}{:}{=}\sum _{n\in \mathbb {Z}^{2}}\delta _{n}. \end{aligned}$$

For $f:[0,T]\times \mathbb {T}^{2}\rightarrow \mathbb {R}$, we define the resolution of the heat equation as

$$\begin{aligned} \mathcal {I}[f]_{t}{:}{=}\int _{0}^{t}P_{t-s}f_s\textrm{d}s=\int _{0}^{t}\mathscr {H}_{t-s}*f_s\textrm{d}s. \end{aligned}$$

We define the solution to the elliptic equation by

$$\begin{aligned} \Phi _{f}{:}{=}\mathscr {G}*(f-\langle f,1\rangle _{L^{2}(\mathbb {T}^{2})}),\qquad f\in \mathcal {S}'(\mathbb {T}^{2};\mathbb {R}), \end{aligned}$$

where for mean-free functions (resp. distributions),

$$\begin{aligned} (\mathscr {G}*f)(x){:}{=}(-\Delta )^{-1}f(x) {:}{=}\sum _{\omega \in \mathbb {Z}^2\setminus \{0\}} \textrm{e}\hspace{0.3888pt}^{2 \pi \textrm{i}\hspace{0.3888pt}\langle \omega ,x\rangle }\frac{1}{|2\pi \omega |^{2}} \widehat{f}(\omega ). \end{aligned}$$

Given a Banach space *E*, a subset $I\subseteq [0,\infty )$ and $\kappa \in (0,1]$, we write $C_IE{:}{=}C(I;E)$ (resp. $C^{\kappa }_I E {:}{=}C^{\kappa }(I;E)$) for the space of bounded, continuous (resp. $\kappa$-Hölder continuous) maps $f:I\rightarrow E$ equipped with the norm $\Vert f\Vert _{C_IE}{:}{=}\sup _{t\in I} \Vert f_t\Vert _E$ (resp. $\Vert f\Vert _{C_I^{\kappa }E}{:}{=}\Vert f\Vert _{C_IE}+ \sup _{s\ne t \in I}\frac{\Vert f_t-f_s\Vert _{E}}{|t-s|^{\kappa }}$). For $T>0$, we use the shorthands $C_TE=C_{[0,T]}E$ and $C_T^\kappa E=C_{[0,T]}^\kappa E$. Note that the norm $\Vert f\Vert _{C_T^{\kappa }E}$ is equivalent to $\Vert f_0\Vert _E + \sup _{s\ne t \in [0,T]}\frac{\Vert f_t-f_s\Vert _E}{|t-s|^\kappa }$. For $\eta \ge 0$ we define the Banach space

$$\begin{aligned} \begin{aligned} C_{\eta ;T}E{:}{=}\Big \{f:(0,T]\rightarrow E:~&(0,T]\ni t\mapsto (1\wedge t)^{\eta }f_{t}~\text {is continuous in}~E,\\&\qquad \quad [6](1\wedge t)^{\eta }f_{t}\big |_{t=0}{:}{=}\lim _{t\rightarrow 0}(1\wedge t)^{\eta }f_{t}\in E\Big \} \end{aligned} \end{aligned}$$

equipped with the norm

$$\begin{aligned} \Vert f\Vert _{C_{\eta ;T}E}{:}{=}\sup _{t\in (0,T]}(1\wedge t)^{\eta }\Vert f_{t}\Vert _{E}. \end{aligned}$$

We refer to $\eta$ as the weight at 0. For $\kappa \in (0,1]$ we let $C^\kappa _{\eta ;T}E{:}{=}C^\kappa _\eta ((0,T];E)$ denote the Banach space of functions $f:(0,T]\rightarrow E$ which are finite under the norm,

$$\begin{aligned} \Vert f\Vert _{C^{\kappa }_{\eta ;T}E}{:}{=}\Vert f\Vert _{C_{\eta ;T}E}+\sup _{s\ne t\in (0,T]}(1\wedge s\wedge t)^{\eta }\frac{\Vert f_{t}-f_{s}\Vert _{E}}{\Vert t-s\Vert ^{\kappa }}. \end{aligned}$$

We also make use of the notation $\mathscr {L}_{\eta ;T}^{\kappa }\mathcal {C}^{\alpha }{:}{=}C_{\eta ;T}^{\kappa }\mathcal {C}^{\alpha -2\kappa }\cap C_{\eta ;T}\mathcal {C}^{\alpha }$ to denote a weighted interpolation space, on which we define

$$\begin{aligned} \Vert u\Vert _{\mathscr {L}_{\eta ;T}^{\kappa }\mathcal {C}^{\alpha }}{:}{=}\max \{\Vert u\Vert _{C_{\eta ;T}^{\kappa }\mathcal {C}^{\alpha -2\kappa }},\Vert u\Vert _{C_{\eta ;T}\mathcal {C}^{\alpha }}\}. \end{aligned}$$

We set $\mathscr {L}_{T}^{\kappa }\mathcal {C}^{\alpha }{:}{=}\mathscr {L}_{0;T}^{\kappa }\mathcal {C}^{\alpha }{:}{=}C_{T}^{\kappa }\mathcal {C}^{\alpha -2\kappa }\cap C_{T}\mathcal {C}^{\alpha }$ and understand $\mathscr {L}^0_{\eta ;T}\mathcal {C}^{\alpha } = C_{\eta ;T}\mathcal {C}^{\alpha }$.

We write $\lesssim$ to indicate that an inequality holds up to a constant depending on quantities that we do not keep track of or are fixed throughout. When we do wish to emphasise the dependence on certain quantities $\alpha$, *p*, *d*, we either write $\lesssim _{\alpha ,p,d}$ or define $C{:}{=}C(\alpha ,p,d)>0$ and write $\le C$.

Let $u,v\in \mathcal {S}'(\mathbb {T}^{2})$, we define the truncated sums

1.7 $$\begin{aligned} \sum _{\begin{array}{c} \omega _1,\omega _2\in \mathbb {Z}^{2}\\ \omega _1\sim \omega _2 \end{array}}\widehat{u}(\omega _1)\widehat{v}(\omega _2){:}{=}\sum _{\omega _1,\omega _2\in \mathbb {Z}^{2}}\widehat{u}(\omega _1)\widehat{v}(\omega _2)\sum _{\begin{array}{c} k,l\in \mathbb {N}_{-1}\\ |k-l|\le 1 \end{array}}\varrho _k(\omega _1)\varrho _{l}(\omega _2) \end{aligned}$$

and

1.8 $$\begin{aligned} \sum _{\begin{array}{c} \omega _1,\omega _2\in \mathbb {Z}^{2}\\ \omega _1\precsim \omega _2 \end{array}}\widehat{u}(\omega _1)\widehat{v}(\omega _2){:}{=}\sum _{\omega _1,\omega _2\in \mathbb {Z}^{2}}\widehat{u}(\omega _1)\widehat{v}(\omega _2)\sum _{k=1}^{\infty }\sum _{l=-1}^{k-2}\varrho _{l}(\omega _1)\varrho _{k}(\omega _2), \end{aligned}$$

where we implicitly assume that those sums converge absolutely. This is a discrete analogue of the usual paraproduct decomposition, cf. Appendix 1. If $\omega _1,\omega _2\in \mathbb {Z}^{2}\setminus \{0\}$, then $\omega _1\sim \omega _2$ implies $9/64|\omega _1|\le |\omega _2|\le 64/9|\omega _1|$ and $\omega _1\precsim \omega _2$ implies $|\omega _1|\le 8/9|\omega _2|$.

We also define a filtered probability space $(\Omega ,\mathcal {F},(\mathcal {F}_{t})_{t\ge 0},\mathbb {P})$ with a complete, right-continuous filtration, which we assume to be large enough to support a countable family of Brownian motions. This probability space will be fixed, so that whenever a property holds almost surely, it will do so with respect to $\mathbb {P}$.

### Strategy and main result

We first outline the paracontrolled approach to (1.1) in a relatively loose manner, identifying the main steps of the method and the diagrams that we will need to give meaning to. Recall that we wish to define a sufficiently robust notion of solution to (1.1) which in particular is stable under regular approximations to the noise. To do so we first write (1.1) in mild form, setting

1.9 $$\begin{aligned} \rho =P\rho _0+\nabla \cdot \mathcal {I}[\rho \nabla \Phi _{\rho }]+\nabla \cdot \mathcal {I}[\sigma \varvec{\xi }]. \end{aligned}$$

Note that $\rho$ may blow up before time *T* due to the non-linearity on the right-hand side of (1.9). For the purpose of this discussion, we will assume that $\rho$ exists until time *T*; see Sect. 3.2 for results on the maximal time of existence. In the remainder of this section we will assume that all terms on the right hand side of (1.9) are continuous in time while taking values in a Hölder–Besov space $\mathcal {C}^{\alpha }(\mathbb {T}^2)$, where $\alpha$ is possibly negative.

Working, for now, with smooth initial data, we may assume that the final term on the right hand side is the least regular component of $\rho$. Using the same stochastic estimates alluded to in the introduction, along with the regularising effect of the heat kernel and the effect of the derivative, we will work under the assumption that . Passing this regularity to $\rho$ and applying the regularising effect of the elliptic equation we expect to have $\nabla \Phi _{\rho } \in C_T\mathcal {C}^{0-}$. Therefore, as discussed in the introduction, the product $\rho \nabla \Phi _{\rho }$ is not a priori well-defined. Our first step is to employ the so called Da Prato–Debussche trick, [14], to remove the most singular term by defining  so that if $\rho$ is a solution to (1.9),We notice that the product  is not classically well-posed, however it can be renormalised and replaced with the symbol , where  denotes the singular part of this product. The term  is well-defined and will have the regularity that one would formally expect of , namely  (see Sect. 2.4). From now on we continue with our expansion, replacing the singular product by its renormalised counterpart  so that we have in fact changed the equation solved by $\rho$.

We are now in better shape, as we may now work with $u \in C_T\mathcal {C}^{0-}$, which renders the first product on the right hand side classically well-posed. However, the second and third products remain ill-defined. We may repeat the same trick, defining , which should solve,where  denotes a finite sum of classically well-posed terms involving *w*,  and . The formal definition of Bony’s decomposition into para and resonant products is given in Appendix 1, however, for now we simply recall the rules that for $f\in \mathcal {C}^{\alpha }$, $g\in \mathcal {C}^{\beta }$, one hasThe new symbol appearing on the right hand side for *w* is a shorthand for . Although those resonant products are not classically well-defined, further stochastic arguments show that they can in fact be defined as objects finite in $C_T\mathcal {C}^{0-}$ without the subtraction of any infinite counterterms. The full definition of , through stochastic calculus, is contained in Sect. 2.2 and we show in Sects. 2.4 and 2.5 that . So even though it requires significant work to define, it is not the least regular term on the right hand side.

Instead this is given by the paraproduct term, , which using Bony’s estimate (Lemma A.4) is only finite in $C_T\mathcal {C}^{0-}$, and the formal product term , which is not even a priori well-defined. Hence, as before we can only expect to find $w\in C_T\mathcal {C}^{0-}$ which is not regular enough to define the products  and  a priori.

One sees that further applications of the Da Prato–Debussche trick will not improve the situation. Instead we employ the core idea that solutions should resemble the noise at small scales. This is formalised through the *paracontrolled Ansatz*, that is, we only look for solutions such that,1.10where the *paracontrolled remainders* $w^{\#}$ and $(\nabla \Phi _w)^{\#}$ are terms to be fixed by the equation which we stipulate to be finite in $C_T\mathcal {C}^{0+}$ and $C_T\mathcal {C}^{1+}$ respectively.^3^ This ensures that the products  and  are classically well-defined. Rearranging, using the linearity of the map $f\mapsto \nabla \Phi _f$ and applying Bony’s decomposition to the products  and , we find the identitywhere  is a new polynomial of its arguments and can be expected to be of strictly positive regularity. Hence, the regularity of $w^{\#}$ is governed by that of the commutator and that of the resonant products  and . The commutator can be controlled by Lemmas A.9 and A.10, which show thatTo treat the resonant products we make use of the *Ansatz* again, writingandUnder our stipulation that $w^{\#}\in C_T\mathcal {C}^{0+}$ and $(\nabla \Phi _w)^{\#} \in C_T\mathcal {C}^{1+}$, the final two resonant products are classically well-defined and so it only remains to check that the first term of each expansion is finite. To achieve this last step we consider a commutator for the triple product,Lemma A.11 shows that the commutator lies in $C_{T}\mathcal {C}^{1-}$. We apply a similar trick to the resonant product , writingAgain, the regularity of the commutator follows from Lemma A.11. Taken together the last two exogenous terms produce the final diagram we are required to construct,Note that the first resonant product above should be read as a vector outer product so that  is matrix valued. We would naively expect both summands of  to diverge logarithmically if we replace $\varvec{\xi }$ by $\varvec{\xi }^{\delta }$ and let $\delta \rightarrow 0$. However, the symmetry of the Green’s function allows us to show that after summing both terms,  is well-defined in a sufficiently strong topology even for $\delta =0$.

Reversing all of the above steps we find the modified equation solved by our paracontrolled object,1.11with $w^{\#}$ a solution to1.12for  a third polynomial of its arguments, their paraproducts and commutators. We call this $\rho$ a paracontrolled solution to (1.1) with enhancement $\mathbb {X}$. For a rigorous definition, see Definition 3.10.

In the paracontrolled decomposition of $\rho$, the first three terms lie in spaces of negative regularity. Hence, the singular parts of the product $\rho \nabla \Phi _{\rho }$ will be determined by non-linear combinations of the first three terms. Since  and  will be supplied as data these terms can be handled directly. However, as *w* also carries information from $\rho$, products involving  cannot be handled in the same way. Instead we make use of the commutator estimates above. To see this in practice and to identify the possibly diverging field $f^\delta$ alluded to in the introduction, we recall our notion of a mollified noise by setting , where $\psi _{\delta }$ is a standard mollifier. We use the notations , ,  to denote the same diagrams now constructed from , and use $\rho ^{\delta }$ to denote the solution of the mild equation1.13We further denote the second Da Prato–Debussche remainder by  and define .

We have the identity,1.14Here we have only kept track of terms that are either not classically well-defined or contain stochastic diagrams which require construction. The final term involving  arises from applying commutators to the paraproduct term in the expansion of $\rho ^\delta$ where the more regular parts have been left implicit above. Since we only expect to have $\rho ^\delta \rightarrow \rho$ in $C_T\mathcal {C}^{-1-}$ we do not expect (1.14) to converge directly. We have already identified the possibly diverging field which renormalises the first term, sinceAs discussed in the introduction, formal power counting would lead one to expect  to diverge at order $\delta ^{-1}$, however, exploiting the symmetry of the elliptic Green’s function we have that .

The diverging diagram  is also contained in the terms  and . However, since  is of regularity $0-$ and  of regularity $-1-$, it is not directly clear how to make sense of those products. Note that if  were a diverging constant rather than a field this would simply be scalar multiplication and we would have no trouble. Nevertheless, using that  is deterministic, one can define the products  and  directly as Itô objects and show that they diverge at a rate no worse than . We refer to Sect. 2.6 for this argument.

Since , we may expand the product  to cancel the diverging terms  and  in (1.14). Hence, we can construct the renormalised product  without further modifications. In particular, this identifies the renormalising sequence $(f^{\delta })_{\delta >0}$ of (1.5) as  and (1.13) as the corresponding mild formulation.

We have therefore identified both the solution $\rho$ and the non-linear term in (1.9) as trilinear functions of a suitable enhancement of the noise. To conclude this section we paraphrase the main result of this paper in the case of regular initial data. The complete statement, including the case of irregular initial data, and the proof are split between Theorems 2.3 and 3.22.

#### Theorem 1.2

Let $\rho _0\in \mathcal {C}^{0+}$ be some initial data, $\varvec{\xi }= (\xi ^1,\xi ^2)$ be a vector-valued space-time white noise on $[0,\infty ) \times \mathbb {T}^2$, $\sigma :[0,\infty ) \times \mathbb {T}^2\rightarrow \mathbb {R}$ be a map such that $\sigma \in C_T\mathcal {H}^2$ for some $T>0$ and $(\psi _\delta )_{\delta >0}$ be a family of symmetric mollifiers. Then there exist enhancements ,  as described above (in particular $\mathbb {X}^\delta$ is built from $\sigma \varvec{\xi }^\delta$ with $\varvec{\xi }^\delta = \psi _{\delta }*\varvec{\xi }$) and a random variable $T_{\text {max}}\in (0,T]$ (the maximal time of existence) with the following properties:

1. There exists a unique paracontrolled solution $\rho$ to (1.1) on $[0,T_{\text {max}})$ with enhancement $\mathbb {X}$ and initial data $\rho _{0}$, which we call the renormalised solution. The maximal time of existence $T_{\text {max}}$ satisfies
  $$\begin{aligned} T_{\text {max}}=T\qquad \text {or}\qquad \lim _{t\uparrow T_{\text {max}}}\Vert \rho (t)\Vert _{\mathcal {C}^{-1-}}=\infty \qquad \text {almost surely}. \end{aligned}$$
2. For each $$\lambda >0$$,
  $$\begin{aligned} \lim _{\delta \rightarrow 0}\mathbb {P}\Big (\sum _{L=1}^{\infty }\frac{2^{-L}}{L}\frac{\Vert \rho -\rho ^{\delta }\Vert _{C_{T_{L}}\mathcal {C}^{-1-}}}{1+\Vert \rho -\rho ^{\delta }\Vert _{C_{T_{L}}\mathcal {C}^{-1-}}}>\lambda \Big )=0, \end{aligned}$$
  where $\rho ^{\delta }$ is the solution to (1.13) and
  $$\begin{aligned} T_{L}{:}{=}T\wedge L\wedge \inf \{t\in [0,T]:\Vert \rho (t)\Vert _{\mathcal {C}^{-1-}}>L~\text {or}~\Vert \rho ^{\delta }(t)\Vert _{\mathcal {C}^{-1-}}>L\} \, \mathrm{for~ every}~ L\in \mathbb {N}. \end{aligned}$$

Furthermore . If $\sigma$ is a constant then  while in general one has the bound .

#### Remark 1.3

In the case of constant $\sigma$ it also holds that . This is due to the symmetry of the elliptic Green’s function, see the discussion of (1.6).

## Noise enhancement

In this section we construct the enhancements required in Theorem 1.2 and establish their regularities.

### Outline and regularities

We begin by defining a vector $\varvec{\xi }=(\xi ^1,\xi ^2)$ of space-time white noises as in [44]. Let $(W^{j}(\cdot ,m))_{m\in \mathbb {Z}^2,j=1,2}$ be a family of complex-valued Brownian motions on $\mathbb {R}_{+}$ starting from 0 that satisfy $\overline{W^j(\cdot ,m)}=W^j(\cdot ,-m)$ and are otherwise independent.

We define for $j=1,2$, the space-time white noise $\xi ^j$ by setting for any $\phi \in L^2((0,\infty )\times \mathbb {T}^2;\mathbb {C})$,

2.1 $$\begin{aligned} \xi ^{j}(\phi ){:}{=}\sum _{m_1\in \mathbb {Z}^2}\int _{0}^{\infty } \textrm{d}W^j(u_1,m_1){\widehat{\phi }}(u_1,-m_1). \end{aligned}$$

We define our choice of mollifiers.

#### Definition 2.1

Let $\varphi \in C^{\infty }(\mathbb {R}^2)$ be of compact support, $\text {supp}(\varphi )\subset B(0,1)$, even and such that $\varphi (0)=1$. Given $\varphi$, we define a sequence of mollifiers $(\psi _\delta )_{\delta >0}$ by $\psi _{\delta }(x){:}{=}\sum _{\omega \in \mathbb {Z}^{2}}\textrm{e}\hspace{0.3888pt}^{2\pi \textrm{i}\hspace{0.3888pt}\langle \omega ,x\rangle }\varphi (\delta \omega )$.

We define a space of enhanced noises.

#### Definition 2.2

*(Enhanced rough noise)* Let $T>0$, $\alpha \in (-5/2,-2)$ and $\kappa \in (0,1/2)$ and let the map

$$\begin{aligned} \Theta :(\mathscr {L}^{\kappa }_T\mathcal {C}^{\alpha +2}\times \mathscr {L}^{\kappa }_T\mathcal {C}^{2\alpha +5})&\rightarrow \mathscr {L}^{\kappa }_T\mathcal {C}^{\alpha +1}\times \mathscr {L}^{\kappa }_T\mathcal {C}^{2\alpha +4}\times \mathscr {L}^{\kappa }_T\mathcal {C}^{3\alpha +6}\times \mathscr {L}^{\kappa }_T\mathcal {C}^{2\alpha +4},\\ (v,f)&\mapsto \Theta (v,f), \end{aligned}$$

be given byWe define the space $\mathcal {X}^{\alpha ,\kappa }_T$ to be the closure of the subset

$$\begin{aligned}  &   \{\Theta (v,f):(v,f)\in \mathscr {L}^{\kappa }_T\mathcal {C}^{\alpha +2}\times \mathscr {L}^{\kappa }_T\mathcal {C}^{2\alpha +5},~v_{0}=f_{0}=0\}\\  &   \quad \subset \mathscr {L}^{\kappa }_T\mathcal {C}^{\alpha +1}\times \mathscr {L}^{\kappa }_T\mathcal {C}^{2\alpha +4}\times \mathscr {L}^{\kappa }_T\mathcal {C}^{3\alpha +6}\times \mathscr {L}^{\kappa }_T\mathcal {C}^{2\alpha +4}. \end{aligned}$$

We shall denote a generic element of this closure by  and equip it with the metric induced by the norm

The main result of this section is the following theorem, reminiscent of [24, Thm. 9.1].

#### Theorem 2.3

Let $T>0$ and $\sigma \in C_T\mathcal {H}^{2}$. Let $\varvec{\xi }$ be a two-dimensional vector of space-time white noises, $(\psi _{\delta })_{\delta >0}$ be a sequence of mollifiers as in Definition 2.1, $\varvec{\xi }^{\delta }{:}{=}\psi _{\delta }*\varvec{\xi }{:}{=}(\psi _{\delta }*\xi ^{1},\psi _{\delta }*\xi ^{2})$ and  be given byThen for all $\alpha \in (-5/2,-2)$ and $\kappa \in (0,1/2)$, the following hold

1. Almost surely, $\mathbb {X}^{\delta }\in \mathcal {X}^{\alpha ,\kappa }_{T}$ and
  $$\begin{aligned} \mathbb {X}^\delta \in \mathscr {L}^{\kappa }_{T}\mathcal {C}^{\alpha +2}\times \mathscr {L}^{\kappa }_{T}\mathcal {C}^{2\alpha +5}\times (\mathscr {L}^{\kappa }_{T}\mathcal {C}^{3\alpha +8})^{\times 2}\times (\mathscr {L}^{\kappa }_{T}\mathcal {C}^{2\alpha +6})^{\times 4}. \end{aligned}$$
2. Almost surely, there exists some , such that for any $p\in [1,\infty )$ we have $\lim _{\delta \rightarrow 0}\mathbb {E}(\Vert \mathbb {X}-\mathbb {X}^{\delta }\Vert _{\mathcal {X}^{\alpha ,\kappa }_{T}}^p)^{1/p}=0$ and $\mathbb {E}(\Vert \mathbb {X}\Vert _{\mathcal {X}^{\alpha ,\kappa }_{T}}^p)^{1/p}<\infty$.
3. Defining  it holds that  and for any $p\in [1,\infty )$,

An explicit definition of the limit  can be found in Sect. 2.2. We call $\mathbb {X}$ the *renormalised enhancement* and  the *canonical enhancement*.

The result will be shown in several parts, namely in Lemma 2.7 (), Lemma 2.16 (), Lemma 2.19 and Lemma 2.24 (), Lemma 2.20 and Lemma 2.25 () and Lemma 2.26 (, , ).

#### Remark 2.4

Different aspects of Theorem 2.3 require different assumptions on the heterogeneity $\sigma$. For example the regularity of  and  only requires $\sigma \in C_TL^\infty$ (Lemmas 2.7 and 2.16) while the regularities of the contractions contained in ,  require that uniformly over $t\in [0,T]$ one has $\sup _{\omega \in \mathbb {Z}^2}|{\widehat{\sigma }}(t,\omega )|(1+|\omega |^2) <\infty$. The assumption $\sigma \in C_T\mathcal {H}^2$ implies both of these conditions and provides a convenient norm and well-studied space that controls the latter quantity; hence we choose to work with this simpler, if sub-optimal restriction. Furthermore, with a view to setting $\sigma = \sqrt{\rho _{\text {det}}}$ (cf. (1.3)) the condition $\sigma \in C_T\mathcal {H}^2$ is more straightforward to check.

#### Remark 2.5

As discussed in the introduction, we build our *regular* enhancement from $\sigma \varvec{\xi }^\delta$ instead of $(\sigma \varvec{\xi })^\delta$ and with only a spatial convolution. Hence, for  to be more regular, we need to assume some regularity on $\sigma$. We again make use of the condition $\sup _{t\in [0,T]}\sup _{\omega \in \mathbb {Z}^{2}}|{\widehat{\sigma }}(t,\omega )|(1+|\omega |^{2})<\infty$ (see the proof of Lemma 2.7), though other choices are possible.

#### Remark 2.6

Our methods also allow us to establish that . However, since we do not make use of the additional time regularity, we omit the proof.

In the remainder of this section, we outline the basic arguments involved in proving Theorem 2.3. We motivate the definition of  and establish its existence.

It is well-known that the Fourier frequencies of the stochastic heat equation are given by Ornstein–Uhlenbeck processes. Similarly, we can find an expression for the Fourier transform of . Let $H_{t}^j(\omega ){:}{=}2\pi \textrm{i}\hspace{0.3888pt}\omega ^j\exp (-t|2\pi \omega |^2)\mathbb {1}_{t\ge 0}$, for all $\omega \in \mathbb {Z}^2$ and $t\in \mathbb {R}$, be the multiplier appearing in $\partial _{j}\mathcal {I}$. We define  by applying the inverse Fourier transform to the sequence2.2We also introduce the Fourier transform of $\tau {:}{=}\nabla \cdot \mathcal {I}[\varvec{\xi }]$ by

$$\begin{aligned} {\widehat{\tau }}(t,\omega ){:}{=}\sum _{j_1=1}^{2}\int _{0}^{t}\textrm{d}W^{j_1}(u_1,\omega )H_{t-u_1}^{j_1}(\omega ). \end{aligned}$$

#### Lemma 2.7

Let $T>0$, $\alpha <-2$, $\kappa \in (0,1/2)$ and $\sigma \in C_{T}L^{\infty }$. Then, for any $p\in [1,\infty )$ we have  and in particular  a.s. Assume in addition $\sigma \in C_{T}\mathcal {H}^{2}$ and $\delta >0$, then it holds that  and in particular  a.s. What is more,  for any $p\in [1,\infty )$.

#### Proof

Let $\gamma \in (0,1]$, $\varepsilon \in (0,\gamma /2)$ and $\max \{1/\varepsilon ,2\}<p<\infty$. To establish the existence and regularity of  in a Besov space, we apply Nelson’s estimate (Lemma 2.14), Kolmogorov’s continuity criterion (Lemma 2.15) and the Besov embedding (A.1). Therefore, in order to establish that  almost surely, it suffices to control the quantityUsing that $\sigma \in C_{T}L^{\infty }$ is bounded, we can pass to real space with (2.7) to deduceUsing that $\mathbb {E}({\widehat{\tau }}(t,\omega )\overline{{\widehat{\tau }}(s,\omega ')})=0$ if $\omega \ne \omega '\in \mathbb {Z}^2$, we obtain

$$\begin{aligned} \begin{aligned}&\mathbb {E}(|\Delta _{q}\tau (t,x)-\Delta _{q}\tau (s,x)|^2)\\&=\sum _{\omega \in \mathbb {Z}^{2}}\sum _{\omega '\in \mathbb {Z}^{2}}\textrm{e}\hspace{0.3888pt}^{2\pi \textrm{i}\hspace{0.3888pt}\langle \omega ,x\rangle }\textrm{e}\hspace{0.3888pt}^{-2\pi \textrm{i}\hspace{0.3888pt}\langle \omega ',x\rangle }\varrho _{q}(\omega )\varrho _{q}(\omega ')\mathbb {E}(({\widehat{\tau }}(t,\omega )-{\widehat{\tau }}(s,\omega ))\overline{({\widehat{\tau }}(t,\omega ')-{\widehat{\tau }}(s,\omega '))})\\&=\sum _{\omega \in \mathbb {Z}^{2}}\varrho _{q}(\omega )^{2}\mathbb {E}(|{\widehat{\tau }}(t,\omega )-{\widehat{\tau }}(s,\omega )|^{2}). \end{aligned} \end{aligned}$$

It follows by Itô’s isometry and interpolation (A.2),

$$\begin{aligned} \mathbb {E}(|{\widehat{\tau }}(t,\omega )-{\widehat{\tau }}(s,\omega )|^2)\le \sum _{j_1=1}^{2}\int _{-\infty }^{\infty }\textrm{d}u_1|H^{j_1}_{t-u_1}(\omega )-H^{j_1}_{s-u_1}(\omega )|^2\lesssim |t-s|^{\gamma }|\omega |^{2\gamma } \end{aligned}$$

and therefore uniformly in $x\in \mathbb {T}^{2}$,

$$\begin{aligned} \mathbb {E}(|\Delta _{q}\tau (t,x)-\Delta _{q}\tau (s,x)|^2)\lesssim |t-s|^\gamma 2^{q(2+2\gamma )}. \end{aligned}$$

To summarize, we obtain by Lemmas 2.14, 2.15 and the arguments above for each $\max \{1/\varepsilon ,2\}<p<\infty$,2.3which we can generalize to $p\in [1,\infty )$ by Hölder’s inequality. In particular,  almost surely, where $\varepsilon \in (0,\gamma /2)$ can be chosen arbitrarily small.

Next we show that the approximating sequence  is more regular if $\sigma \in C_{T}\mathcal {H}^{2}$. In Fourier space,2.4We apply Itô’s isometry, the triangle inequality and interpolation (A.2) to estimatewhere we used that $|{\widehat{\sigma }}(u,\omega )|\lesssim (1+|\omega |^{2})^{-1}\Vert \sigma \Vert _{C_{T}\mathcal {H}^{2}}$ uniformly in $u\in [0,T]$ and $\omega \in \mathbb {Z}^{2}$, and that $(1+x^2)^{1/2}|\varphi (x)|\lesssim \Vert \varphi \Vert _{L^{\infty }}$ uniformly in $x\in \mathbb {R}^{2}$ (the latter of which we leave implicit.)

We may assume $\omega ,\omega '\in \mathbb {Z}^{2}\setminus \{0\}$, since . We decompose the sum over $m_1\in \mathbb {Z}^{2}$ into the domains $m_1=0$, $m_1=\omega$, $m_1=\omega '$ and $m_1\in \mathbb {Z}^{2}\setminus \{0,\omega ,\omega '\}$,

$$\begin{aligned} \begin{aligned}&\sum _{m_1\in \mathbb {Z}^{2}}(1+|\omega -m_1|^{2})^{-1}(1+|\omega '-m_1|^{2})^{-1}(1+|\delta m_1|^{2})^{-1}\\&\le |\omega |^{-2}|\omega '|^{-2}+\delta ^{-2}(1+|\omega -\omega '|^{2})^{-1}(|\omega |^{-2}+|\omega '|^{-2})\\&\quad +\delta ^{-2}\sum _{m_1\in \mathbb {Z}^{2}\setminus \{0,\omega ,\omega '\}}|\omega -m_1|^{-2}|\omega '-m_1|^{-2}|m_1|^{-2}. \end{aligned} \end{aligned}$$

We estimate the sum over $m_1\in \mathbb {Z}^{2}\setminus \{0,\omega ,\omega '\}$. Assume $\omega =\omega '$, then by Lemma C.5 and (C.3),

$$\begin{aligned} \sum _{m_1\in \mathbb {Z}^{2}\setminus \{0,\omega ,\omega '\}}|\omega -m_1|^{-4}|m_1|^{-2}\lesssim |\omega |^{-2}. \end{aligned}$$

Assume $\omega \ne \omega '$, we apply Lemma C.4 to estimate for $\varepsilon <1$,

$$\begin{aligned} \sum _{m_1\in \mathbb {Z}^{2}\setminus \{0,\omega ,\omega '\}}|\omega -m_1|^{-2}|\omega '-m_1|^{-2}|m_1|^{-2}\lesssim |\omega -\omega '|^{-2+\varepsilon }(|\omega |^{-2+2\varepsilon }+|\omega '|^{-2+2\varepsilon }). \end{aligned}$$

Having established the decay of the Fourier coefficients, we can bound the Littlewood–Paley blocks. Let $q\in \mathbb {N}_{-1}$, we obtain uniformly in $x\in \mathbb {T}^{2}$,Consequently, by Lemmas 2.14 and 2.15 for any $p\in [1,\infty )$ and $\varepsilon \in (0,\gamma /2)$,In particular,  almost surely, where $\varepsilon \in (0,\gamma /2)$ can be chosen arbitrarily small.

To deduce  for all $p\in [1,\infty )$, $\alpha <-2$ and $\kappa \in (0,1/2)$, let $\gamma \in (0,1]$, $\varepsilon \in (0,\gamma /2)$, $\max \{1/\varepsilon ,2\}<p<\infty$ and $0<\vartheta '<\vartheta \le 1$. We use Nelson’s estimate (Lemma 2.14) and Kolmogorov’s continuity criterion (Lemma 2.15) to boundWe then use Lemma 2.12 to boundUsing the same estimates as in the derivation of (2.3), we obtainTherefore,which implies . An application of Hölder’s inequality allows us to deduce the convergence for all $p\in [1,\infty )$, where $\varepsilon$ and $\vartheta$ can be chosen arbitrarily small. This yields the claim.

$$\square $$

The convergence of the other approximations in $\mathbb {X}^{\delta }$ is analogous. The only crucial observation here is that we can choose $\vartheta '$ and $\vartheta$ arbitrarily small when applying Lemma 2.12, which ensures that the various applications of Lemma C.3 in the proof of Theorem 2.3 carry over. Hence, we will omit the details.

### Feynman diagrams

As demonstrated by (2.2), we may construct our white-noise enhancement as (iterated) stochastic integrals. However, as we continue to multiply terms, we need to apply Itô’s product rule to increasingly complicated expressions. To implement this procedure efficiently, we use an extension of a graphical representation that was developed by [24, 44], which relates our stochastic objects to Feynman diagrams.

There are several types of vertices. A root  represents the argument $(t,\omega )$. A circle  denotes an instance of stochastic integration in time against a two-dimensional Brownian field with heterogeneity $\sigma$. Graphically this integrator is given by 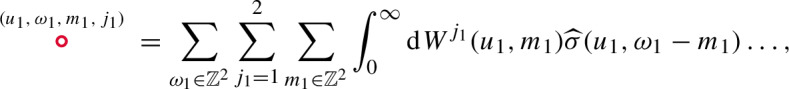 where the placeholder $\ldots$ stands for an integrand in $u_1$, $\omega _1$, $m_1$, $j_1$, which is to be determined from the remaining diagram.

Generally, circles are enumerated by $k\in \mathbb {N}$ and equipped with tuples of dummy variables $(u_k,\omega _k,m_k,j_k)$, where $u_k\in (0,\infty )$, $\omega _k\in \mathbb {Z}^{2}$, $m_k\in \mathbb {Z}^{2}$ and $j_k=1,2$, which we point out explicitly for the reader’s convenience. We refer to the $\omega$, $\omega _k$ as the *frequencies* and the $m_k$ as the *modes*. We denote coordinates of those by superscripts.

Vertices are connected by different types of directed edges, i.e. arrows, representing integrands. Black arrows  are associated to the integrand $H_{t-u_k}^{j_k}(\omega _k)$, which is the Fourier multiplier appearing in $\nabla \cdot \mathcal {I}$. Highlighted arrows , for $j=1,2$, are associated to $G^j(\omega _k)H_{t-u_k}^{j_k}(\omega _k)$, where

$$\begin{aligned} G^j(\omega _k){:}{=}2\pi \textrm{i}\hspace{0.3888pt}\omega _k^j|2\pi \omega _k|^{-2}\mathbb {1}_{\omega _k\ne 0} \end{aligned}$$

is the multiplier for $\partial _j\Phi$. The direction of an arrow indicates the smaller time variable $u_k$ in the integration. Furthermore, if a single arrow emerges from the root , we multiply the integrand by $\mathbb {1}_{\omega =\omega _{1}}$.

For example, by applying those rules, we can represent (2.2) as 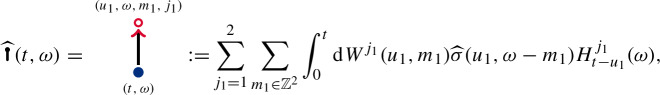 and  as 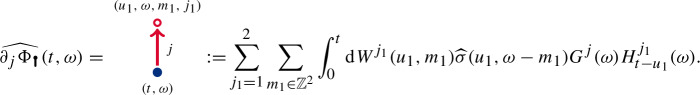 In particular, if $\omega =0$, then $H^j_t(\omega )=G^j(\omega )=0$, hence we may assume $\omega \ne 0$ whenever it appears in either multiplier.

As a general rule, in our diagrams **black** objects are associated to scalars and objects are associated to vectors. Our arrows have highlighted arrowheads, indicating that they act on vector-valued objects. On the other hand, the type of object they return is determined by the arrow shaft. Accordingly,  produces a scalar and  a vector.

The existence of  is not guaranteed by Lemma A.4, since  and hence . In order to construct such non-linear objects, we formally apply Itô’s product rule to identify the candidate Fourier transform. Let $n\in \mathbb {N}$ and assume $a_1,\ldots ,a_n\in \mathbb {N}$ are distinct. We denote by $\Sigma (a_1,\ldots ,a_n)$ the permutation group of $\{a_1,\ldots ,a_n\}$. Let $\omega _1,\omega _2\in \mathbb {Z}^2$, we compute2.5The symmetrization of the first integrand is a direct consequence of the Itô product rule. Note that in the second term, $j_1=j_2$, $u_1=u_2$ but $m_1=-m_2$, which is a consequence of the Hermitean structure of complex Brownian motion. Such a decomposition of stochastic products into iterated (stochastic) integrals is often called a *Wiener chaos decomposition* after [53], see [27, 44] for more details.

To represent , let us extend our graphical rules. Two arrows emerging from a common vertex represent a convolution in Fourier space. Their integrands are multiplied, but are related by the *Kirchhoff rule* [44]: each vertex *v* has a frequency $\omega$ or $\omega _k$ which is part of its variables. This frequency will be called *ingoing* at the vertex *v*. An ingoing frequency at a vertex *v* is *outgoing* for the vertex *w*, if there exists an arrow pointing from *w* to *v*. A vertex is called *internal*, if there exists an arrow emerging from it. The rule states that at each internal vertex, the ingoing frequency (e.g. $\omega$ above) equals the sum of the outgoing frequencies (e.g. $\omega _1$, $\omega _2$ above). In graphical notation, 
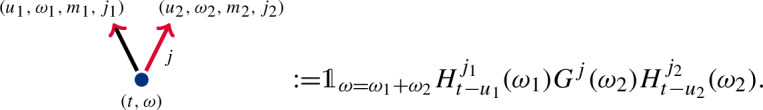
Those arrows will target the integrators  and  which will be multiplied and integrated over. The integral is then restricted to the simplex $u_1<u_2$ to ensure that the integrand is adapted. To obtain the integral over the full domain, we symmetrize the integrand by permuting the indices that appear in the simplex. For example, 
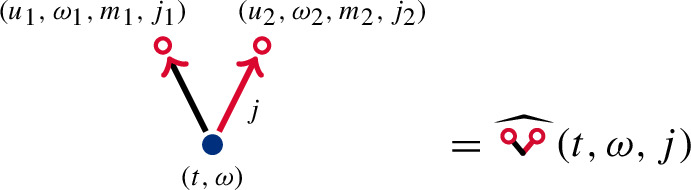
 is the first object in the decomposition (2.5).

Next, let us discuss . As can be seen in (2.5), instances of Lebesgue integration arise through Itô correction terms. Itô corrections will be denoted by *contractions*, i.e. two arrows pointing at different vertices  and  are merged at a common vertex . Graphically, 
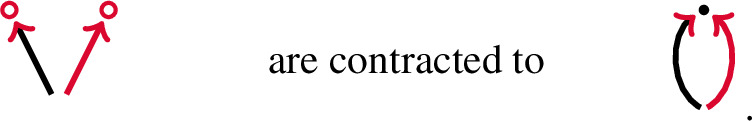
Using the orthogonality of $(W^{j}(u,m))_{u\ge 0,m\in \mathbb {Z}^{2},j=1,2}$, we can identify some of the dummy variables of the two vertices that are being merged. Indeed, as in (2.5), we set $j_1=j_2$, $u_1=u_2$, $m_1=-m_2$, but leave $\omega _1$, $\omega _2$ as is. To make it easier for the reader to discern the multipliers attached to each arrow, we give both tuples of dummy variables, even after the contraction. Graphically, 
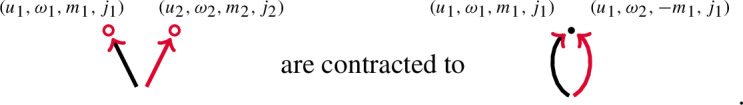
 This results in the diagram 
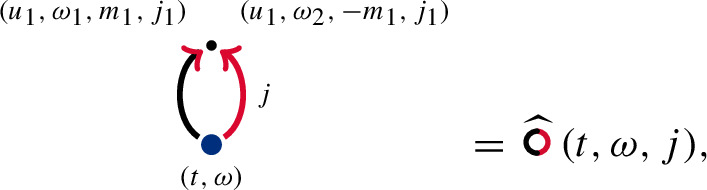
 which is the Itô correction in the decomposition (2.5) and coincides with the mean, ). Diagrams carrying contractions are often called *Wick contractions* after [52], see [24] for more details.

Depending on $\sigma$,  may be infinite. Hence, we consider the *renormalised enhancement*, where we only keep the first term  of the decomposition (2.5) to define . Formally, this is equivalent to subtracting the mean as a counterterm,This identity can be made rigorous with suitable regularization and limit procedures, see the discussion of the canonical enhancement at the end of this section.

Another source of Lebesgue integrals is the concatenation of the $\mathcal {I}$ operation. We obtain arrows pointing at other arrows, connected through a vertex . We multiply their integrands and make sure to respect the Kirchhoff rule. The multiplier of the incoming arrow will be determined by a tuple of dummy variables $(u_k,\omega _k,j_k)$ at the connecting vertex. For example,  can be expressed as 
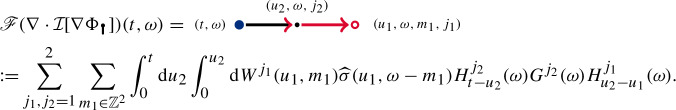
The renormalised stochastic object  can then be expressed as 
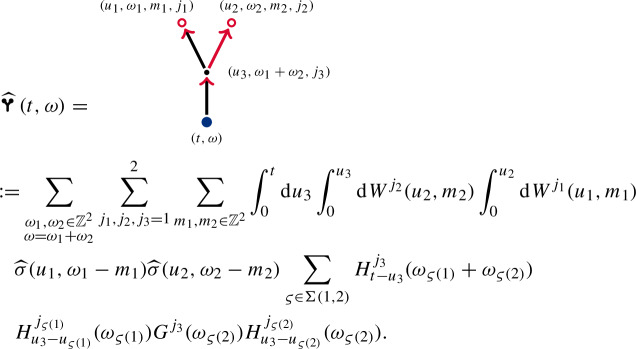


In fact, we will not construct  in itself. As we will see in Lemma 2.16,  can be constructed as a continuous function in time that takes values in a space of distributions. On the other hand, we do not expect  to admit pointwise-in-time values. Instead, we expect it to exist as a proper space-time distribution. This resembles the situation discussed in [44, pp. 23–24 & pp. 32–33] and [9, 28].

A particular variant of the root  is the vertex  which arises through applications of the resonant product. The vertex  relates the frequencies of the arrows that it joins through the $\sim$-relation defined in (1.7), see also [44, (64)].

Let us consider more complicated objects. We have the Wiener chaos decompositionThe contractions  and  that one might expect are absent in the renormalised enhancement due to our definition of . We express the third-order Wiener chaos term  as follows. For $j=1,2$, 
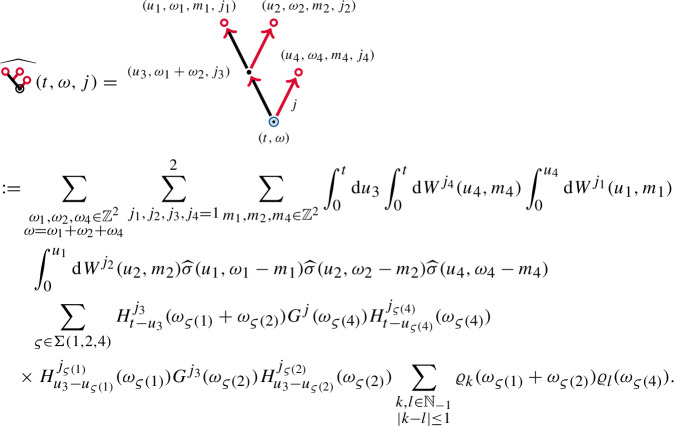
 The diagrams  and , may not exist in themselves, but the summed object  does. Its iterated integral representation is given by
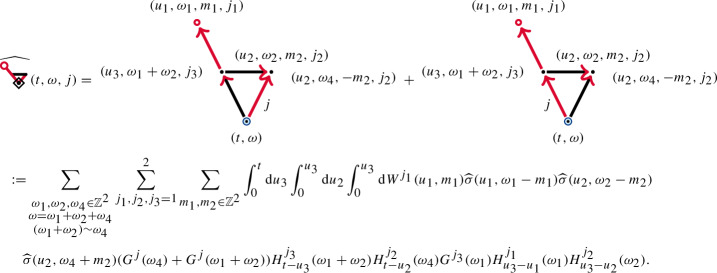
 The remaining diagrams ,  and  are similar to the ones given above.

We will show in Lemma C.1 that the resulting factor $G^{j}(\omega _4)+G^{j}(\omega _1+\omega _2)$ has better decay in $\omega _4$, than $G^{j}(\omega _4)$. This is due to the symmetry of $G^{j}$, which allows us to write $G^{j}(\omega _1+\omega _2)+G^{j}(\omega _4)=G^{j}(\omega -\omega _4)-G^{j}(-\omega _4)$. The improved decay leads to the well-posedness of  and is a higher-dimensional analogue of the product rule discussed in (1.6).

#### Remark 2.8

One could simplify the contraction by using the identity

2.6 $$\begin{aligned} \sum _{m_2\in \mathbb {Z}^{2}}{\widehat{\sigma }}(u_2,\omega _2-m_2){\widehat{\sigma }}(u_2,\omega _4+m_2)=\widehat{\sigma ^{2}}(u_2,\omega _2+\omega _4). \end{aligned}$$

This idea would allow us to derive bounds in terms of $\Vert \sigma ^2\Vert _{C_T\mathcal {H}^{2}}$ rather than $\Vert \sigma \Vert _{C_T\mathcal {H}^{2}}^{2}$. However, (2.6) is no longer applicable in our prelimiting enhancement due to the cut-off $\varphi (\delta m_2)$ (cf. (2.4)). Instead we use direct estimates that do not rely on (2.6).

We extend our graphical rules to incorporate the operator $\partial _{l}\mathcal {I}$ for $l=1,2$. An indexed black arrow pointing at a scalar object  is associated to the multiplier $H^{l}_{t-u_k}(\omega _k)$. On the other hand, a doubly-indexed, highlighted arrow pointing at a scalar object  is associated to $G^{j}(\omega _k)H^{l}_{t-u_k}(\omega _k)$.

The remaining object in the enhancement is  for $k,j=1,2$. We consider the Wiener chaos decompositionThe first term is given by 
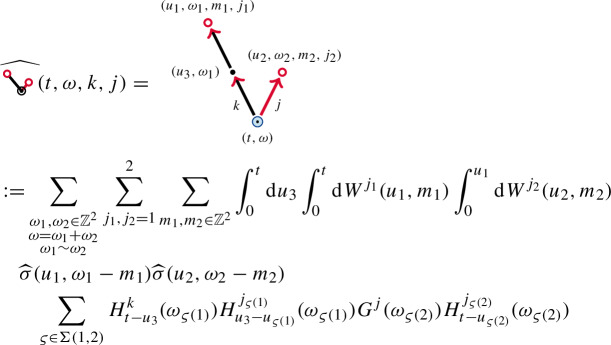
 and the second term  is again similar.

We consider the contractions as a summed object . We obtain
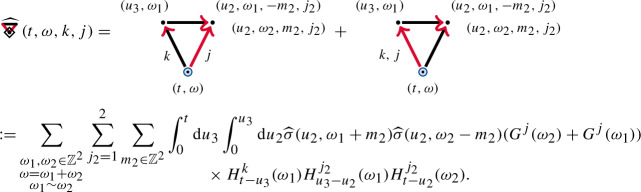
 We can define approximate diagrams as in (2.4) by multiplying the cut-off $\varphi (\delta m_k)$ to each instance of the noise .

In general, we denote the regularization of a diagram by a superscript $\delta$. The canonical enhancement  is then built from regularized noise terms, but retains the diverging sequences that are removed in the renormalised enhancement $\mathbb {X}$. Repeating (2.5), we may consider the decomposition of the diagram with cut-off,where . In addition to , we also have to control the mean, 
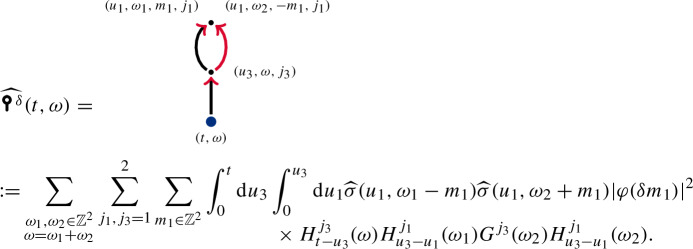
 Including  in  generates additional terms in the decomposition of ,The diagram  is given by 
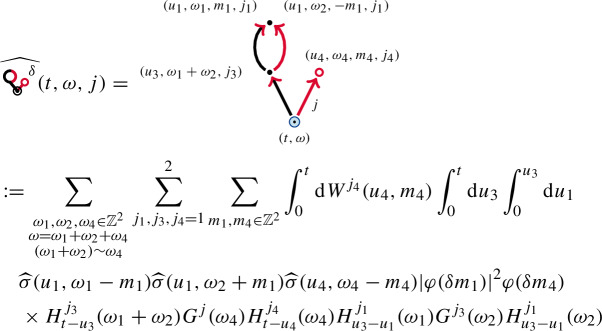
and  is again similar. Here, we have implicitly changed our graphical rules to include the cut-off.

### Existence and regularity of stochastic objects

In this section we define the notion of an iterated Itô integral with heterogeneity $\sigma$ and discuss in Lemma 2.11 how it can be controlled by passing to real space. We then use an interpolation argument in Lemma 2.12 to show that mollified iterated Itô integrals, where we include a cut-off on each mode, converge to their un-mollified counterparts. Subsequently, we introduce Nelson’s estimate, Lemma 2.14, and derive a general criterion of existence for stochastic objects taking values in Besov spaces, Lemma 2.15. See also [24, 44] for different instances of the same arguments.

Let $n\in \mathbb {N}$, $D\subset (0,\infty )^{n}$ and $\phi \in L^2(D\times \mathbb {T}^{2n}\times \{1,2\}^{n};\mathbb {C})$. We define the spatial Fourier transform of $\phi$ by

$$\begin{aligned}  &   {\widehat{\phi }}(u_1,\omega _1,j_1,\ldots ,u_n,\omega _n,j_n)\\  &   \quad {:}{=}\int _{(\mathbb {T}^2)^n}\textrm{e}\hspace{0.3888pt}^{-2\pi \textrm{i}\hspace{0.3888pt}(\langle \omega _1,x_1\rangle +\ldots +\langle \omega _n,x_n\rangle )}\phi (u_1,x_1,j_1,\ldots ,u_n,x_n,j_n)\textrm{d}x_1\ldots \textrm{d}x_n. \end{aligned}$$

We can now define iterated Itô integrals.

#### Definition 2.9

Let $n\in \mathbb {N}$ and let

$$\begin{aligned} (0,\infty )^{n}_{>}{:}{=}\{(u_{1},\ldots ,u_{n})\in (0,\infty )^{n}:u_{1}>u_{2}>\ldots >u_{n}\}. \end{aligned}$$

We define the iterated Itô integral acting on $\phi \in L^2((0,\infty )^n_{>}{\times }\mathbb {T}^{2n}{\times }\{1,2\}^{n};\mathbb {C})$ by

$$\begin{aligned}  &   I^{n}(\phi ){:}{=}\sum _{\omega _1,\ldots ,\omega _n\in \mathbb {Z}^2}\sum _{j_1,\ldots ,j_n=1}^{2}\int _{0}^{\infty }\textrm{d}W^{j_1}(u_1,\omega _1)\ldots \int _{0}^{u_{n-1}}\textrm{d}W^{j_n}(u_n,\omega _n)\\  &   \quad {\widehat{\phi }}(u_1,-\omega _1,j_1,\ldots ,u_n,-\omega _n,j_n). \end{aligned}$$

We can identify second moments of iterated Itô integrals by a version of Itô’s isometry.

#### Lemma 2.10

(Itô’s isometry) Let $n\in \mathbb {N}$ and $\phi \in L^2((0,\infty )^n_{>}\times \mathbb {T}^{2n}\times \{1,2\}^{n};\mathbb {C})$, then

$$\begin{aligned} \begin{aligned} \mathbb {E}(|I^{n}(\phi )|^{2})&=\sum _{\omega _1,\ldots ,\omega _n\in \mathbb {Z}^2}\sum _{j_1,\ldots ,j_n=1}^{2}\int _{0}^{\infty }\textrm{d}u_{1}\ldots \int _{0}^{u_{n-1}}\textrm{d}u_{n}|{\widehat{\phi }}(u_1,-\omega _1,j_1,\ldots ,u_n,-\omega _n,j_n)|^{2}\\&=\Vert \phi \Vert _{L^2((0,\infty )^n_{>}\times \mathbb {T}^{2n}\times \{1,2\}^{n};\mathbb {C})}^{2}. \end{aligned} \end{aligned}$$

#### Proof

The claim follows by Itô’s isometry, applied inductively to each stochastic integral. $\square$

Next we define iterated Itô integrals with heterogeneity $\sigma$ and show how one may control the influence of the heterogeneity by passing to real space.

#### Lemma 2.11

Let $n\in \mathbb {N}$, $T>0$, $\sigma \in C_TL^{\infty }$, $\delta \ge 0$ and $\varphi$ be as in Definition 2.1. Let

$$\begin{aligned} (0,T)^{n}_{>}{:}{=}\{(u_1,\ldots ,u_n)\in (0,T)^{n}:u_{1}>u_{2}>\ldots >u_{n}\}. \end{aligned}$$

We define the heterogeneous iterated Itô integral acting on $\phi \in L^2((0,T)^n_{>}\times \mathbb {T}^{2n}\times \{1,2\}^{n};\mathbb {C})$ (with mollification if $\delta >0$) by

$$\begin{aligned} \begin{aligned}&I^{n}_{\delta ;\sigma }(\phi ){:}{=}\sum _{\omega _1,\ldots ,\omega _n\in \mathbb {Z}^2}\sum _{j_1,\ldots ,j_n=1}^{2}\sum _{m_1,\ldots ,m_n\in \mathbb {Z}^2}\int _{0}^{T}\textrm{d}W^{j_1}(u_1,m_1)\int _{0}^{u_1}\textrm{d}W^{j_2}(u_2,m_2)\ldots \\&\qquad \int _{0}^{u_{n-1}}\textrm{d}W^{j_n}(u_n,m_n)\varphi (\delta m_1)\dots \varphi (\delta m_n){\widehat{\sigma }}(u_1,\omega _1-m_1)\dots {\widehat{\sigma }}(u_n,\omega _n-m_n)\\&\qquad {\widehat{\phi }}(u_1,-\omega _1,j_1,\ldots ,u_n,-\omega _n,j_n). \end{aligned} \end{aligned}$$

If $$\delta =0$$, then

2.7 $$\begin{aligned} \mathbb {E}(|I^{n}_{0;\sigma }(\phi )|^2)\le \Vert \sigma \Vert _{C_TL^\infty }^{2n}\mathbb {E}(|I^{n}(\phi )|^2). \end{aligned}$$

If $$\delta >0$$, then

2.8 $$\begin{aligned} \mathbb {E}(|I^{n}_{\delta ;\sigma }(\phi )|^2)\le \Vert \varphi \Vert _{L^{\infty }}^{2n}\Vert \sigma \Vert _{C_TL^\infty }^{2n}\mathbb {E}(|I^{n}(\phi )|^2). \end{aligned}$$

#### Proof

Let $\varvec{\omega }=(\omega _1,\ldots ,\omega _n)$, $\varvec{m}=(m_1,\ldots ,m_n)$, $\varvec{u}=(u_1,\ldots ,u_n)$ and $\varvec{j}=(j_1,\ldots ,j_n)$. We represent

$$\begin{aligned}  &   {\widehat{\sigma }}(u_1,\omega _1-m_1)\dots {\widehat{\sigma }}(u_n,\omega _n-m_n){\widehat{\phi }}(u_1,-\omega _1,j_1,\ldots ,u_n,-\omega _n,j_n)\\  &   \quad =\widehat{\sigma ^{\otimes n}}(\varvec{u},\varvec{\omega }-\varvec{m}){\widehat{\phi }}(\varvec{u},-\varvec{\omega },\varvec{j}). \end{aligned}$$

Using that the Fourier transform turns products of $L^2(\mathbb {T}^{2n})$-functions into convolutions, we obtain

$$\begin{aligned} \sum _{\varvec{\omega }\in (\mathbb {Z}^{2})^{\times n}}\widehat{\sigma ^{\otimes n}}(\varvec{u},\varvec{\omega }-\varvec{m}){\widehat{\phi }}(\varvec{u},-\varvec{\omega },\varvec{j})=\mathscr {F}(\sigma ^{\otimes n}\phi )(\varvec{u},-\varvec{m},\varvec{j}) \end{aligned}$$

and consequently, $I^{n}_{\delta ;\sigma }(\phi )=I^{n}(\psi _{\delta }^{\otimes n}*(\sigma ^{\otimes n}\phi ))$ for $\delta >0$ and $I^{n}_{0;\sigma }(\phi )=I^{n}(\sigma ^{\otimes n}\phi )$ for $\delta =0$. To establish (2.8) for $\delta >0$, we apply Itô’s isometry (Lemma 2.10), Parseval’s theorem and the boundedness of $\varphi$ and $\sigma$ to control

$$\begin{aligned}  &   \!\!\!\! \mathbb {E}(|I^n_{\delta ;\sigma }(\phi )|^2)\\  &   =\sum _{\varvec{j}\in \{1,2\}^{\times n}}\sum _{\varvec{m}\in (\mathbb {Z}^{2})^{\times n}}\int _{0}^{T}\textrm{d}u_1\ldots \int _{0}^{u_{n-1}}\textrm{d}u_n|\varphi ^{\otimes n}(\delta \varvec{m})|^{2}|\mathscr {F}(\sigma ^{\otimes n}\phi )(\varvec{u},-\varvec{m},\varvec{j})|^{2}\\  &   \le \Vert \varphi \Vert _{L^{\infty }}^{2n}\sum _{\varvec{j}\in \{1,2\}^{\times n}}\sum _{\varvec{m}\in (\mathbb {Z}^{2})^{\times n}}\int _{0}^{T}\textrm{d}u_1\ldots \int _{0}^{u_{n-1}}\textrm{d}u_n|\mathscr {F}(\sigma ^{\otimes n}\phi )(\varvec{u},-\varvec{m},\varvec{j})|^{2}\\  &   =\Vert \varphi \Vert _{L^{\infty }}^{2n}\sum _{\varvec{j}\in \{1,2\}^{\times n}}\int _{0}^{T}\textrm{d}u_1\ldots \int _{0}^{u_{n-1}}\textrm{d}u_n\int _{(\mathbb {T}^2)^n}\textrm{d}\varvec{x}|\sigma ^{\otimes n}(\varvec{u},\varvec{x})|^2|\phi (\varvec{u},\varvec{x},\varvec{j})|^2\\  &   \le \Vert \varphi \Vert _{L^{\infty }}^{2n}\Vert \sigma \Vert _{C_TL^\infty }^{2n}\sum _{\varvec{j}\in \{1,2\}^{\times n}}\int _{0}^{T}\textrm{d}u_1\ldots \int _{0}^{u_{n-1}}\textrm{d}u_n\int _{(\mathbb {T}^2)^n}\textrm{d}\varvec{x}|\phi (\varvec{u},\varvec{x},\varvec{j})|^2\\  &   =\Vert \varphi \Vert _{L^{\infty }}^{2n}\Vert \sigma \Vert _{C_TL^\infty }^{2n}\mathbb {E}(|I^n(\phi )|^2), \end{aligned}$$

which yields (2.8). The derivation of (2.7) for $\delta =0$ is analogous.

$$\square $$

Next we consider the second moment of the difference $I^{n}_{0;\sigma }(\phi )-I^{n}_{\delta ;\sigma }(\phi )$, where we combine the idea of Lemma 2.11 with an interpolation argument to extract a small power of $\delta$. The resulting bound (2.9) is reminiscent of [24, Lem. 8.7] in that we also need to pass to a space of lower regularity.

#### Lemma 2.12

Let $n\in \mathbb {N}$, $T>0$, $\sigma \in C_T\mathcal {H}^{1}$, $\delta \ge 0$ and $\varphi$ be as in Definition 2.1. Then for every $0<\vartheta '<\vartheta \le 1$ and $\phi \in L^2((0,T)^n_{>};\mathcal {H}^{\vartheta }(\mathbb {T}^{2n};\mathbb {C}^{2n}))$,

2.9 $$\begin{aligned}  &   \mathbb {E}(|I^{n}_{0;\sigma }(\phi )-I^{n}_{\delta ;\sigma }(\phi )|^2)\nonumber \\  &   \quad \lesssim \delta ^{2\vartheta '}\Vert 1-\varphi \Vert _{C^{1}(\mathbb {R}^{2})}^{2}(1+\Vert \varphi \Vert _{L^{\infty }}^{n-1})^{2}\Vert \sigma \Vert _{C_{T}\mathcal {H}^{1}}^{2n}\mathbb {E}(|I^{n}((1-\Delta )^{\vartheta /2}\phi )|^{2}).\nonumber \\ \end{aligned}$$

#### Proof

We use induction and interpolation to bound for every $\vartheta '\in (0,1)$,

2.10 $$\begin{aligned} |1-\varphi ^{\otimes n}(\delta \varvec{m})|\le \delta ^{\vartheta '}\Vert 1-\varphi \Vert _{C^{1}(\mathbb {R}^{2})}(1+\Vert \varphi \Vert _{L^{\infty }}^{n-1})n(1+|\varvec{m}|^{2})^{\vartheta '/2}, \end{aligned}$$

which, together with Itô’s isometry and the definition of $I^{n}_{0;\sigma }(\phi )-I^{n}_{\delta ;\sigma }(\phi )$, yields

$$\begin{aligned} \begin{aligned}&\mathbb {E}(|I^{n}_{0;\sigma }(\phi )-I^{n}_{\delta ;\sigma }(\phi )|^2)\\&\lesssim \delta ^{2\vartheta '}\Vert 1-\varphi \Vert _{C^{1}(\mathbb {R}^{2})}^{2}(1+\Vert \varphi \Vert _{L^{\infty }}^{n-1})^{2}\\&\quad \times \sum _{\varvec{j}\in \{1,2\}^{\times n}}\sum _{\varvec{m}\in (\mathbb {Z}^{2})^{\times n}}\int _{0}^{T}\textrm{d}u_1\ldots \int _{0}^{u_{n-1}}\textrm{d}u_n(1+|2\pi \varvec{m}|^{2})^{\vartheta '}|\mathscr {F}(\sigma ^{\otimes n}\phi )(\varvec{u},-\varvec{m},\varvec{j})|^{2}. \end{aligned} \end{aligned}$$

Let $0<\vartheta '<\vartheta \le 1$, to control the integrand, we use standard product estimates in Bessel potential spaces to obtain

$$\begin{aligned} \begin{aligned}&\sum _{\varvec{m}\in (\mathbb {Z}^{2})^{\times n}}(1+|2\pi \varvec{m}|^{2})^{\vartheta '}|\mathscr {F}(\sigma ^{\otimes n}\phi )(\varvec{u},-\varvec{m},\varvec{j})|^{2}\\&\quad =\Vert (\sigma ^{\otimes n}\phi )(\varvec{u},\,\cdot \,,\varvec{j})\Vert _{\mathcal {H}^{\vartheta '}(\mathbb {T}^{2n})}^{2}\\&\quad \lesssim \Vert \sigma \Vert _{C_{T}\mathcal {H}^{1}(\mathbb {T}^{2})}^{2n}\Vert \phi (\varvec{u},\,\cdot \,,\varvec{j})\Vert _{\mathcal {H}^{\vartheta }(\mathbb {T}^{2n})}^{2}. \end{aligned} \end{aligned}$$

Therefore,

$$\begin{aligned}  &   \mathbb {E}(|I^{n}_{0;\sigma }(\phi )-I^{n}_{\delta ;\sigma }(\phi )|^2)\\  &   \quad \lesssim \delta ^{2\vartheta '}\Vert 1-\varphi \Vert _{C^{1}(\mathbb {R}^{2})}^{2}(1+\Vert \varphi \Vert _{L^{\infty }}^{n-1})^{2}\Vert \sigma \Vert _{C_{T}\mathcal {H}^{1}}^{2n}\mathbb {E}(|I^{n}((1-\Delta )^{\vartheta /2}\phi )|^{2}), \end{aligned}$$

which yields (2.9). $$\square $$

#### Remark 2.13

In (2.9), to extract an exponent $\vartheta '$ of $\delta ^{2}$, we had to assume that $\sigma \in C_{T}\mathcal {H}^{1}$ and $\phi \in L^2((0,T)^n_{>};\mathcal {H}^{\vartheta }(\mathbb {T}^{2n};\mathbb {C}^{2n}))$ for some $0<\vartheta '<\vartheta \le 1$; in other words, we had to assume that $\sigma$ is regular and also incur a small loss in the regularity of $\phi$. Both assumptions can be lifted if one considers iterated Itô integrals based on the noise $\psi _{\delta }*(\sigma \varvec{\xi })$ rather than $\sigma (\psi _{\delta }*\varvec{\xi })$, where one can use that $\psi _{\delta }*(\sigma \varvec{\xi })$ may be interpreted as a mollified, vector-valued space-time white noise on an $L^{2}$-space with measure $\sigma (u,x)^{2}\textrm{d}u\textrm{d}x$.

The following result, Nelson’s estimate, allows us to bound $p\text {th}$-moments of iterated Itô integrals by their second moments.

#### Lemma 2.14

(Nelson’s estimate) Let $n\in \mathbb {N}$ and $p\in [2,\infty )$. Then, there exists a $C>0$ such that for any $\phi \in L^2((0,\infty )^{n}_{>}\times \mathbb {T}^{2n}\times \{1,2\}^{n};\mathbb {C})$,

$$\begin{aligned} \mathbb {E}(|I^n(\phi )|^p)^{1/p}\le C \mathbb {E}(|I^n(\phi )|^2)^{1/2}. \end{aligned}$$

#### Proof

For a proof, see [44, 45].

$$\square $$

The following Kolmogorov criterion provides an efficient method for establishing the regularity of stochastic processes in Hölder–Besov spaces. The presentation of this lemma is reminiscent of [49, Prop. 4.1].

#### Lemma 2.15

Let $X:[0,T]\rightarrow \mathcal {S}'(\mathbb {T}^{2})$ be a stochastic process such that $X(0)=0$ and let $\alpha \in \mathbb {R}$, $p\in (1,\infty )$, $\gamma \in (1/p,1]$. Let $\gamma '\in (0,\gamma -1/p)$ and assume there exists some $K>0$ such that

2.11 $$\begin{aligned}  &   \int _{0}^{T}\int _{0}^{T}|t-s|^{-2-p\gamma '}\sum _{q\in \mathbb {N}_{-1}}2^{pq\alpha }\nonumber \\  &   \qquad \int _{\mathbb {T}^{2}}\mathbb {E}(|\Delta _qX(t,x)-\Delta _qX(s,x)|^p)\textrm{d}x\textrm{d}s\textrm{d}t\le K<\infty . \end{aligned}$$

Then there exists a modification of *X* (which we do not relabel) such that

$$\begin{aligned} \mathbb {E}\Big (\Vert X\Vert ^p_{C^{\gamma '}_{T}\mathcal {C}^{\alpha -2/p}}\Big )\lesssim \mathbb {E}\Big (\Vert X\Vert ^p_{C^{\gamma '}_{T}\mathcal {B}_{p,p}^{\alpha }}\Big )\lesssim _{p,\gamma '}K \end{aligned}$$

and in particular $X \in C^{\gamma '}_T \mathcal {C}^{\alpha -2/p}$, $\mathbb {P}$-a.s. Assume that

$$\begin{aligned} M{:}{=}\sup _{s\ne t\in [0,T]}|t-s|^{-p\gamma }\sum _{q\in \mathbb {N}_{-1}}2^{pq\alpha }\int _{\mathbb {T}^{2}}\mathbb {E}(|\Delta _qX(t,x)-\Delta _qX(s,x)|^p)\textrm{d}x<\infty , \end{aligned}$$

then for each $\gamma '\in (0,\gamma -1/p)$, (2.11) is satisfied with

$$\begin{aligned} K=M\int _{0}^{T}\int _{0}^{T}|t-s|^{-2+p(\gamma -\gamma ')}\textrm{d}s\textrm{d}t<\infty . \end{aligned}$$

#### Proof

The bound follows by the definition of $\mathcal {B}_{p,p}^{\alpha }$ (Definition A.1), the Besov-Hölder embedding in time [20, Proof of Thm. A.10] and the Besov embedding in space (A.1). To show $X\in C^{\gamma '}_T \mathcal {C}^{\alpha -2/p}$, it suffices to exhibit a smooth, approximating sequence. This can be achieved by combining the Besov embedding (A.1) with the fact that the smooth functions are dense in $\mathcal {B}^\alpha _{p,p}$.

$$\square $$

### Diagrams of order 2 and 3

In this section we construct the second-order diagrams ,  and  and the third-order diagrams  and .

#### Lemma 2.16

Let $T>0$, $\alpha <-2$, $\kappa \in (0,1/2)$ and $\sigma \in C_{T}L^{\infty }$. Then, for any $p\in [1,\infty )$ we have  and in particular  a.s.

From now on we denote  to emphasize the separate rôles of colour and shape.

We first derive a useful upper bound on the second moments of  in terms of an explicit, time-dependent function . We call this function the *shape coefficient*.

#### Definition 2.17

Let $s,t\ge 0$ and $\omega _1,\omega _2\in 2\pi \mathbb {Z}^2\setminus \{0\}$. We define the *shape coefficient*2.12and the *increment shape coefficient*2.13

Here, the letter $\textsf{S}$ stands for shape and $\textsf{D}$ for difference. It will be clear from the proof of Lemma 2.18 that .

Shape coefficients play a central rôle in our bounds, as they capture the iterated applications of $\nabla \cdot \mathcal {I}$; they fundamentally depend on the shape of the diagram, as opposed to the additional colouring induced by $\nabla \Phi$. Using this notation, we obtain the following bound.

#### Lemma 2.18

Let $s,t\in [0,T]$, $x\in \mathbb {T}^{2}$ and $q\in \mathbb {N}_{-1}$. It holds that2.14

We refer to the prefactor $|\omega _1|^{2}|\omega _2|^{-2}|\langle \omega _2,\omega _1+\omega _2\rangle |^2$ as the *colouring* of .

Before we give the proof of Lemma 2.18, let us comment on its general strategy. In ,  and , it does not suffice to apply the triangle inequality to push the absolute value past the integral sign. This is related to the appearance of the sub-diagram , which we do not expect to be pointwise evaluable. Instead, we rely on bilinearity and expand the integrand

$$\begin{aligned} (f(t)-f(s))\overline{(g(t)-g(s))}=f(t)\overline{g(t)}+f(s)\overline{g(s)}-f(s)\overline{g(t)}-f(t)\overline{g(s)}, \end{aligned}$$

which leads to the common equation for this type of shape coefficient,

$$\begin{aligned} \textsf{D}_{s,t}=\textsf{S}_{t,t}+\textsf{S}_{s,s}-\textsf{S}_{s,t}-\textsf{S}_{t,s}. \end{aligned}$$

We refer to Lemmas 2.22, 2.24, 2.25 and 2.26 for instances where we can simplify our calculations by applying the triangle inequality.

#### Proof of Lemma 2.18

Let $s,t\in [0,T]$, $x\in \mathbb {T}^{2}$ and $q\in \mathbb {N}_{-1}$. An application of (2.7) yieldswhere  is defined byUsing that  if $\omega \ne \omega '\in \mathbb {Z}^{2}$, we obtainIt follows by an application of Itô’s isometry and Jensen’s inequality,where we used that the complex absolute value of $z\in \mathbb {C}$ is given by $|z|^{2}=z\overline{z}$.

Recalling the definition of  from (2.13), we obtainThis yields the claim. $\square$

To evaluate the integrals in , we use a case distinction over $(\omega _1\perp \omega _2)$ and $\lnot (\omega _1\perp \omega _2)$. We can then find explicit expressions for  via (2.13), which can be used to derive bounds. This is the content of Lemma B.1. We can now give the proof of Lemma 2.16.

#### Proof of Lemma 2.16

Let $T>0$ and $\gamma \in [0,1]$. To decompose the right hand side of (2.14), we introduce the orthogonal sumand the non-orthogonal sumWe obtain the decompositionwhere we used that . In the orthogonal sum $\varvec{\textrm{E}}^{\perp }$, we obtain by Lemma B.1,so thatwhere we used the orthogonality $(\omega _1\perp \omega _2)$ to identify $|\langle \omega _2,\omega _1+\omega _2\rangle |^2=|\omega _2|^{4}$. Using the orthogonality again, we obtain the bound $|\omega _1|^{2}\le |\omega _1|^{2}+|\omega _2|^{2}=|\omega |^{2}$. By applying (C.1) to the finite sum over $\omega _1\in \mathbb {Z}^{2}\setminus \{0\}$, $|\omega _1|\le |\omega |$, we arrive atNext we consider the non-orthogonal sum $\varvec{\textrm{E}}^{\lnot }$. Lemma B.1 yieldsso that by Lemma C.3 for any $\gamma \in (0,1)$ and $\varepsilon \in (0,(2-2\gamma \wedge 1))$,where we used the Cauchy–Schwarz inequality to control $|\langle \omega _2,\omega _1+\omega _2\rangle |^2\lesssim |\omega _2|^{2}|\omega _1+\omega _2|^{2}$. Applying these results to the bound (2.14), we arrive atwhich is uniform in $x\in \mathbb {T}^{2}$. Assume in addition $\varepsilon <\gamma /2$. Using that  denotes an iterated Itô integral, we obtain by Lemmas 2.14 and 2.15 for any $p\in [1,\infty )$,and therefore  a.s. for any $\kappa \in (0,1/2)$. $\square$

Next we consider the third-order diagrams  and .

#### Lemma 2.19

Let $T>0$, $\alpha <-2$, $\kappa \in (0,1/2)$ and $\sigma \in C_{T}L^{\infty }$. Then, for any $p\in [1,\infty )$ we haveand in particular  a.s.

#### Proof

The proof of this lemma is similar to the one for Lemma 2.16, so we only provide a sketch. The key idea is to consider the shape coefficientwhere the factor  was already defined in (2.12), and  is given byThe factorization  follows since there is no arrow pointing at the common root between the vertices labelled by $u_3$ and $u_4$.

We can then find explicit expressions for , which we use to bound the second moments of  and . The claim then follows by (2.7), Lemmas 2.14 and 2.15. $\square$

We can also show the existence of the diagrams  and .

#### Lemma 2.20

Let $T>0$, $\alpha <-2$, $\kappa \in (0,1/2)$ and $\sigma \in C_{T}L^{\infty }$. Then, for any $p\in [1,\infty )$ we haveand in particular  a.s.

We define a shape coefficient for  and . Since those do not contain the problematic sub-diagram , it suffices to push the absolute value past the integral sign. We denote this fact by the letter $\textsf{A}$ for absolute value. In particular, we may bound any integral over $[0,\infty )$ by $(-\infty ,\infty )$, which simplifies our calculations.

#### Definition 2.21

Let $s,t\ge 0$, $\omega _1,\omega _1',\omega _2\in \mathbb {Z}^{2}\setminus \{0\}$ and $k,k'=1,2$. We set

We can then bound the second moment of  in terms of this object.

#### Lemma 2.22

Let $s,t\in [0,T]$, $x\in \mathbb {T}^{2}$, $k,j=1,2$ and $q\in \mathbb {N}_{-1}$. It holds that

#### Proof of Lemma 2.20

We provide a sketch. The shape coefficient is controlled in Lemma B.3, we can then use fairly direct estimates and apply Lemmas 2.14 and 2.15 as before. We observe that  differs from  only in its colouring of the $\omega _1$ and $\omega _2$ arrows, with the sum of the exponents preserved. Consequently by the same arguments as for , we can also construct . $\square$

### Wick contractions

In this section we construct the contractions , ,  and .

The diagrams ,  differ from ,  and , , despite their similarity in structure. The first two are well-defined, since two applications of $\nabla \Phi$ appear inside the -shaped sub-diagram. This is not the case for ,  and ,  as one may tell by the distribution of highlighted arrows.

However, by adding the problematic diagrams,  and  we can make use of the symmetry $G^{j}(\omega _{4})=-G^{j}(-\omega _{4})$, for all $j=1,2$ and $\omega _{4}\in \mathbb {Z}^{2}$, to establish the existence of the summed objects.

We define a shape coefficient for those diagrams.

#### Definition 2.23

Let $s,t\ge 0$, $\omega _1,\omega _2,\omega _3\in \mathbb {Z}^{2}\setminus \{0\}$ and $k=1,2$. We set

In Lemma 2.24 we establish the existence of , ,  and in Lemma 2.25, we establish the existence of .

#### Lemma 2.24

Let $T>0$, $\alpha <-2$, $\kappa \in (0,1/2)$ and $\sigma \in C_{T}\mathcal {H}^{2}$. Then, for any $p\in [1,\infty )$ we haveand in particular  a.s.

#### Lemma 2.25

Let $T>0$, $\alpha <-2$, $\kappa \in (0,1/2)$ and $\sigma \in C_{T}\mathcal {H}^{2}$. Then, it holds that

We first show Lemma 2.24. Let us focus on , the derivation for  and  is similar, but easier.

#### Proof of Lemma 2.24

Let $s,t\in [0,T]$, $x\in \mathbb {T}^{2}$, $j=1,2$ and $q\in \mathbb {N}_{-1}$. An application of (2.7) yieldswhere  is defined byUsing the definition of the Littlewood–Paley block $\Delta _{q}$, uniformly in $x\in \mathbb {T}^{2}$,We apply Itô’s isometry and a decay estimate (Lemma C.1) for the symmetrized elliptic multiplier $G^{j}(\omega _{1}+\omega _{2})+G^{j}(\omega _{4})=G^{j}(\omega -\omega _{4})-G^{j}(-\omega _{4})$. We obtain the bound2.15We control the shape coefficient with Lemma B.2 and obtain for all $\gamma \in [0,1]$,We can then plug this expression into (2.15) and apply Lemma C.6 to control the sums over $\omega _4$ and $m_2\in \mathbb {Z}^{2}$. Let $\gamma \in (0,1/2)$ and $\varepsilon \in (0,1-2\gamma )$, it follows that2.16Hence it suffices to control the remaining sum over $\omega _1\in \mathbb {Z}^{2}\setminus \{0\}$,

$$\begin{aligned} \sum _{\omega _1\in \mathbb {Z}^{2}\setminus \{0\}}|\omega _1|^{-2}(1\vee |\omega -\omega _1|)^{-2+\varepsilon }(1\vee |\omega '-\omega _1|)^{-2+\varepsilon }. \end{aligned}$$

We distinguish the cases $\omega =\omega '$ and $\omega \ne \omega '$. In the case $\omega =\omega '$, we decompose the sum into the regions $\omega _1=\omega$ and $\omega _1\in \mathbb {Z}^{2}\setminus \{0,\omega \}$. We then estimate by Lemma C.3,

$$\begin{aligned}  &   \sum _{\omega _1\in \mathbb {Z}^{2}\setminus \{0\}}|\omega _1|^{-2}(1\vee |\omega -\omega _1|)^{-4+2\varepsilon }\\  &   \quad =|\omega |^{-2}+\sum _{\omega _1\in \mathbb {Z}^{2}\setminus \{0,\omega \}}|\omega _1|^{-2}|\omega -\omega _1|^{-4+2\varepsilon }\lesssim |\omega |^{-2+2\varepsilon }. \end{aligned}$$

In the case $\omega \ne \omega '$, we decompose the sum into the regions $\omega _1=\omega$, $\omega _1=\omega '$ and $\omega _1\in \mathbb {Z}^{2}\setminus \{0,\omega ,\omega '\}$,

$$\begin{aligned} \begin{aligned}&\sum _{\omega _1\in \mathbb {Z}^{2}\setminus \{0\}}|\omega _1|^{-2}(1\vee |\omega -\omega _1|)^{-2+\varepsilon }(1\vee |\omega '-\omega _1|)^{-2+\varepsilon }\\&=(|\omega |^{-2}+|\omega '|^{-2})|\omega -\omega '|^{-2+\varepsilon }+\sum _{\omega _1\in \mathbb {Z}^{2}\setminus \{0,\omega ,\omega '\}}|\omega _1|^{-2}|\omega -\omega _1|^{-2+\varepsilon }|\omega '-\omega _1|^{-2+\varepsilon } \end{aligned} \end{aligned}$$

and apply Lemma C.4 to bound

$$\begin{aligned}  &   \sum _{\omega _1\in \mathbb {Z}^{2}\setminus \{0,\omega ,\omega '\}}|\omega _1|^{-2}|\omega -\omega _1|^{-2+\varepsilon }|\omega '-\omega _1|^{-2+\varepsilon }\\  &   \quad \lesssim |\omega -\omega '|^{-2+\varepsilon }|\omega |^{-2+2\varepsilon }+|\omega -\omega '|^{-2+\varepsilon }|\omega '|^{-2+2\varepsilon }. \end{aligned}$$

Assume $q\in \mathbb {N}_{0}$ and $\omega ,\omega '\in \text {supp}(\varrho _q)$. It follows that $2^q\lesssim |\omega |,|\omega '|\lesssim 2^q$ and if $\omega \ne \omega '$ then $2^q\lesssim |\omega -\omega '|\lesssim 2^q$ as well. We obtain by (2.16),Assume in addition $\varepsilon <\gamma$. We obtain by Lemmas 2.14 and 2.15 for any $p\in [1,\infty )$,and therefore  a.s. for any $\kappa \in (0,1/2)$. $\square$

Next we prove the existence of .

#### Proof of Lemma 2.25

Let $0\le s\le t\le T$, $\omega \in \mathbb {Z}^2$ and $k,j=1,2$. We can bound the incrementWe control the shape coefficient with Lemma B.2 and the elliptic multiplier with Lemma C.1. Let $\kappa \in [0,1]$, we arrive atLet $\varepsilon \in (0,1)$. We bound by Lemma C.3,

$$\begin{aligned} \begin{aligned}&\sum _{m_2\in \mathbb {Z}^{2}}(1+|\omega _1+m_2|^{2})^{-1}(1+|\omega _2-m_2|^{2})^{-1}\\&=2(1+|\omega |^{2})^{-1}+\sum _{m_2\in \mathbb {Z}^{2}\setminus \{-\omega _1,\omega _2\}}(1+|\omega _1+m_2|^{2})^{-1}(1+|\omega _2-m_2|^{2})^{-1}\\&\lesssim |\omega |^{-2+2\varepsilon } \end{aligned} \end{aligned}$$

and obtainFor any $\kappa \in (0,1/2)$, we bound by Lemma C.3,It follows directly thatand we obtain  for any $\kappa \in (0,1/2)$.

$$\square $$

### Construction of the canonical enhancement

In this section we construct  and ,  for correlation lengths $\delta >0$. Additionally, we bound the speed of divergence as $\delta \rightarrow 0$ by a logarithmic rate using the symmetry of the elliptic equation.

#### Lemma 2.26

Let $T>0$, $\alpha <-2$, $\kappa \in (0,1)$, $\delta >0$, $\sigma \in C_{T}\mathcal {H}^{2}$ and $\varphi$ be as in Definition 2.1. Then, it holds thatand for any $\kappa \in (0,1/2)$, $p\in [1,\infty )$,and

#### Proof

We first establish . Let $s,t\in [0,T]$, $\omega \in \mathbb {Z}^{2}$, $\kappa \in (0,1)$ and $\varepsilon \in (0,1/2)$. It suffices to consider $\omega \in \mathbb {Z}^{2}\setminus \{0\}$, since . We symmetrize the contraction. Changing the rôles of $\omega _1$, $\omega _2$ in the definition of  and using that $\varphi$ is even, we obtainWe apply the triangle inequality, Lemma C.1 and (A.2) to boundWe can now apply Lemma C.7 to control the sums over $m_1\in \mathbb {Z}^{2}$, $|m_1|\le \delta ^{-1}$, and $\omega _1\in \mathbb {Z}^{2}\setminus \{0,\omega \}$,and soThe proof that  with a divergence of order $1\vee (\delta ^{-1}\log (\delta ^{-1}))$ follows by a similar derivation, where we skip the symmetrization and use that $|\varphi (\delta m_1)|\lesssim (1+|\delta m_1|^{2})^{-1/4}\Vert \varphi \Vert _{L^{\infty }}$.

Next we establish that . We first consider . We apply Itô’s isometry, (2.8) and Lemma C.1 to bound uniformly in $x\in \mathbb {T}^{2}$,Assume $s\le t$ and $\gamma \in [0,1]$. We apply Lemma B.3 to control the shape coefficient. We can then apply Lemma C.7 and obtain for $\varepsilon \in (0,1/2)$, $\delta >0$,Assume $6\varepsilon <4-2\gamma$. We apply Hölder’s inequality,

2.17 $$\begin{aligned} \begin{aligned}&\sum _{\begin{array}{c} \omega _4\in \mathbb {Z}^{2}\setminus \{0,\omega ,\omega '\}\\ (\omega -\omega _4)\sim \omega _4\\ (\omega '-\omega _4)\sim \omega _4 \end{array}}|\omega _4|^{-2}|\omega -\omega _4|^{-2+\gamma +3\varepsilon }|\omega '-\omega _4|^{-2+\gamma +3\varepsilon }\\&\le \Big (\sum _{\begin{array}{c} \omega _4\in \mathbb {Z}^{2}\setminus \{0,\omega \}\\ (\omega -\omega _4)\sim \omega _4 \end{array}}|\omega _4|^{-2}|\omega -\omega _4|^{-4+2\gamma +6\varepsilon }\Big )^{1/2}\Big (\sum _{\begin{array}{c} \omega _4\in \mathbb {Z}^{2}\setminus \{0,\omega '\}\\ (\omega '-\omega _4)\sim \omega _4 \end{array}}|\omega _4|^{-2}|\omega '-\omega _4|^{-4+2\gamma +6\varepsilon }\Big )^{1/2}\\&\lesssim (1\vee |\omega |)^{-2+\gamma +3\varepsilon }(1\vee |\omega '|)^{-2+\gamma +3\varepsilon }, \end{aligned}\nonumber \\ \end{aligned}$$

which impliesAssume in addition $\gamma \in (0,1)$ and $\varepsilon \in (0,\gamma /2)$, we obtain by Lemmas 2.14 and 2.15 for any $p\in [1,\infty )$,and therefore  a.s. for any $\kappa \in (0,1/2)$.

The only difference between  and  is that the factor $G^{j}(\omega _1+\omega _2)$ replaces $G^{j}(\omega _4)$. Instead of (2.17), we estimate by Hölder’s inequality,

$$\begin{aligned}  &   \sum _{\begin{array}{c} \omega _4\in \mathbb {Z}^{2}\setminus \{0,\omega ,\omega '\}\\ (\omega -\omega _4)\sim \omega _4\\ (\omega '-\omega _4)\sim \omega _4 \end{array}}|\omega -\omega _4|^{-3+\gamma +3\varepsilon }|\omega '-\omega _4|^{-3+\gamma +3\varepsilon }\\  &   \quad \le \Big (\sum _{\begin{array}{c} \omega _4\in \mathbb {Z}^{2}\setminus \{\omega \}\\ (\omega -\omega _4)\sim \omega _4 \end{array}}|\omega -\omega _4|^{-6+2\gamma +6\varepsilon }\Big )^{1/2}\Big (\sum _{\begin{array}{c} \omega _4\in \mathbb {Z}^{2}\setminus \{\omega '\}\\ (\omega '-\omega _4)\sim \omega _4 \end{array}}|\omega '-\omega _4|^{-6+2\gamma +6\varepsilon }\Big )^{1/2}\\  &   \quad \lesssim (1\vee |\omega |)^{-2+\gamma +3\varepsilon }(1\vee |\omega '|)^{-2+\gamma +3\varepsilon }. \end{aligned}$$

As before, we obtain by (2.8), Lemmas 2.14 and 2.15 for any $\gamma \in (0,1)$, $6\varepsilon <4-2\gamma$, $\varepsilon <\gamma /2$, $\delta >0$ and $p\in [1,\infty )$,and therefore  a.s. for any $\kappa \in (0,1/2)$. $\square$

#### Remark 2.27

We may also construct , which is similar to  but without the lower stem (cf. (2.5)). However, to obtain $\kappa$-time regularity, we need to trade $2\kappa$-space regularity in the parabolic multipliers $H_{t-u_1}^{j_1}(\omega _1)H_{t-u_1}^{j_1}(\omega _2)$. We then sum over $\omega _1,\omega _2\in \mathbb {Z}^{2}$, hence we will be stuck with a divergence of $\delta ^{-2\kappa }$, where $\kappa$ is arbitrarily small but positive.

#### Remark 2.28

We can show that , if $\sigma \equiv 1$. Indeed, if we choose $\sigma \equiv 1$, then ${\widehat{\sigma }}(u,\omega )=\mathbb {1}_{\omega =0}$. Consequently on the right hand side of (2.5), $\omega _1=m_1$ and $\omega _2=-m_1$. By the symmetrization, we obtain the factor $G^{j}(\omega _1)+G^{j}(\omega _2)$. Using that $G^{j}$ is odd and that $\omega _1=-\omega _2$, we see that this term is zero, so that . Similarly, , if $\sigma \equiv 1$.

## Existence of paracontrolled solutions

In this section we show the existence and uniqueness of a paracontrolled solution to (1.1) given an abstract enhancement, where the notion of paracontrolled solution has previously been motivated in (1.11)–(1.12) and will be made precise in Definition 3.10.

In Sect. 3.1 we consider the solution on small time intervals (Lemma 3.12), which we then extend in Sect. 3.2 to a maximal time of existence (Lemma 3.20). In Sect. 3.3 we then combine the deterministic solution theory above with the stochastic existence of the renormalised enhancement (Theorem 2.3) to construct the renormalised solution to (1.1) as a random variable (Theorem 3.22, Part 1). We can then show that solutions to (1.13) converge in probability to the renormalised solution (Theorem 3.22, Part 2).

### Local well-posedness

Throughout we fix exponents satisfying the assumptions below. To explain their usage: *p* and $\beta _0$ will be the integrabillity and regularity exponents of the admissible initial condition in the Besov scale $\mathcal {B}^{\beta _0}_{p,q}$, where *q* is the microscopic parameter; $\alpha$ will be the regularity of the space-time white noise, so that almost surely ; $\beta$ measures the regularity of the second Da Prato–Debussche remainder, *w*, in the Hölder scale and $\eta$ the allowed blow-up of *w* at $t=0$; $\beta '$ measures the regularity of the Gubinelli derivative $w'$; $\beta ^{\#}$ measures the maximal spatial regularity of the paracontrolled remainder and finally $\kappa$ will be used to denote time regularity.

From now on we fix $(\alpha ,q,p,\beta ,\beta ',\beta ^{\#},\beta _{0},\kappa ,\eta )$ satisfying

3.1 $$\begin{aligned} \begin{aligned} \alpha \in (-9/4,-2),&\qquad q\in [1,\infty ],\\ p\in (2/(\alpha +3),\infty ],&\qquad \beta \in (-1/2,\alpha +2),\\ \beta '\in (-2\alpha -4,(\beta +1)\wedge (2\alpha +5)],&\qquad \beta ^{\#}\in (-\alpha -2,\alpha +\beta '+2),\\ \beta _{0}\in (\beta +2/p-(\alpha +3),\beta ^{\#}],&\qquad \kappa \in ((\beta ^{\#}-\alpha -2)/2,1/2),\\ \eta \in \Big [\Big (\Big (\frac{\beta -\beta _{0}}{2}\vee 0\Big )+\frac{1}{p}\Big )&\vee \Big (\frac{\beta ^{\#}-\beta _0}{4}+\frac{1}{2p}\Big ),\frac{\alpha +3}{2}\Big ). \end{aligned}\nonumber \\ \end{aligned}$$

One can confirm that the intervals in (3.1) are non-empty. Particular choices of exponents allow us to consider regular and irregular initial data.

#### Example 3.1

By taking $p=\infty$, $\beta _{0}=\beta ^{\#}$ and $\eta =0$, we can choose as initial data any $\rho _{0}\in \mathcal {B}_{\infty ,q}^{\beta ^{\#}}$, without incurring a blow-up at 0.

#### Example 3.2

By taking $p=q=\infty$ and $\beta <2\alpha +4$, we can choose as initial data any $\rho _{0}\in \mathcal {C}^{\alpha +1}$, which is the natural regularity of  and the state space of .

#### Example 3.3

Using that $L^{p}\hookrightarrow \mathcal {B}_{p,\infty }^{0}$ (see Appendix 1), we can choose as initial data any $\rho _{0}\in L^{p}$ with $p>2$.

In what follows, we fix an arbitrary tuple of exponents $(\alpha ,q,p,\beta ,\beta ',\beta ^{\#},\beta _{0},\kappa ,\eta )$ satisfying (3.1).

Let us fix a $T>0$. We define the space of paracontrolled distributions.

#### Definition 3.4

Let $\mathbb {X}\in \mathcal {X}^{\alpha ,\kappa }_{T}$ and $\rho _0\in \mathcal {B}_{p,q}^{\beta _0}$. We define the space

$$\begin{aligned} \mathscr {D}_{T}\subset \mathscr {L}_{\eta ;T}^{\kappa }\mathcal {C}^{\beta }(\mathbb {T}^2;\mathbb {R})\times \mathscr {L}_{\eta ;T}^{\kappa }\mathcal {C}^{\beta '}(\mathbb {T}^2;\mathbb {R}^{2})\times (\mathscr {L}_{\eta ;T}^{\kappa }\mathcal {C}^{\beta }(\mathbb {T}^2;\mathbb {R})\cap \mathscr {L}_{2\eta ;T}^{\kappa }\mathcal {C}^{\beta ^{\#}}(\mathbb {T}^2;\mathbb {R})) \end{aligned}$$

of distributions paracontrolled by $\mathbb {X}$ as those triples $\varvec{w}{:}{=}(w,w', w^\#)$, such that3.2as well as $\lim _{t\rightarrow 0}w_{t}=\lim _{t\rightarrow 0}w_{t}^{\#}=\rho _{0}$ in $\mathcal {S}'(\mathbb {T}^{2};\mathbb {R})$ and $\lim _{t\rightarrow 0}w'_{t}=\nabla \Phi _{\rho _{0}}$ in $\mathcal {S}'(\mathbb {T}^{2};\mathbb {R}^{2})$. We equip this space with the metric induced by the norm

$$\begin{aligned} \Vert \varvec{w}\Vert _{\mathscr {D}_{T}}{:}{=}\max \{\Vert w\Vert _{\mathscr {L}_{\eta ;T}^{\kappa }\mathcal {C}^{\beta }},\Vert w'\Vert _{\mathscr {L}_{\eta ;T}^{\kappa }\mathcal {C}^{\beta '}},\Vert w^{\#}\Vert _{\mathscr {L}_{\eta ;T}^{\kappa }\mathcal {C}^{\beta }},\Vert w^{\#}\Vert _{\mathscr {L}_{2\eta ;T}^{\kappa }\mathcal {C}^{\beta ^{\#}}}\}. \end{aligned}$$

#### Remark 3.5

The Ansatz (3.2) allows us to write the mild equation for $w^{\#}$ as $w^{\#}=P\rho _{0}+\nabla \cdot \mathcal {I}[\Omega ^{\#}(\varvec{w})]$, with some $\Omega ^{\#}(\varvec{w})$ determined by (1.9). We can hence simplify our estimates by using the space-time regularization of $\mathcal {I}$. Note that (3.2) is equivalent to the Ansatz (1.10) discussed in the introduction, up to commutators.

#### Lemma 3.6

Given $\mathbb {X}\in \mathcal {X}^{\alpha ,\kappa }_{T}$ and $\rho _0\in \mathcal {B}_{p,q}^{\beta _0}$, the space $\mathscr {D}_T$ is a non-empty, complete metric space.

#### Proof

To show that $\mathscr {D}_T$ is non-empty, we can choose $w'=\nabla \Phi _{P\rho _0}$, $w^\#=P\rho _0$ and set . The initial condition is satisfied, since $\lim _{t\rightarrow 0}w'_{t}=\nabla \Phi _{\rho _0}$ in $\mathcal {S}'(\mathbb {T}^{2};\mathbb {R}^{2})$ and $\lim _{t\rightarrow 0}w^{\#}_{t}=\rho _{0}$ in $\mathcal {S}'(\mathbb {T}^{2};\mathbb {R})$. By Lemmas A.6 and A.8, using that $\beta '\le \beta +1$, and $\big ((\beta -\beta _{0})/2\vee 0\big )+1/p\le \eta$, we obtain $w'=\nabla \Phi _{P\rho _0}\in \mathscr {L}_{\eta ;T}^{\kappa }\mathcal {C}^{\beta '}$, $w^{\#}=P\rho _0\in \mathscr {L}_{\eta ;T}^{\kappa }\mathcal {C}^{\beta }$ and

$$\begin{aligned} \Vert \nabla \Phi _{P\rho _0}\Vert _{\mathscr {L}^{\kappa }_{\eta ;T}\mathcal {C}^{\beta '}}\lesssim \Vert \nabla \Phi _{P\rho _0}\Vert _{\mathscr {L}^{\kappa }_{\eta ;T}\mathcal {C}^{\beta +1}}\lesssim \Vert P\rho _0\Vert _{\mathscr {L}^{\kappa }_{\eta ;T}\mathcal {C}^{\beta }}\lesssim _{T}\Vert \rho _0\Vert _{\mathcal {B}_{p,q}^{\beta _{0}}}. \end{aligned}$$

Using that $\beta _{0}\le \beta ^{\#}$ and $(\beta ^{\#}-\beta _{0})/2+1/p\le 2\eta$, we obtain $w^{\#}\in \mathscr {L}_{2\eta ;T}^{\kappa }\mathcal {C}^{\beta ^{\#}}$ and

$$\begin{aligned} \Vert P\rho _0\Vert _{\mathscr {L}^{\kappa }_{2\eta ;T}\mathcal {C}^{\beta ^{\#}}}\lesssim _{T}\Vert \rho _0\Vert _{\mathcal {B}_{p,q}^{\beta _{0}}}. \end{aligned}$$

Since , we find by an application of the triangle inequality, Lemmas A.6 and A.4, that $w\in \mathscr {L}_{\eta ;T}^{\kappa }\mathcal {C}^{\beta }$ andwhere we used that $\beta <\alpha +2$ and $\beta '>0$.

To show completeness, let $(\varvec{w}_n)_{n\in \mathbb {N}}$ be a Cauchy sequence in $\mathscr {D}_{T}$. By the completeness of $\mathscr {L}_{\eta ;T}^{\kappa }\mathcal {C}^{\beta }\times \mathscr {L}_{\eta ;T}^{\kappa }\mathcal {C}^{\beta '}\times (\mathscr {L}_{\eta ;T}^{\kappa }\mathcal {C}^{\beta }\cap \mathscr {L}_{2\eta ;T}^{\kappa }\mathcal {C}^{\beta ^{\#}})$, we obtain $w_n\rightarrow w\in \mathscr {L}_{\eta ;T}^{\kappa }\mathcal {C}^{\beta }$, $w'_{n}\rightarrow w'\in \mathscr {L}_{\eta ;T}^{\kappa }\mathcal {C}^{\beta '}$, $w^{\#}_{n}\rightarrow w^{\#}\in \mathscr {L}_{\eta ;T}^{\kappa }\mathcal {C}^{\beta }$ and $w^{\#}_{n}\rightarrow w^{\#}\in \mathscr {L}_{2\eta ;T}^{\kappa }\mathcal {C}^{\beta ^{\#}}$ all with the correct initial conditions. It suffices to show  so that $\varvec{w}\in \mathscr {D}_{T}$. Then again by Lemmas A.6 and A.4,which combined with $w_n\rightarrow w\in \mathscr {L}_{\eta ;T}^{\kappa }\mathcal {C}^{\beta }$ yields .

$$\square $$

In the next lemma we show that  is well-defined on $\mathscr {D}_{T}\times \mathcal {X}^{\alpha ,\kappa }_{T}$.

#### Lemma 3.7

There exists a continuous operator $\mathscr {P}:\mathscr {D}_T\times \mathcal {X}^{\alpha ,\kappa }_{T}\rightarrow C_{2\eta ;T}\mathcal {C}^{2\alpha +4}$, such that when all objects are smooth,

#### Proof

Using the notation  (see Lemma A.11) and recalling that  we can expand the product intowhere $\varvec{w}\in \mathscr {D}_{T}$ and $\mathbb {X}\in \mathcal {X}^{\alpha ,\kappa }_{T}$. In what follows, we establish the bound3.3by various applications of our commutator results. The regularity $\mathscr {P}(\varvec{w},\mathbb {X})\in C_{2\eta ;T}\mathcal {C}^{2\alpha +4}$ follows by the same arguments.

Using Lemma A.11 and that $\beta '\in (0,1)$, $2\alpha +4<0<2\alpha +4+\beta '$, we obtainandFurther, by Lemma A.4, using that $2\alpha +4<0<\beta ^{\#}+\alpha +2$,To control the remainder, we apply Lemmas A.9 and A.10, using that $\kappa \in ((\beta ^{\#}-\alpha -2)/2,1/2)$ and $\beta ^{\#}<\alpha +\beta '+2$,Similarly, by Lemmas A.4, A.9 and A.10, using that $2\alpha +4<0<\beta ^{\#}+\alpha +2$,Finally, by Lemma A.4, using that $2\alpha +4<0<2\alpha +4+\beta '$,This yields the claim.

$$\square $$

We can now derive a priori bounds for our solution map.

#### Lemma 3.8

Let $\mathbb {X}\in \mathcal {X}^{\alpha ,\kappa }_{T}$ and $\rho _0\in \mathcal {B}_{p,q}^{\beta _{0}}$. Let $\varvec{\Psi }$, acting on $\varvec{u}=(u,u',u^{\#})\in \mathscr {D}_T$, be given by $\varvec{\Psi }(\varvec{u}){:}{=}(w,w',w^\#)$, wherewithThen $\varvec{\Psi }(\varvec{u})\in \mathscr {D}_{T}$ and there exists some $\theta >0$ depending only on the chosen exponents and the dimension, such that for $T\le 1$,

3.4 $$\begin{aligned}&\max \{\Vert w\Vert _{\mathscr {L}_{\eta ;T}^{\kappa }\mathcal {C}^{\beta }},\Vert w^\#\Vert _{\mathscr {L}_{\eta ;T}^{\kappa }\mathcal {C}^{\beta }},\Vert w^\#\Vert _{\mathscr {L}_{2\eta ;T}^{\kappa }\mathcal {C}^{\beta ^{\#}}}\}\lesssim (1+T^{\theta }\Vert \varvec{u}\Vert _{\mathscr {D}_T})^{2}(1+\Vert \mathbb {X}\Vert _{\mathcal {X}^{\alpha ,\kappa }_{T}}+\Vert \rho _0\Vert _{\mathcal {B}_{p,q}^{\beta _0}})^{2}, \end{aligned}$$

3.5 $$\begin{aligned}&\Vert w'\Vert _{\mathscr {L}^{\kappa }_{\eta ;T}\mathcal {C}^{\beta '}}\lesssim \Vert u\Vert _{\mathscr {L}_{\eta ;T}^{\kappa }\mathcal {C}^{\beta }}+\Vert \mathbb {X}\Vert _{\mathcal {X}^{\alpha ,\kappa }_{T}}. \end{aligned}$$

#### Proof

We derive bounds for our solution map in several steps. By the same arguments it also follows that $\varvec{\Psi }(\varvec{u})\in \mathscr {D}_{T}$. In the remainder of the proof, we will assume $T\le 1$.

*Step* 1. *The* $\mathscr {L}_{\eta ;T}^{\kappa }\mathcal {C}^{\beta }$-*regularity of* *w*. As in the proof of Lemma 3.6, but this time keeping track of the dependency on *T*, we see that*Step* 2. *The* $\mathscr {L}_{\eta ;T}^{\kappa }\mathcal {C}^{\beta }$ *and* $\mathscr {L}_{2\eta ;T}^{\kappa }\mathcal {C}^{\beta ^{\#}}$-*regularity of* $w^\#$. To establish the $\mathscr {L}_{\eta ;T}^{\kappa }\mathcal {C}^{\beta }$-regularity of $w^{\#}$, we apply Lemma A.6, using that $((\beta -\beta _{0})/2\vee 0)+1/p\le \eta$, $\eta <1/2$, $\beta +1<\alpha +\beta +4$ and $-(\alpha +1)/2\vee \kappa <1-\eta$,

$$\begin{aligned} \Vert w^{\#}\Vert _{\mathscr {L}_{\eta ;T}^{\kappa }\mathcal {C}^{\beta }}\lesssim T^{\eta -\big (\frac{\beta -\beta _0}{2}\vee 0\big )-\frac{1}{p}}\Vert \rho _0\Vert _{\mathcal {B}_{p,q}^{\beta _0}}+(T^{\frac{\alpha +3}{2}-\eta }\vee T^{1-\kappa -\eta })\Vert \Omega ^{\#}(\varvec{u})\Vert _{C_{2\eta ;T}\mathcal {C}^{\alpha +\beta +2}}. \end{aligned}$$

To establish the $\mathscr {L}_{2\eta ;T}^{\kappa }\mathcal {C}^{\beta ^{\#}}$-regularity of $w^{\#}$, we apply Lemma A.6, using that $\beta _{0}\le \beta ^{\#}$, $(\beta ^{\#}-\beta _{0})/2+1/p\le 2\eta$, $\eta {<}1/2$, $\beta ^{\#}+1{<}\alpha +\beta +4$ and $(\beta ^{\#}-\alpha -\beta -1)/2\vee \kappa {<}1$,

$$\begin{aligned} \Vert w^{\#}\Vert _{\mathscr {L}_{2\eta ;T}^{\kappa }\mathcal {C}^{\beta ^{\#}}}\lesssim T^{2\eta -\frac{\beta ^{\#}-\beta _0}{2}-\frac{1}{p}}\Vert \rho _0\Vert _{\mathcal {B}_{p,q}^{\beta _0}}+(T^{1-\frac{\beta ^{\#}-\alpha -\beta -1}{2}}\vee T^{1-\kappa })\Vert \Omega ^{\#}(\varvec{u})\Vert _{C_{2\eta ;T}\mathcal {C}^{\alpha +\beta +2}}. \end{aligned}$$

*Step* 3. *The* $C_{2\eta ;T}\mathcal {C}^{\alpha +\beta +2}$-*regularity of* $\Omega ^\#(\varvec{u})$. We obtain by various applications of Lemma A.4, using in particular that $4\alpha +9>0$ and $\beta >-1/2$,andBy (3.3) of Lemma 3.7, using that $\alpha +\beta +2\le 2\alpha +4$,*Step* 4. *The* $\mathscr {L}_{\eta ;T}^{\kappa }\mathcal {C}^{\beta +1}$-*regularity of* $w'$. By definition, . Using that $\beta '\le \beta +1$ and $\beta '\le 2\alpha +5$, we obtain*Step* 5. *Closing the bounds.* Using that $T\le 1$, we can collect all of the terms above and cast them in the form (3.4)–(3.5). This yields the claim. $\square$

While Lemma 3.8 shows that $\varvec{\Psi }$ is a map from $\mathscr {D}_T$ to itself, it is not a contraction for small *T*, since there is no small time parameter on the right hand side of (3.5). The remedy is to apply $\varvec{\Psi }$ twice and argue that a fixed point of $\varvec{\Psi }^{\circ 2}$ is also a fixed point of $\varvec{\Psi }$ itself.

#### Proposition 3.9

Let $\mathbb {X}\in \mathcal {X}^{\alpha ,\kappa }_{T}$ and $\rho _0\in \mathcal {B}_{p,q}^{\beta _{0}}$. Then there exists some $\bar{T}\in (0,T\wedge 1]$ such that there is a unique solution $\varvec{w}=(w,w',w^{\#})\in \mathscr {D}_{\bar{T}}$ to the equation3.6The time of existence $\bar{T}$ depends only on $\Vert \rho \Vert _{\mathcal {B}_{p,q}^{\beta _{0}}}$, $\Vert \mathbb {X}\Vert _{\mathcal {X}^{\alpha ,\kappa }_{T}}$, the chosen exponents and the dimension.

#### Proof

We let $\bar{T}\in (0,T]$, $\bar{T}\le 1$, $\mathbb {X}\in \mathcal {X}^{\alpha ,\kappa }_{T}$, $\varvec{u}\in \mathscr {D}_{\bar{T}}$ and define

$$\begin{aligned} (\Psi (\varvec{u}),\Psi (\varvec{u})',\Psi (\varvec{u})^\#){:}{=}\varvec{\Psi }(\varvec{u}). \end{aligned}$$

By Lemma 3.8 there exists some $\theta >0$ such that

3.7 $$\begin{aligned}  &   \max \{\Vert \Psi (\varvec{u})\Vert _{\mathscr {L}_{\eta ;\bar{T}}^{\kappa }\mathcal {C}^{\beta }},\Vert \Psi (\varvec{u})^{\#}\Vert _{\mathscr {L}_{\eta ;\bar{T}}^{\kappa }\mathcal {C}^{\beta }},\Vert \Psi (\varvec{u})^{\#}\Vert _{\mathscr {L}_{2\eta ;\bar{T}}^{\kappa }\mathcal {C}^{\beta ^{\#}}}\}\nonumber \\  &   \quad \lesssim (1+\bar{T}^{\theta }\Vert \varvec{u}\Vert _{\mathscr {D}_{\bar{T}}})^{2}(1+\Vert \mathbb {X}\Vert _{\mathcal {X}^{\alpha ,\kappa }_{\bar{T}}} +\Vert \rho _0\Vert _{\mathcal {B}_{p,q}^{\beta _{0}}})^{2}, \end{aligned}$$

and

3.8 $$\begin{aligned} \Vert \Psi (\varvec{u})'\Vert _{\mathscr {L}_{\eta ;\bar{T}}^{\kappa }\mathcal {C}^{\beta '}}\lesssim \Vert u\Vert _{\mathscr {L}_{\eta ;\bar{T}}^{\kappa }\mathcal {C}^{\beta }}+\Vert \mathbb {X}\Vert _{\mathcal {X}^{\alpha ,\kappa }_{\bar{T}}}. \end{aligned}$$

Now denote

$$\begin{aligned} (\Psi ^{\circ 2}(\varvec{u}),\Psi ^{\circ 2}(\varvec{u})',\Psi ^{\circ 2}(\varvec{u})^{\#}){:}{=}\varvec{\Psi }^{\circ 2}(\varvec{u}). \end{aligned}$$

By iterating the bounds (3.7)–(3.8), using $\bar{T}\le 1$ to streamline exponents, we obtain

3.9 $$\begin{aligned} \Vert \varvec{\Psi }^{\circ 2}(\varvec{u})\Vert _{\mathscr {D}_{\bar{T}}}\lesssim (1+\bar{T}^{\theta /2}\Vert \varvec{u}\Vert _{\mathscr {D}_{\bar{T}}})^{4}(1+\Vert \mathbb {X}\Vert _{\mathcal {X}^{\alpha ,\kappa }_{\bar{T}}}+\Vert \rho _0\Vert _{\mathcal {B}_{p,q}^{\beta _0}})^{6}. \end{aligned}$$

Let $C>0$ be larger than the implicit constants of the inequalities (3.7) and (3.9) above. Assume that $M,R>0$ are sufficiently large such that

$$\begin{aligned} (1+\Vert \mathbb {X}\Vert _{\mathcal {X}^{\alpha ,\kappa }_{T}}+\Vert \rho _0\Vert _{\mathcal {B}_{p,q}^{\beta _{0}}})<M,\qquad 2CM^{6}<R. \end{aligned}$$

Assume further that $\Vert \varvec{u}\Vert _{\mathscr {D}_{\bar{T}}}<R$. Using the bound (3.9), we can choose $\bar{T}=\bar{T}(R,\theta )\le 1$ smaller, if necessary, such that

$$\begin{aligned} \Vert \varvec{\Psi }^{\circ 2}(\varvec{u})\Vert _{\mathscr {D}_{\bar{T}}}\le C(1+\bar{T}^{\theta /2}R)^{4}M^{6}\le 2CM^{6}<R. \end{aligned}$$

Consequently, $\varvec{\Psi }^{\circ 2}$ is a self-mapping on the ball

$$\begin{aligned} \mathfrak {B}_{R;\bar{T}}{:}{=}\{\varvec{u}\in \mathscr {D}_{\bar{T}}:\Vert \varvec{u}\Vert _{\mathscr {D}_{\bar{T}}}<R\}. \end{aligned}$$

Upon choosing $R>0$ sufficiently large, we can ensure that $\mathfrak {B}_{R;\bar{T}}\subset \mathscr {D}_{\bar{T}}$ is non-empty.

To achieve contractivity, we use the bilinearity of the equation. Let $\varvec{v}=(v,v',v^{\#}),\varvec{w}=(w,w',w^{\#})\in \mathscr {D}_{\bar{T}}$ and denote

$$\begin{aligned} (\Psi (\varvec{v}),\Psi (\varvec{v})',\Psi (\varvec{v})^\#){:}{=}\varvec{\Psi }(\varvec{v}),\qquad (\Psi (\varvec{w}),\Psi (\varvec{w})',\Psi (\varvec{w})^\#){:}{=}\varvec{\Psi }(\varvec{w}). \end{aligned}$$

We see thatwhereThe difference of the renormalised products is given byUsing the same bounds as before, we obtain, for some $\theta >0$,and

$$\begin{aligned}  &   \max \{\Vert \Psi (\varvec{v})^{\#}-\Psi (\varvec{w})^{\#}\Vert _{\mathscr {L}_{\eta ;\bar{T}}^{\kappa }\mathcal {C}^{\beta }},\Vert \Psi (\varvec{v})^{\#}-\Psi (\varvec{w})^{\#}\Vert _{\mathscr {L}_{2\eta ;\bar{T}}^{\kappa }\mathcal {C}^{\beta ^{\#}}}\}\\  &   \quad \lesssim \bar{T}^{\theta }\Vert \Omega ^{\#}(\varvec{v})-\Omega ^{\#}(\varvec{w})\Vert _{C_{2\eta ;\bar{T}}\mathcal {C}^{\alpha +\beta +2}}, \end{aligned}$$

as well as

$$\begin{aligned} \Vert \Psi (\varvec{v})'-\Psi (\varvec{w})'\Vert _{\mathscr {L}_{\eta ;\bar{T}}^{\kappa }\mathcal {C}^{\beta '}}\lesssim \Vert v-w\Vert _{\mathscr {L}_{\eta ;\bar{T}}^{\kappa }\mathcal {C}^{\beta }}. \end{aligned}$$

For the right hand side,By the same arguments as in the proof of Lemma 3.7 we haveCombining the bounds above, we obtain

3.10 $$\begin{aligned}&\max \{\Vert \Psi (\varvec{v})-\Psi (\varvec{w})\Vert _{\mathscr {L}_{\eta ;\bar{T}}^{\kappa }\mathcal {C}^{\beta }},\Vert \Psi (\varvec{v})^{\#}-\Psi (\varvec{w})^{\#}\Vert _{\mathscr {L}_{\eta ;\bar{T}}^{\kappa }\mathcal {C}^{\beta }},\Vert \Psi (\varvec{v})^{\#}-\Psi (\varvec{w})^{\#}\Vert _{\mathscr {L}_{2\eta ;\bar{T}}^{\kappa }\mathcal {C}^{\beta ^{\#}}}\}\nonumber \\&\qquad \lesssim \bar{T}^{\theta }\Vert \varvec{v}-\varvec{w}\Vert _{\mathscr {D}_{\bar{T}}}(1+\Vert \mathbb {X}\Vert _{\mathcal {X}^{\alpha ,\kappa }_{\bar{T}}}+\Vert v\Vert _{\mathscr {L}_{\eta ;\bar{T}}^{\kappa }\mathcal {C}^{\beta }}+\Vert w\Vert _{\mathscr {L}_{\eta ;\bar{T}}^{\kappa }\mathcal {C}^{\beta }})^{2}, \end{aligned}$$

3.11 $$\begin{aligned}&\Vert \Psi (\varvec{v})'-\Psi (\varvec{w})'\Vert _{\mathscr {L}_{\eta ;\bar{T}}^{\kappa }\mathcal {C}^{\beta '}}\lesssim \Vert v-w\Vert _{\mathscr {L}_{\eta ;\bar{T}}^{\kappa }\mathcal {C}^{\beta }}. \end{aligned}$$

Next we consider

$$\begin{aligned} (\Psi ^{\circ 2}(\varvec{v}),\Psi ^{\circ 2}(\varvec{v})',\Psi ^{\circ 2}(\varvec{v})^\#){:}{=}\varvec{\Psi }^{\circ 2}(\varvec{v}),\qquad (\Psi ^{\circ 2}(\varvec{w}),\Psi ^{\circ 2}(\varvec{w})',\Psi ^{\circ 2}(\varvec{w})^\#){:}{=}\varvec{\Psi }^{\circ 2}(\varvec{w}). \end{aligned}$$

Iterating (3.10)–(3.11), we arrive at

$$\begin{aligned} \begin{aligned}&\Vert \varvec{\Psi }^{\circ 2}(\varvec{v})-\varvec{\Psi }^{\circ 2}(\varvec{w})\Vert _{\mathscr {D}_{\bar{T}}}\\&\lesssim \bar{T}^{\theta }\Vert \varvec{v}-\varvec{w}\Vert _{\mathscr {D}_{\bar{T}}}(1+\Vert \mathbb {X}\Vert _{\mathcal {X}^{\alpha ,\kappa }_{\bar{T}}}+\Vert v\Vert _{\mathscr {L}_{\eta ;\bar{T}}^{\kappa }\mathcal {C}^{\beta }}+\Vert w\Vert _{\mathscr {L}_{\eta ;\bar{T}}^{\kappa }\mathcal {C}^{\beta }}\\&\qquad \qquad \qquad \qquad \qquad \qquad +\Vert \Psi (\varvec{v})\Vert _{\mathscr {L}_{\eta ;\bar{T}}^{\kappa }\mathcal {C}^{\beta }}+\Vert \Psi (\varvec{w})\Vert _{\mathscr {L}_{\eta ;\bar{T}}^{\kappa }\mathcal {C}^{\beta }})^{4}. \end{aligned} \end{aligned}$$

Assume $\varvec{v},\varvec{w}\in \mathfrak {B}_{R;\bar{T}}$. It follows by (3.7), the definition of $\bar{T}$ and $\mathfrak {B}_{R;\bar{T}}$, that

$$\begin{aligned} \Vert v\Vert _{\mathscr {L}_{\eta ;\bar{T}}^{\kappa }\mathcal {C}^{\beta }}<R,\qquad \Vert w\Vert _{\mathscr {L}_{\eta ;\bar{T}}^{\kappa }\mathcal {C}^{\beta }}<R,\qquad \Vert \Psi (\varvec{v})\Vert _{\mathscr {L}_{\eta ;\bar{T}}^{\kappa }\mathcal {C}^{\beta }}<R,\qquad \Vert \Psi (\varvec{w})\Vert _{\mathscr {L}_{\eta ;\bar{T}}^{\kappa }\mathcal {C}^{\beta }}<R. \end{aligned}$$

Choosing $\bar{T}$ still smaller, if necessary, we can arrange that for some $c<1$,

$$\begin{aligned} \Vert \varvec{\Psi }^{\circ 2}(\varvec{v})-\varvec{\Psi }^{\circ 2}(\varvec{w})\Vert _{\mathscr {D}_{\bar{T}}}<c\Vert \varvec{v}-\varvec{w}\Vert _{\mathscr {D}_{\bar{T}}}, \end{aligned}$$

showing that $\varvec{\Psi }^{\circ 2}$ is a contraction on $\mathfrak {B}_{R;\bar{T}}$.

By Banach’s fixed-point theorem there exists a unique fixed point for $\varvec{\Psi }^{\circ 2}$ in $\mathfrak {B}_{R;\bar{T}}$. It suffices to argue that a fixed point to $\varvec{\Psi }^{\circ 2}$ is also a fixed point to $\varvec{\Psi }$. The following is due to [49, Thm. 5.15]. Denote for the sake of notation, $\varvec{w}=\varvec{\Psi }^{\circ 2}(\varvec{w})$ and $\varvec{v}{:}{=}\varvec{\Psi }(\varvec{w})$. We have $\varvec{\Psi }^{\circ 2}(\varvec{v})=\varvec{\Psi }(\varvec{w})=\varvec{v}$, hence $\varvec{v}$ is itself a fixed point to $\varvec{\Psi }^{\circ 2}$, yielding by uniqueness that $\varvec{v}=\varvec{w}$. One can furthermore show that this fixed point is in fact unique in all of $\mathscr {D}_{\bar{T}}$; it suffices to compare two putative solutions in $\mathscr {D}_{\bar{T}}$ and similar estimates to those above show that they must be equal on a small time interval. Continuity then gives equality in all of $[0,\bar{T}]$. $\square$

The utility of Proposition 3.9 is that it allows us to show existence and uniqueness of a suitable notion of solution to (1.1) by setting . We call this solution paracontrolled and are now in a position to give a rigorous definition (cf. (1.11)–(1.12) for a formal motivation).

#### Definition 3.10

Let $\mathbb {X}\in \mathcal {X}^{\alpha ,\kappa }_{T}$ and $\rho _{0}\in \mathcal {B}_{p,q}^{\beta _{0}}$. Let $S\le T$, we call $\rho :[0,S]\rightarrow \mathcal {S}'(\mathbb {T}^{2})$ a paracontrolled solution to (1.1) on [0, *S*] with enhancement $\mathbb {X}$ and initial data $\rho _{0}$, if

- $$\varvec{w}{:}{=}(w,w',w^{\#})\in \mathscr {D}_{S}$$, where
, where the product $\nabla \cdot \mathcal {I}[\rho \nabla \Phi _{\rho }]$ needs to be interpreted in the renormalised sense, that is  with $\Omega ^{\#}(\varvec{w})$ defined as in Lemma 3.8.

In particular it follows by Definition 3.4, that $\lim _{t\rightarrow 0}\rho _{t}=\rho _{0}$ in $\mathcal {S}'(\mathbb {T}^{2})$.

We call $\rho :[0,S)\rightarrow \mathcal {S}'(\mathbb {T}^{2})$ a paracontrolled solution on [0, *S*), if $\rho$ is a paracontrolled solution on $[0,S']$ for every $S'<S$.

Using the uniqueness of the fixed point in Proposition 3.9, one can show that paracontrolled solutions are unique.

#### Lemma 3.11

Let $\mathbb {X}\in \mathcal {X}^{\alpha ,\kappa }_{T}$, $\rho _{0}\in \mathcal {B}_{p,q}^{\beta _{0}}$ and $S\le T$. Then there exists at most one paracontrolled solution $\rho :[0,S]\rightarrow \mathcal {S}'(\mathbb {T}^{2})$ to (1.1) on [0, *S*] with enhancement $\mathbb {X}$ and initial data $\rho _{0}$.

#### Proof

Consider two paracontrolled solutions $\rho$, ${\widetilde{\rho }}$, which by definition are associated to two triples $\varvec{w}$, $\widetilde{\varvec{w}}$. However, these triples must both satisfy by definition the same fixed-point equation (3.6), which has a unique solution by Proposition 3.9. Therefore both paracontrolled solutions must agree. $\square$

In the following lemma, we prove the existence of a paracontrolled solution given an abstract enhancement.

#### Lemma 3.12

Let $\mathbb {X}\in \mathcal {X}^{\alpha ,\kappa }_{T}$ and $\rho _0\in \mathcal {B}_{p,q}^{\beta _{0}}$. Let $\bar{T}\in (0,T\wedge 1]$ be as in Proposition 3.9 and let $\varvec{w}=(w,w',w^{\#})\in \mathscr {D}_{\bar{T}}$ be the fixed point of (3.6) constructed therein. Then,  is the unique paracontrolled solution to (1.1) on $[0,\bar{T}]$ with enhancement $\mathbb {X}$ and initial data $\rho _{0}$.

#### Proof

Let $\varvec{w}=(w,w',w^{\#})\in \mathscr {D}_{\bar{T}}$ be the fixed point to (3.6) and . It follows by definition,andHence, $\rho$ is a paracontrolled solution to (1.1) on $[0,\bar{T}]$ with enhancement $\mathbb {X}$ and initial data $\rho _{0}$, which is unique by Lemma 3.11.

$$\square $$

The next lemma shows that paracontrolled solutions are locally Lipschitz continuous in the noise enhancement and the initial data.

#### Lemma 3.13

Let $R>0$,  and $\rho _{0}^{\textsf {X}},\rho _{0}^{\textsf {Y}}\in \mathcal {B}_{p,q}^{\beta _{0}}$ be such that

$$\begin{aligned} \max \{\Vert \mathbb {X}\Vert _{\mathcal {X}^{\alpha ,\kappa }_{T}},\Vert \mathbb {Y}\Vert _{\mathcal {X}^{\alpha ,\kappa }_{T}},\Vert \rho _{0}^{\textsf {X}}\Vert _{\mathcal {B}_{p,q}^{\beta _{0}}},\Vert \rho _{0}^{\textsf {Y}}\Vert _{\mathcal {B}_{p,q}^{\beta _{0}}}\}<R. \end{aligned}$$

Then there exists some $\bar{T}=\bar{T}(R)\in (0,T]$ with the following properties

1. There exist solutions $\varvec{w}_{\textsf {X}}{:}{=}(w_{\textsf {X}},w'_{\textsf {X}},w^{\#}_{\textsf {X}})$ and $\varvec{w}_{\textsf {Y}}{:}{=}(w_{\textsf {Y}},w'_{\textsf {Y}},w^{\#}_{\textsf {Y}})$ to  and  on $[0,\bar{T}]$ by an application of Proposition 3.9. Here, $\Omega ^{\#}_{\textsf {X}}(\varvec{w}_{\textsf {X}})$ and $\Omega ^{\#}_{\textsf {Y}}(\varvec{w}_{\textsf {Y}})$ are defined as in Lemma 3.8 with noises $\mathbb {X}$, $\mathbb {Y}$ respectively.
2. Setting  one has

#### Proof

The claim follows as in Lemma 3.8, using the trilinearity of the equation.

$$\square $$

### Maximal time of existence

To establish a maximal time of existence for paracontrolled solutions to (1.1) (Theorem 3.15), we iterate the construction of Sect. 3.1. Let $(\alpha ,q,p,\beta ,\beta ',\beta ^{\#},\beta _{0},\kappa ,\eta )$ satisfy (3.1). Assume we have constructed a solution $\varvec{w}\in \mathscr {D}_{T_{1}}$ to (3.6) until time $T_{1}\in (0,T)$. Given the initial data  and an enhancement $\mathbb {X}\in \mathcal {X}^{\alpha ,\kappa }_{T}$, our paracontrolled Ansatz for the continuation is $\widetilde{\varvec{w}}=(\widetilde{w},\widetilde{w}',\widetilde{w}^{\#})$, where3.12with Gubinelli derivative3.13and paracontrolled remainder3.14where the renormalised product $\mathscr {P}_{T_{1}}(\widetilde{\varvec{w}},\mathbb {X})_{t}$ is given by3.15Hence, the only difference between the first iteration (Proposition 3.9) and subsequent steps is the initial data  in (3.14) and the additional termin the renormalised product (3.15).

#### Remark 3.14

The paracontrolled triple $\widetilde{\varvec{w}}$ of the second iteration step is related to the paracontrolled triple $\varvec{w}$ of the first iteration step by

$$\begin{aligned} \widetilde{w}_{t}=w_{T_{1}+t},\qquad \widetilde{w}'_{t}=w'_{T_{1}+t},\qquad \widetilde{w}^{\#}_{t}=w^{\#}_{T_{1}+t}+P_{t}(w_{T_{1}}-w^{\#}_{T_{1}}) \end{aligned}$$

(for those *t* such that both $\widetilde{\varvec{w}}_{t}$ and $\varvec{w}_{T_{1}+t}$ exist.) The additional terms in $\widetilde{w}^{\#}$ and $\mathscr {P}_{T_{1}}(\widetilde{\varvec{w}},\mathbb {X})$ ensure that the initial data in (3.14) can be expressed in terms of $\rho _{T_{1}}$,  and . Since we assumed $\mathbb {X}\in \mathcal {X}^{\alpha ,\kappa }_{T}$, this allows us to restart the equation as long as $\rho _{T_{1}}\in \mathcal {C}^{\alpha +1}$. The price to pay is that the dynamics of the paracontrolled remainder changes at $T_{1}$, hence the triple will no longer be continuous on the full time interval of existence. However, this will not be an issue, as we are primarily interested in the dynamics of the solutionwhich will still be continuous on the full time interval.

We can now use a technique inspired by [43, Derivation of (1.27)] to establish a maximal time of existence $T_{\text {max}}\le T$ and a paracontrolled solution on $[0,T_{\text {max}})$.

#### Theorem 3.15

Let $(\alpha ,q,p,\beta ,\beta ',\beta ^{\#},\beta _{0},\kappa ,\eta )$ satisfy (3.1). Let $T>0$, $\rho _{0}\in \mathcal {B}_{p,q}^{\beta _{0}}$ and $\mathbb {X}\in \mathcal {X}^{\alpha ,\kappa }_{T}$. Then there exists a $T_{\text {max}}\in (0,T]$ and a unique paracontrolled solution $\rho :[0,T_{\text {max}})\rightarrow \mathcal {S}'(\mathbb {T}^{2})$ to (1.1) on $[0,T_{\text {max}})$ with enhancement $\mathbb {X}$ and initial data $\rho _{0}\in \mathcal {B}_{p,q}^{\beta _{0}}$, such that

$$\begin{aligned} T_{\text {max}}=T\qquad \text {or}\qquad \lim _{t\uparrow T_{\text {max}}}\Vert \rho _{t}\Vert _{\mathcal {C}^{\alpha +1}}=\infty . \end{aligned}$$

#### Proof

Let $(\alpha ,q,p,\beta ,\beta ',\beta ^{\#},\beta _{0},\kappa ,\eta )$ satisfy (3.1). Since $\rho _{0}\in \mathcal {B}_{p,q}^{\beta _{0}}$, we can apply Proposition 3.9 and run the equation for some small time $\bar{t}_{0}=\bar{t}_{0}(\Vert \rho _{0}\Vert _{\mathcal {B}_{p,q}^{\beta _{0}}},\Vert \mathbb {X}\Vert _{\mathcal {X}^{\alpha ,\kappa }_{T}})\le T$. By construction, we obtain $\rho _{t_{0}}\in \mathcal {C}^{\alpha +1}$ for each $0<t_{0}\le \bar{t}_{0}$.

For the second iteration step, we choose a new tuple of exponents $(\alpha ,\infty ,\infty ,\beta _{1},\beta '_{1},\beta ^{\#}_{1},\alpha +1,\kappa ,\eta _{1})$ satisfying the assumptions (3.1) and3.16Using the assumption (3.16), we can control the additional term appearing in (3.15) via Bony’s estimates (Lemma A.4) and Schauder’s estimates (Lemma A.6).

Let $R>0$ be such that $\Vert \mathbb {X}\Vert _{\mathcal {X}^{\alpha ,\kappa }_{T}}\le R$ and assume that $0<t_{0}<\bar{t}_{0}$ satisfies $\Vert \rho _{t_{0}}\Vert _{\mathcal {C}^{\alpha +1}}\le R$. We can then use the decomposition (3.12)–(3.15) and an argument analogous to Proposition 3.9 to re-start the equation from $\rho _{t_{0}}$ and run it until time $t_{0}+T(R)$ for some $T(R)>0$.

Next, assume $\Vert \rho _{t_{0}+T(R)/2}\Vert _{\mathcal {C}^{\alpha +1}}\le R$, we can then re-start the equation at time $t_{0}+T(R)/2$ from $\rho _{t_{0}+T(R)/2}$ and run it until $t_{0}+T(R)/2+T(R)$ with the same exponents. We can then continue this procedure on the intervals

3.17 $$\begin{aligned}  &   [0,\bar{t}_{0}],\quad [t_{0},t_{0}+T(R)],\quad \Big [t_{0}+\frac{T(R)}{2},t_{0}+\frac{T(R)}{2}+T(R)\Big ],\quad \nonumber \\  &   \Big [t_{0}+T(R),t_{0}+2T(R)\Big ],\quad \ldots \end{aligned}$$

until the first time $t\in [t_{0},T]$ such that $\Vert \rho _{t}\Vert _{\mathcal {C}^{\alpha +1}}>R$, or, if such a *t* does not exist, until *T*. We denote this time horizon by $T_{\text {max}}^{(R)}$. This yields a paracontrolled solution $\rho :[0,T_{\text {max}}^{(R)}]\rightarrow \mathcal {S}'(\mathbb {T}^{2})$ to (1.1) with enhancement $\mathbb {X}$ and initial data $\rho _{0}\in \mathcal {B}_{p,q}^{\beta _{0}}$, where we used that the intervals (3.17) had some overlap as in [43, Derivation of (1.27)] to ensure that $\rho$ is independent of the choice of iteration used in its construction. Furthermore, the paracontrolled solution is unique by an application of Lemma 3.11.

The sequence $(T^{(R)}_{\max })_{R>0}$ is increasing, hence we may define $T_{\text {max}}{:}{=}\sup _{R>0}T_{\text {max}}^{(R)}=\lim _{R\rightarrow \infty }T_{\text {max}}^{(R)}\in (0,T]$. Assume $T_{\text {max}}<T$, then

$$\begin{aligned} \lim _{t\uparrow T_{\text {max}}}\Vert \rho _{t}\Vert _{\mathcal {C}^{\alpha +1}}=\lim _{R\uparrow \infty }\Vert \rho _{T_{\text {max}}^{(R)}}\Vert _{\mathcal {C}^{\alpha +1}}>\lim _{R\uparrow \infty }R=\infty . \end{aligned}$$

This yields the claim. $\square$

The principal issue in comparing paracontrolled solutions is that the maximal time of existence may depend on the noise enhancement. Hence, one needs to find a metric space of functions that can blow up at separate, finite times. A suitable space was introduced in [10, Sec. 1.5.1], whose construction we follow:

#### Definition 3.16

Let $(F,\Vert \,\cdot \,\Vert _{F})$ be a normed vector space and let  be a cemetery state. We define  and equip it with the topology generated by the open balls of $F$ and the sets of the form  with $R>0$.

Next we define the space $F^{\text {sol}}_{T}$ of continuous functions on [0, *T*] with values in  that cannot return from the cemetery state .

#### Definition 3.17

We define the set

We equip $F^{\text {sol}}_{T}$ with a suitable metric.

#### Lemma 3.18

There exists a metric $D_{T}$ on $F^{\text {sol}}_{T}$ such that $D_{T}(f_{n},f)\rightarrow 0$ as $n\rightarrow \infty$ if and only if for every $L>0$,

$$\begin{aligned} \lim _{n\rightarrow \infty }\sup _{t\in [0,T_{L}]}\Vert f_{n}(t)-f(t)\Vert _{F}=0, \end{aligned}$$

where

$$\begin{aligned} T_{L}{:}{=}T\wedge L\wedge \inf \{t\in [0,T]:\Vert f_{n}(t)\Vert _{F}>L~\text {or}~\Vert f(t)\Vert _{F}>L\}. \end{aligned}$$

#### Proof

The existence of such a metric follows by [10, Lem. 1.2]. $\square$

Finally, we equip $F^{\text {sol}}_{T}$ with a weight at 0 to allow for irregular initial data.

#### Definition 3.19

Let $\eta \ge 0$, we denote  for each  and $t\in (0,T]$. We defineand equip  with the metric  given by

Let $\alpha \in \mathbb {R}$ and $\eta \ge 0$, we denote the metric $D_{\eta ;T}$ on $(\mathcal {C}^{\alpha +1})^{\text {sol}}_{\eta ;T}$ by $D_{\eta ;T}^{\alpha +1}$.

In the following, we show that the paracontrolled solutions constructed in Theorem 3.15 are elements of $(\mathcal {C}^{\alpha +1})^{\text {sol}}_{\eta ;T}$.

#### Lemma 3.20

Let $(\alpha ,q,p,\beta ,\beta ',\beta ^{\#},\beta _{0},\kappa ,\eta )$ satisfy (3.1). Let $T>0$, $\rho _{0}\in \mathcal {B}_{p,q}^{\beta _{0}}$ and $\mathbb {X}\in \mathcal {X}^{\alpha ,\kappa }_{T}$. Let $\rho$ be the paracontrolled solution to (1.1) on $[0,T_{\text {max}})$ with enhancement $\mathbb {X}$ and initial data $\rho _{0}$, as constructed in Theorem 3.15. Then $\rho \in (\mathcal {C}^{\alpha +1})^{\text {sol}}_{\eta ;T}$ and, in particular, .

#### Proof

The paracontrolled solution constructed in Theorem 3.15 is continuous and exists until it blows up in $\mathcal {C}^{\alpha +1}$, from which the claim follows. $\square$

### The renormalised solution

In this subsection we combine the deterministic solution theory (Lemma 3.20) with the stochastic existence of the renormalised enhancement (Theorem 2.3) to construct the renormalised solution to (1.1) as a random variable (Theorem 3.22, Part 1). We then show that the renormalised solution is the limit in probability of solutions to (1.13) (Theorem 3.22, Part 2).

#### Definition 3.21

Let $(\alpha ,q,p,\beta ,\beta ',\beta ^{\#},\beta _{0},\kappa ,\eta )$ satisfy (3.1). Let $\rho _{0}\in \mathcal {B}^{\beta _0}_{p,q}$, $T>0$, $\sigma \in C_{T}\mathcal {H}^{2}$ and let $\mathbb {X}$ be the renormalised enhancement of Theorem 2.3. We call the paracontrolled solution $\rho \in (\mathcal {C}^{\alpha +1})_{\eta ;T}^{\text {sol}}$ to (1.1) with enhancement $\mathbb {X}$ and initial data $\rho _{0}$ the renormalised solution.

We can now prove the main result of this section, which is similar to [22, Cor. 4.7  & Cor. 5.9], [24, Thm. 3.7] and [9, Cor. 3.13].

#### Theorem 3.22

Let $(\alpha ,q,p,\beta ,\beta ',\beta ^{\#},\beta _{0},\kappa ,\eta )$ satisfy (3.1). Let $\rho _{0}\in \mathcal {B}^{\beta _0}_{p,q}$, $T>0$, $\sigma \in C_{T}\mathcal {H}^{2}$ and let $\mathbb {X}$ resp. $(\mathbb {X}^{\delta })_{\delta >0}$ be the (renormalised resp. prelimiting renormalised) enhancements of Theorem 2.3.

1. There exist paracontrolled solutions $\rho$, $(\rho ^{\delta })_{\delta >0}$ in $(\mathcal {C}^{\alpha +1})_{\eta ;T}^{\text {sol}}$ to (1.1) with enhancements $\mathbb {X}$, $(\mathbb {X}^{\delta })_{\delta >0}$ respectively and initial data $\rho _{0}$. The random variables $(\rho ^{\delta })_{\delta >0}$ coincide with the mild solutions (1.13).
2. It holds that $\rho ^{\delta }\rightarrow \rho$ in $(\mathcal {C}^{\alpha +1})^{\text {sol}}_{\eta ;T}$ in probability as $\delta \rightarrow 0$. In particular for each $\lambda >0$ one has that 3.18 where for every $L\in \mathbb {N}$, we denote $T_{L}{:}{=}T\wedge L\wedge \inf \{t\in [0,T]:\Vert \rho _{\eta \text {-wt}}(t)\Vert _{\mathcal {C}^{\alpha +1}}>L~\text {or}~\Vert \rho _{\eta \text {-wt}}^{\delta }(t)\Vert _{\mathcal {C}^{\alpha +1}}>L\}$.

#### Proof

Let $(\alpha ,q,p,\beta ,\beta ',\beta ^{\#},\beta _{0},\kappa ,\eta )$ satisfy (3.1). The enhancements $\mathbb {X}$ and $(\mathbb {X}^{\delta })_{\delta >0}$ exist almost surely by an application of Theorem 2.3. It then follows by Lemma 3.20 that $\rho$ and $(\rho ^{\delta })_{\delta >0}$ are random variables in $(\mathcal {C}^{\alpha +1})^{\text {sol}}_{\eta ;T}$. By Lemma 3.18 and the local Lipschitz continuity of the solution map in the enhancement, Lemma 3.13, we deduce that for every $\lambda >0$ there exists some $\nu >0$ such that $\Vert \mathbb {X}-\mathbb {X}^{\delta }\Vert _{\mathcal {X}^{\alpha ,\kappa }_{T}}\le \nu$ implies $D_{\eta ;T}^{\alpha +1}(\rho ,\rho ^{\delta })\le \lambda$.

Hence, by an application of Theorem 2.3,

$$\begin{aligned} \mathbb {P}\Big (D_{\eta ;T}^{\alpha +1}(\rho ,\rho ^{\delta })>\lambda \Big )\le \mathbb {P}(\Vert \mathbb {X}-\mathbb {X}^{\delta }\Vert _{\mathcal {X}^{\alpha ,\kappa }_{T}}>\nu )\rightarrow 0\quad \text {as}~\delta \rightarrow 0, \end{aligned}$$

which yields that $\rho ^{\delta }\rightarrow \rho$ in $(\mathcal {C}^{\alpha +1})_{\eta ;T}^{\text {sol}}$ in probability. The convergence (3.18) then follows by the explicit form of $D_{\eta ;T}^{\alpha +1}$, see [10, Sec. 1.5.1]. $\square$

#### Example 3.23

The same arguments as in the proof of Theorem 3.22, Part 1, also allow us to construct the mild solution to (1.3) as a paracontrolled solution to (1.1) with enhancement  (and heterogeneity $\sigma =\sqrt{\rho _{\text {det}}}$). To obtain a limit for vanishing correlation lengths $\delta$, one needs to assume that the noise intensity $\varepsilon$ decays sufficiently quickly depending on the behaviour of the renormalisation. This will be the focus of an upcoming work [41].

## Data Availability

N/A

## A Besov and Hölder–Besov spaces

Throughout the following section, all properties are given for mappings or distributions on $\mathbb {T}^d$ taking values in $\mathbb {R}^n$ for some $d,n\in \mathbb {N}$.

### A.1 Besov spaces

Applying essentially the same arguments as in the proof of [2, Prop. 2.10] there exists a *dyadic partition of unity*, i.e. a pair of non-negative, radially symmetric and compactly supported smooth functions $\varrho _{-1},\varrho _{0}\in C^{\infty }(\mathbb {R}^{d};[0,1])$ such that $\text {supp}(\varrho _{-1})\subset B(0,1/2)$ and $\text {supp}(\varrho _{0})\subset \{x\in \mathbb {R}^d:9/32\le |x|\le 1\}$. Furthermore, defining for each $k\in \mathbb {N}$, $\varrho _k(x){:}{=}\varrho _0(2^{-k}x)$, we assume it holds that

1. $$\text {supp}(\varrho _{k})\cap \text {supp}(\varrho _{l})=\emptyset $$   if    $$|k-l|\ge 2$$,
2. $$\sum _{k=-1}^{\infty }\varrho _{k}(x)=1$$   for any   $$x\in \mathbb {R}^d$$.

For $k\ge -1$ we define the Littlewood–Paley blocks to be the Fourier multipliers $\Delta _{k}u{:}{=}\mathscr {F}^{-1}(\varrho _{k}\mathscr {F}u)$ and set

$$\begin{aligned} \Delta _{<k}u{:}{=}\sum _{l=-1}^{k-1}\Delta _{l}u. \end{aligned}$$

As with the Fourier transform, we initially define these operators on smooth functions and then extend them by duality to $\mathcal {S}'(\mathbb {T}^d;\mathbb {R}^n)$.

#### Definition A.1

*(Besov spaces)* Let $\alpha \in \mathbb {R}$ and $p,q\in [1,\infty ]$. We define the nonhomogeneous Besov space $\mathcal {B}^{\alpha }_{p,q}(\mathbb {T}^d;\mathbb {R})$ to be the completion of the smooth functions $C^{\infty }(\mathbb {T}^d;\mathbb {R})$ under the norm

$$\begin{aligned} \Vert u\Vert _{\mathcal {B}^{\alpha }_{p,q}(\mathbb {T}^d;\mathbb {R})}{:}{=}\Vert (2^{k\alpha }\Vert \Delta _{k}u\Vert _{L^{p}(\mathbb {T}^d;\mathbb {R})})_{k\in \mathbb {N}_{-1}}\Vert _{\ell ^q}, \end{aligned}$$

which is extended to vector resp. matrix-valued functions in a natural componentwise manner. Here $\ell ^q$ denotes the usual space of *q*-summable sequences (or bounded when $q=\infty$). When $p=q=\infty$ we recall the shorthand $\mathcal {C}^{\alpha }(\mathbb {T}^d;\mathbb {R}^n){:}{=}\mathcal {B}^\alpha _{\infty ,\infty }(\mathbb {T}^d;\mathbb {R}^n)$ and call $\mathcal {C}^{\alpha }(\mathbb {T}^d;\mathbb {R}^n)$ the Hölder–Besov space.

#### Remark A.2

Note that the dyadic partition of unity obtained by [2, Prop. 2.10] is built from ${\widetilde{\varrho }}_{-1}$, ${\widetilde{\varrho }}_{0}$ with $\text {supp}({\widetilde{\varrho }}_{-1})\subset B(0,4/3)$ and $\text {supp}({\widetilde{\varrho }}_{0})\subset \{x\in \mathbb {R}^d:3/4\le |x|\le 8/3\}$. However, for our purposes it is convenient to rescale these functions by a factor of 3/8 so that the only integer in the support of $\varrho _{-1}$ is 0. Since the Besov spaces are independent of the chosen dyadic partition of unity [2, Cor. 2.70] this change is harmless.

The Besov spaces enjoy a number of useful properties which we list below. Proofs of the following statements can be found in [2, 22].

1. Embeddings: there exists a constant $C>0$ such that for any $\alpha \in \mathbb {R}$, $1\le p_1\le p_2\le \infty$ and $1\le q_1\le q_2 \le \infty$,
  A.1 $$\begin{aligned} \Vert u\Vert _{\mathcal {B}^{\alpha -d\left( 1/p_1-1/p_2\right) }_{p_2,q_2}} \le C \Vert u\Vert _{\mathcal {B}^{\alpha }_{p_1,q_1}}. \end{aligned}$$
  We also have the following continuous embeddings,
  $$\begin{aligned} \Vert u\Vert _{\mathcal {B}^\alpha _{p,q}}&\lesssim \Vert u\Vert _{\mathcal {B}^{\alpha '}_{p,q}},\quad \alpha<\alpha ' \in \mathbb {R},\\\Vert u\Vert _{\mathcal {B}^\alpha _{p,q}}&\lesssim \Vert u\Vert _{\mathcal {B}^{\alpha '}_{p,q'}},\quad \alpha<\alpha ' \in \mathbb {R},~q< q'\in [1,\infty ]. \end{aligned}$$
2. Relations to $L^p$-spaces: for $p\in [1,\infty ]$, one has,
  $$\begin{aligned} \Vert f\Vert _{\mathcal {B}^{0}_{p,\infty }}\lesssim \Vert f\Vert _{L^p}\lesssim \Vert f\Vert _{\mathcal {B}^{0}_{p,1}}. \end{aligned}$$

We regularly work in a scale of interpolation spaces which relate temporal and spatial regularity and are suitable for solutions to parabolic PDEs.

#### Definition A.3

*[Interpolation spaces]* Let $T>0$, $\eta \ge 0$, $\alpha \in \mathbb {R}$ and $\kappa \in (0,1]$. We define the norm

$$\begin{aligned} \Vert u\Vert _{\mathscr {L}_{\eta ;T}^{\kappa }\mathcal {C}^{\alpha }}{:}{=}\max \{\Vert u\Vert _{C_{\eta ;T}^{\kappa }\mathcal {C}^{\alpha -2\kappa }},\Vert u\Vert _{C_{\eta ;T}\mathcal {C}^{\alpha }}\}, \end{aligned}$$

and the spaces $\mathscr {L}_{\eta ;T}^{\kappa }\mathcal {C}^{\alpha }=C_{\eta ;T}^{\kappa }\mathcal {C}^{\alpha -2\kappa }\cap C_{\eta ;T}\mathcal {C}^{\alpha }$. We set $\mathscr {L}_{T}^{\kappa }\mathcal {C}^{\alpha }{:}{=}\mathscr {L}_{0;T}^{\kappa }\mathcal {C}^{\alpha }{:}{=}C_{T}^{\kappa }\mathcal {C}^{\alpha -2\kappa }\cap C_{T}\mathcal {C}^{\alpha }$ and by an abuse of notation understand $\mathscr {L}^{0}_{\eta ;T}\mathcal {C}^{\alpha } = C_{\eta ;T}\mathcal {C}^{\alpha }$.

### A.2 Paraproducts

For $u,v\in C^{\infty }(\mathbb {T}^d;\mathbb {R})$ we define the paraproduct  and resonant product  by

Formally one has the decomposition . Conditions under which this decomposition is valid for (time-dependent) distributions *u* and *v* are given by Bony’s estimates in the following lemma. These operators naturally extend to vector-valued and matrix-valued objects as either inner or outer products. Where the precise meaning is not clear from context it will be specified in the text.

#### Lemma A.4

(Bony’s estimates) Let $T>0$ and $\eta ,\eta _1,\eta _2\ge 0$ be such that $\eta =\eta _1+\eta _2$.

- Let $\beta \in \mathbb {R}$, $u\in C_{\eta _1;T}L^\infty (\mathbb {T}^d;\mathbb {R})$ and $v\in C_{\eta _2;T}\mathcal {C}^{\beta }(\mathbb {T}^d;\mathbb {R})$, then  and
- Let $\beta \in \mathbb {R}$, $\alpha <0$, $u\in C_{\eta _1;T}\mathcal {C}^{\alpha }(\mathbb {T}^d;\mathbb {R})$ and $v\in C_{\eta _2;T}\mathcal {C}^{\beta }(\mathbb {T}^d;\mathbb {R})$, then  and
- Let $\alpha ,\beta \in \mathbb {R}$ with $\alpha +\beta >0$, $u\in C_{\eta _1;T}\mathcal {C}^{\alpha }(\mathbb {T}^d;\mathbb {R})$ and $v\in C_{\eta _2;T}\mathcal {C}^{\beta }(\mathbb {T}^d;\mathbb {R})$, then  and

#### Proof

The result is a direct consequence of [22, Lem. 2.1]. $\square$

### A.3 Parabolic and elliptic regularity estimates

We will make use of the following interpolation inequality. Let $x\ge 0$ and $\gamma \in [0,1]$, then

A.2 $$\begin{aligned} 0\le 1-\textrm{e}\hspace{0.3888pt}^{-x}\le x^{\gamma }. \end{aligned}$$

We also apply the following rapid decay inequality. For any $r>0$, uniformly in $x\ge 0$,

A.3 $$\begin{aligned} x^{r}\textrm{e}\hspace{0.3888pt}^{-x}\lesssim 1. \end{aligned}$$

The operators *P*, $\mathcal {I}$ and $\Phi$ introduced in Sect. 1.1 and their accompanying kernels $\mathscr {H}$ and $\mathscr {G}$ can be generalized to $\mathbb {T}^{d}$ *mutatis mutandis*. For all $n\in \mathbb {N}$, we also apply $\mathcal {I}$ to $f=(f_1,\ldots ,f_n):[0,T]\times \mathbb {T}^{d}\rightarrow \mathbb {R}^{n}$ by setting $\mathcal {I}[f]=(\mathcal {I}[f_1],\ldots ,\mathcal {I}[f_n])$.

#### Lemma A.5

Let $\alpha \le \beta \in \mathbb {R}$ and $p,q,p',q'\in [1,\infty ]$ be such that $p\ge p'$ and $q\ge q'$. Then for any $t>0$,

A.4 $$\begin{aligned} \Vert P_tf\Vert _{\mathcal {B}^{\beta }_{p,q}}\lesssim (1\vee t^{-\frac{\beta -\alpha }{2}})(1\vee t^{-\frac{d}{2}\big (\frac{1}{p'}-\frac{1}{p}\big )})\Vert f\Vert _{\mathcal {B}_{p',q'}^{\alpha }}. \end{aligned}$$

Secondly, if $\alpha \le \beta \le \alpha +2$ then for any $t>0$,

A.5 $$\begin{aligned} \Vert (P_t-1) f\Vert _{\mathcal {B}^{\alpha }_{p,q}} \lesssim t^{\frac{\beta -\alpha }{2}} \Vert f\Vert _{\mathcal {B}^{\beta }_{p,q}}. \end{aligned}$$

#### Proof

We first show (A.4), which is an easy consequence of the Besov embedding and the regularising effect of the heat flow. By (A.1) for any $\beta \in \mathbb {R}$ and $p,q,p',q' \in [1,\infty ]$ with $p\ge p'$ and $q\ge q'$, it holds that,

$$\begin{aligned} \Vert P_tf\Vert _{\mathcal {B}^{\beta }_{p,q}} \lesssim \Vert P_tf\Vert _{\mathcal {B}^{\beta +d\left( 1/p'-1/p\right) }_{p',q'}}. \end{aligned}$$

We now apply the semigroup property and the regularizing effect of the heat flow ( [2, Lem. 2.4] & (A.3)),

$$\begin{aligned} \Vert P_{t}f\Vert _{\mathcal {B}^{\beta +d\left( 1/p'-1/p\right) }_{p',q'}}  &   \lesssim (1\vee t^{-\frac{d}{2}\big (\frac{1}{p'}-\frac{1}{p}\big )})\Vert P_{t/2}f\Vert _{\mathcal {B}^{\beta }_{p',q'}}\\  &   \lesssim (1\vee t^{-\frac{d}{2}\big (\frac{1}{p'}-\frac{1}{p}\big )})(1\vee t^{-\frac{\beta -\alpha }{2}})\Vert f\Vert _{\mathcal {B}^{\alpha }_{p',q'}}. \end{aligned}$$

This yields (A.4). The bound (A.5) can be found in [43, Prop. A.13], who cite [42, Prop. 6] for a proof in the full space. We provide a short argument. We consider the Littlewood–Paley blocks $\Delta _{k}(P_t-1)u=(P_t-1)\Delta _{k}u$ for $k\in \mathbb {N}_{-1}$. Since $(P_t-1)\Delta _{-1}u=0$, we may assume $k\in \mathbb {N}$. We apply [2, Lem. 2.4 & Lem. 2.1] to obtain the existence of some $c>0$ such that

$$\begin{aligned} \begin{aligned} \Vert (P_t-1)\Delta _{k}f\Vert _{L^p}&\le \int _{0}^{t}\Vert \partial _s P_s\Delta _{k}f\Vert _{L^p}\textrm{d}s=\int _{0}^{t}\Vert P_s\Delta \Delta _{k}f\Vert _{L^p}\textrm{d}s\\&\lesssim \Vert \Delta \Delta _k f\Vert _{L^{p}}\int _{0}^{t}\textrm{e}\hspace{0.3888pt}^{-cs2^{2k}}\textrm{d}s\lesssim \Vert \Delta _kf\Vert _{L^{p}}2^{2k}\int _{0}^{t}\textrm{e}\hspace{0.3888pt}^{-cs2^{2k}}\textrm{d}s\\&\lesssim \Vert \Delta _kf\Vert _{L^{p}}(1-\textrm{e}\hspace{0.3888pt}^{-ct2^{2k}})\lesssim \Vert \Delta _kf\Vert _{L^{p}}t^{\frac{\beta -\alpha }{2}}2^{k(\beta -\alpha )}. \end{aligned} \end{aligned}$$

In the last inequality, we applied (A.2) with $(\beta -\alpha )/2\in [0,1]$. This yields the claim using the definition of the $\mathcal {B}_{p,q}^{\alpha }$-norm.

$$\square $$

We can now establish Schauder estimates similar to [22, Lem. A.9] and [9, Prop. 2.7].

#### Lemma A.6

Let $T>0$, $\eta \ge 0$, $\alpha \le \beta \in \mathbb {R}$, $\kappa \in [0,1]$ and $p,q\in [1,\infty ]$. Then the following hold

1. If $$\frac{\beta -\alpha }{2}+\frac{d}{2p}\le \eta $$, then
  A.6 $$\begin{aligned} \Vert Pf\Vert _{\mathscr {L}_{\eta ;T}^{\kappa }\mathcal {C}^{\beta }}\lesssim (1\vee T^{-\frac{\beta -\alpha }{2}}) (1\vee T^{-\frac{d}{2p}})(1\wedge T)^{\eta }\Vert f\Vert _{\mathcal {B}_{p,q}^{\alpha }} \end{aligned}$$
  and $P:\mathcal {B}_{p,q}^{\alpha }\rightarrow \mathscr {L}_{\eta ;T}^{\kappa }\mathcal {C}^{\beta }$ is a continuous map.
2. If $\eta '\in [0,1)$, $\eta \le \eta '$ and $\beta <\alpha +2$ are such that $\frac{\beta -\alpha }{2} \vee \kappa \le 1-(\eta '-\eta )$, then
  A.7 $$\begin{aligned} \Vert \mathcal {I}[f]\Vert _{\mathscr {L}_{\eta ;T}^{\kappa }\mathcal {C}^{\beta }}\lesssim _{T}\Vert f\Vert _{C_{\eta ';T}\mathcal {C}^{\alpha }} \end{aligned}$$
  and $\mathcal {I}:C_{\eta ';T}\mathcal {C}^{\alpha }\rightarrow \mathscr {L}_{\eta ;T}^{\kappa }\mathcal {C}^{\beta }$ is a continuous map. In particular if $T\le 1$, then
  $$\begin{aligned} \Vert \mathcal {I}[f]\Vert _{\mathscr {L}_{\eta ;T}^{\kappa }\mathcal {C}^{\beta }}\lesssim ( T^{1-\frac{\beta -\alpha }{2}-(\eta '-\eta )}\vee T^{1-\kappa -(\eta '-\eta )})\Vert f\Vert _{C_{\eta ';T}\mathcal {C}^{\alpha }}. \end{aligned}$$
3. Furthermore, $\mathcal {I}:C_T\mathcal {C}^{\alpha }\rightarrow \mathscr {L}_T^{\kappa }\mathcal {C}^{\alpha +2}$ is a continuous map.

#### Proof

The proofs of (A.6) and (A.7) are simple consequences of Lemma A.5, the semigroup property and the definition of the interpolation spaces, Definition A.3. The continuity of $t\mapsto (1\wedge t)^{\eta }P_{t}u_0$ and $t\mapsto (1\wedge t)^{\eta }\mathcal {I}[f]_{t}$ follows from the fact that the same statement is true for smooth $u_{0}$, *f*, and then by taking limits along a smooth approximating sequence. $\square$

Assume $\theta :\mathbb {R}^d\rightarrow \mathbb {C}$ is smooth and such that for all multi-indices $\nu \in \mathbb {N}^d_{0}$, $\partial ^\nu \theta$ is of at most polynomial growth. Additionally assume that $\theta$ satisfies the *reality condition*

A.8 $$\begin{aligned} \overline{\theta (\omega )}=\theta (-\omega ),\qquad \omega \in \mathbb {Z}^{d}. \end{aligned}$$

We define the Fourier multiplier acting on $u\in \mathcal {S}'(\mathbb {T}^d;\mathbb {R})$ by the expression

$$\begin{aligned} \theta (D)u {:}{=}\mathscr {F}^{-1}(\theta \widehat{u}). \end{aligned}$$

The polynomial growth condition on all partial derivatives and (A.8) ensure that $\theta (D)$ maps real-valued distributions to real-valued distributions, a result which we generalize in the following lemma.

#### Lemma A.7

Let $\alpha \in \mathbb {R}$, $p,q\in [1,\infty ]$, $u\in \mathcal {B}^\alpha _{p,q}(\mathbb {T}^d;\mathbb {R})$, $k=2\lfloor 1+d/2\rfloor$ and assume that $\theta :\mathbb {R}^{d}\rightarrow \mathbb {C}$ satisfies $\theta \in C^{k}(\mathbb {R}^d\setminus \{0\};\mathbb {C})$, $\theta (0)=0$ and the reality condition (A.8). Assume there exists some $m\in \mathbb {R}$ and $C>0$, such that for any multi-index $\nu \in \mathbb {N}^d_0$ with $|\nu |\le k$,

$$\begin{aligned} |\partial ^\nu \theta (x)|\le C|x|^{m-|\nu |},\qquad x\in \mathbb {R}^d\setminus \{0\}. \end{aligned}$$

Then,

$$\begin{aligned} \Vert \theta (D)u\Vert _{\mathcal {B}^{\alpha -m}_{p,q}}\lesssim \Vert u\Vert _{\mathcal {B}^{\alpha }_{p,q}}. \end{aligned}$$

#### Proof

Since *u* is periodic the only frequency contained in the support of $\rho _{-1}$ is $\omega =0$, hence $\theta (D)\Delta _{-1}u=0$. The remaining Littlewood–Paley blocks can then be addressed directly with [2, Lem. 2.2]. $\square$

Lemma A.7 leads directly to a control on solutions to Poisson’s equation and their derivatives.

#### Lemma A.8

Let $u\in \mathcal {S}'(\mathbb {T}^d;\mathbb {R})$ be such that $\langle u,1\rangle _{L^{2}(\mathbb {T}^{d})}=0$. Then for any $\alpha \in \mathbb {R}$ and $p,q\in [1,\infty ]$,

$$\begin{aligned} \Vert \mathscr {G}*u\Vert _{\mathcal {B}^{\alpha }_{p,q}} \lesssim \Vert u\Vert _{\mathcal {B}^{\alpha -2}_{p,q}}, \qquad \Vert \nabla \mathscr {G}*u\Vert _{\mathcal {B}^{\alpha }_{p,q}}\lesssim \Vert u\Vert _{\mathcal {B}^{\alpha -1}_{p,q}}. \end{aligned}$$

#### Proof

Simply apply Lemma A.7 to the multipliers $\theta _1(\omega )= \frac{1}{|2\pi \omega |^{2}}\mathbb {1}_{\omega \ne 0}$ and $\theta _2(\omega )=\frac{2\pi \textrm{i}\hspace{0.3888pt}\omega }{|2\pi \omega |^{2}}\mathbb {1}_{\omega \ne 0}$. $\square$

### A.4 Commutator results

Many of the results presented below are analogues and simple extensions of similar results found in [9, 48] to time-weighted spaces, $C_{\eta ;T}\mathcal {C}^{\alpha }$.

#### Lemma A.9

Let $T>0$, $\eta ,\eta _1,\eta _2\ge 0$ such that $\eta =\eta _1+\eta _2$, $\alpha \in (-\infty ,1)$, $\beta \in \mathbb {R}$ and $k=2\lfloor 1+d/2\rfloor$. Assume $\theta :\mathbb {R}^{d}\rightarrow \mathbb {C}$ satisfies $\theta \in C^{k+1}(\mathbb {R}^d\setminus \{0\};\mathbb {C})$, $\theta (0)=0$ and (A.8). Assume there exists some $m\in \mathbb {R}$ and $C>0$, such that for any multi-index $\nu \in \mathbb {N}^d_0$ with $|\nu |\le k+1$,

$$\begin{aligned} |\partial ^\nu \theta (x)|\le C|x|^{m-|\nu |},\qquad x\in \mathbb {R}^d\setminus \{0\}. \end{aligned}$$

Let $u\in C_{\eta _1;T}\mathcal {C}^{\alpha }(\mathbb {T}^{d};\mathbb {R})$ and $v\in C_{\eta _2;T}\mathcal {C}^{\beta }(\mathbb {T}^{d};\mathbb {R})$, then  and

#### Proof

The result is a simple extension of [48, Lem. 5.3.20] and [9, Lem. A.1] to functions with prescribed blow-up in $\mathcal {C}^{\alpha }$ at $t=0$. $\square$

Next, we consider the commutator between the operators $\mathcal {I}$ and , a result reminiscent of [9, Prop. 2.7].

#### Lemma A.10

Let $T>0$, $\eta ,\eta _{1},\eta _{2}\in [0,1)$ such that $\eta =\eta _1+\eta _2$, $\kappa >0$, $\alpha \in (-\infty ,(1\wedge 2\kappa ))$, $\beta \in \mathbb {R}$ and $m\in (0,2)$. Let $u\in \mathscr {L}_{\eta _1;T}^{\kappa }\mathcal {C}^{\alpha }(\mathbb {T}^{d};\mathbb {R})$ and $v\in C_{\eta _2;T}\mathcal {C}^{\beta }(\mathbb {T}^{d};\mathbb {R})$, then  and

A.9

#### Proof

Let $u\in \mathscr {L}_{\eta _1;T}^{\kappa }\mathcal {C}^{\alpha }$ and $v\in C_{\eta _2;T}\mathcal {C}^{\beta }$, we first prove the bound (A.9) and then the regularity . Let $t\in [0,T]$, by definition,

To bound the first summand, we apply [48, Lem. 5.3.20], and use that $\alpha <1$, to estimate

Taking the supremum, we obtain

We can use a case distinction over $t\in [0,1]$ and $t\in (1,\infty )$; and the assumptions $\eta <1$, $m<2$, to bound

$$\begin{aligned} \sup _{t\in [0,T]}(1\wedge t)^{\eta }\int _{0}^{t}|t-s|^{-m/2}(1\wedge s)^{-\eta }\textrm{d}s\lesssim _{T}1. \end{aligned}$$

To bound the second summand, we apply Lemma A.5, and use that $\alpha -2\kappa <0$, to estimate

Taking the supremum, we obtain

We can use a case distinction over $t\in [0,1]$, $t\in (1,2]$, and $t\in (2,\infty )$; and the assumptions $\eta <1$, $m<2$, to bound

$$\begin{aligned} \sup _{t\in [0,T]}(1\wedge t)^{\eta }\int _{0}^{t}|t-s|^{\kappa }(1\vee |t-s|^{-\kappa -m/2})(1\wedge s)^{-\eta }\textrm{d}s\lesssim _{T}1. \end{aligned}$$

It follows that

which yields (A.9).

To establish , we need to show that  is continuous and that  exists in $\mathcal {C}^{\alpha +\beta +m}$.

The continuity in (0, *T*] follows by the completeness of the continuous functions under the supremum norm and by taking limits along a smooth approximating sequence of *v*.

To show the continuity at 0, we can use the same bounds as in the derivation of (A.9) to control for every $t\in (0,1\wedge T]$,

which implies . Therefore, , which yields the claim.

$$\square $$

Finally, we present a commutator result between the operators  and .

#### Lemma A.11

Let $T>0$, $\eta ,\eta _1,\eta _2,\eta _3\ge 0$ such that $\eta =\eta _1+\eta _2+\eta _3$, $\alpha \in (0,1)$ and $\beta ,\gamma \in \mathbb {R}$ such that $\beta +\gamma <0$ and $\alpha +\beta +\gamma >0$. We define

Then $\mathscr {C}$ extends to a bounded, trilinear operator $\mathscr {C}:C_{\eta _1;T}\mathcal {C}^{\alpha }\times C_{\eta _2;T}\mathcal {C}^{\beta }\times C_{\eta _3;T}\mathcal {C}^{\gamma }\rightarrow C_{\eta ;T}\mathcal {C}^{\alpha +\beta +\gamma }$.

#### Proof

The result is a direct consequence of [22, Lem. 2.4]. $\square$

## B Shape coefficient estimates

In this section we bound our shape coefficients in terms of the time regularity $|t-s|^{\gamma }$ and the space regularity $|\omega _k|^{\beta }$ for some $\gamma \in [0,1]$ and $\beta \in \mathbb {R}$. We first consider the shape coefficient for .

### Lemma B.1

Let $s,t\ge 0$, $\gamma \in [0,1]$ and $\omega _1,\omega _2\in 2\pi \mathbb {Z}^2\setminus \{0\}$ be such that $\omega _1+\omega _2\not =0$.

1. In the case $(\omega _1\perp \omega _2)$, we obtain
2. In the case $\lnot (\omega _1\perp \omega _2)$, we obtain

The implied constants are uniform in $\omega _1$, $\omega _2$.

### Proof

To bound our shape coefficients, we first derive explicit expressions, a step that was inspired by [51, Proof of Thm. 2.1]. To do so, we evaluate the exponential integrals in (2.12) and compute  through (2.13). We can then further decompose

into the *leading term*  and the *error term* . The error term is generated by the zero initial condition of the noise, i.e. the remaining restriction $u_3,u_3'\ge 0$ in (2.12).

Assume $$(\omega _1\perp \omega _2)$$, then

and

We first consider  and estimate by (A.2) for $\gamma \in [0,1]$,

Next we estimate the error term . By symmetry it is enough to consider $s\le t$, for which

$$\begin{aligned} \textrm{e}\hspace{0.3888pt}^{-(t+s)|\omega _1+\omega _2|^2}-\textrm{e}\hspace{0.3888pt}^{-2s|\omega _1+\omega _2|^2}\le 0. \end{aligned}$$

We can then proceed to bound the remaining non-negative term of  by (A.2) and (A.3), giving the result in the case $(\omega _1\perp \omega _2)$.

Next assume $\lnot (\omega _1\perp \omega _2)$. We obtain the expressions

B.1

and

B.2

We first consider (B.1). The first term

$$\begin{aligned} \frac{1}{|\omega _1|^2+|\omega _2|^2-|\omega _1+\omega _2|^2}(\textrm{e}\hspace{0.3888pt}^{-|t-s|(|\omega _1|^2+|\omega _2|^2)}-\textrm{e}\hspace{0.3888pt}^{-|t-s||\omega _1+\omega _2|^2}) \end{aligned}$$

is non-positive so that by (A.2),

In (B.2), we bound the second, non-positive term by 0. In the first term, we distinguish cases to fix the sign of the prefactor $(|\omega _1|^2+|\omega _2|^2-|\omega _1+\omega _2|^2)^{-1}$. Assume first $|\omega _1|^2+|\omega _2|^2<|\omega _1+\omega _2|^2$ and by symmetry $s\le t$.

We obtain by (A.2) and (A.3),

B.3 $$\begin{aligned} \begin{aligned}&\frac{1}{|\omega _1|^2+|\omega _2|^2-|\omega _1+\omega _2|^2}\frac{1}{|\omega _1|^2+|\omega _2|^2+|\omega _1+\omega _2|^2}\\&\quad \times \Big ((\textrm{e}\hspace{0.3888pt}^{t(|\omega _1+\omega _2|^2-|\omega _1|^2-|\omega _2|^2)}-1)(\textrm{e}\hspace{0.3888pt}^{-2t|\omega _1+\omega _2|^2}-\textrm{e}\hspace{0.3888pt}^{-(t+s)|\omega _1+\omega _2|^2})\\&\quad +(\textrm{e}\hspace{0.3888pt}^{s(|\omega _1+\omega _2|^2-|\omega _1|^2-|\omega _2|^2)}-1)(\textrm{e}\hspace{0.3888pt}^{-2s|\omega _1+\omega _2|^2}-\textrm{e}\hspace{0.3888pt}^{-(t+s)|\omega _1+\omega _2|^2})\Big )\\&\le \frac{1}{|\omega _1+\omega _2|^2-|\omega _1|^2-|\omega _2|^2}\frac{1}{|\omega _1|^2+|\omega _2|^2+|\omega _1+\omega _2|^2}\\&\quad \times \textrm{e}\hspace{0.3888pt}^{-(t+s)(|\omega _1|^2+|\omega _2|^2)}(1-\textrm{e}\hspace{0.3888pt}^{-t(|\omega _1+\omega _2|^2-|\omega _1|^2-|\omega _2|^2)})(1-\textrm{e}\hspace{0.3888pt}^{-(t-s)|\omega _1+\omega _2|^2})\\&\lesssim |t-s|^{\gamma }\frac{|\omega _1+\omega _2|^{2\gamma }}{|\omega _1|^2+|\omega _2|^2+|\omega _1+\omega _2|^2}\frac{1}{|\omega _1|^2+|\omega _2|^2}\frac{t}{t+s}\\&\lesssim |t-s|^{\gamma }|\omega _1+\omega _2|^{-2+2\gamma }|\omega _1|^{-2}. \end{aligned} \end{aligned}$$

If instead $|\omega _1+\omega _2|^2<|\omega _1|^2+|\omega _2|^2$, $s\le t$, then

B.4 $$\begin{aligned} \begin{aligned}&\frac{1}{|\omega _1|^2+|\omega _2|^2-|\omega _1+\omega _2|^2}\frac{1}{|\omega _1|^2+|\omega _2|^2+|\omega _1+\omega _2|^2}\\&\quad \times \Big ((\textrm{e}\hspace{0.3888pt}^{t(|\omega _1+\omega _2|^2-|\omega _1|^2-|\omega _2|^2)}-1)(\textrm{e}\hspace{0.3888pt}^{-2t|\omega _1+\omega _2|^2}-\textrm{e}\hspace{0.3888pt}^{-(t+s)|\omega _1+\omega _2|^2})\\&\qquad \quad +(\textrm{e}\hspace{0.3888pt}^{s(|\omega _1+\omega _2|^2-|\omega _1|^2-|\omega _2|^2)}-1)(\textrm{e}\hspace{0.3888pt}^{-2s|\omega _1+\omega _2|^2}-\textrm{e}\hspace{0.3888pt}^{-(t+s)|\omega _1+\omega _2|^2})\Big )\\&\le \frac{1}{|\omega _1|^2+|\omega _2|^2-|\omega _1+\omega _2|^2}\frac{1}{|\omega _1|^2+|\omega _2|^2+|\omega _1+\omega _2|^2}\\&\quad \times (1-\textrm{e}\hspace{0.3888pt}^{-t(|\omega _1|^2+|\omega _2|^2-|\omega _1+\omega _2|^2)})\textrm{e}\hspace{0.3888pt}^{-(t+s)|\omega _1+\omega _2|^2}(1-\textrm{e}\hspace{0.3888pt}^{-(t-s)|\omega _1+\omega _2|^2})\\&\lesssim |t-s|^{\gamma }|\omega _1+\omega _2|^{-2}\frac{|\omega _1+\omega _2|^{2\gamma }}{|\omega _1|^2+|\omega _2|^2+|\omega _1+\omega _2|^2}\frac{t}{t+s}\\&\le |t-s|^{\gamma }|\omega _1+\omega _2|^{-2}|\omega _1|^{-2+2\gamma }. \end{aligned} \end{aligned}$$

By combining (B.2) with (B.3) and (B.4), we arrive at

This yields the claim. $\square$

Next we bound the shape coefficient , which appears in , ,  and .

### Lemma B.2

Let $s,t\ge 0$, $k=1,2$, $\gamma \in [0,1]$ and $C\ge 1$. Then uniformly in $\omega _1,\omega _2,\omega _3\in \mathbb {Z}^2\setminus \{0\}$ such that $C^{-1}|\omega _1|\le |\omega _2|\le C|\omega _1|$, it holds that

### Proof

The claim follows by the triangle inequality and repeated applications of (A.2). $\square$

The following lemma controls the shape coefficient , which appears in ,  and , .

### Lemma B.3

Let $s,t\ge 0$, $k,k'=1,2$, $\gamma \in [0,1]$ and $C,C'\ge 1$. Then uniformly in $\omega _1,\omega _1',\omega _2\in \mathbb {Z}^2\setminus \{0\}$ such that $C^{-1}|\omega _1|\le |\omega _2|\le C|\omega _1|$ and $(C')^{-1}|\omega _1'|\le |\omega _2|\le C'|\omega _1'|$, it holds that

### Proof

The claim follows by the triangle inequality and repeated applications of (A.2). $\square$

## C Summation estimates

### C.1 Basic estimates

We prove a number of summation and discrete convolution estimates that are central to our bounds.

Recall that $G^{j}(\omega )=2\pi \textrm{i}\hspace{0.3888pt}\omega ^{j}|2\pi \textrm{i}\hspace{0.3888pt}\omega |^{-2}\mathbb {1}_{\omega \ne 0}$ for all $\omega \in \mathbb {Z}^{2}$ and $j=1,2$. The following lemma shows that $|G^{j}(\omega +\omega _1)-G^{j}(\omega _1)|$ has better decay in $\omega +\omega _1$ than $|G^{j}(\omega +\omega _1)|$.

#### Lemma C.1

Let $j=1,2$. Then uniformly in $\omega ,\omega _1\in \mathbb {Z}^2$ such that $\omega _1,\omega +\omega _1\ne 0$, it holds that

$$\begin{aligned} |G^{j}(\omega +\omega _1)-G^{j}(\omega _1)|\lesssim |\omega ||\omega +\omega _1|^{-2}(1+|\omega ||\omega _1|^{-1}). \end{aligned}$$

#### Proof

We compute

$$\begin{aligned} G^{j}(\omega +\omega _1)-G^{j}(\omega _1)=\frac{\textrm{i}\hspace{0.3888pt}}{2\pi }\Big (\frac{\omega ^j+\omega _1^j}{|\omega +\omega _1|^2}-\frac{\omega _1^j}{|\omega _1|^2}\Big ), \end{aligned}$$

which can be bounded in absolute value by

$$\begin{aligned}  &   \Bigg |\frac{\omega ^j+\omega _1^j}{|\omega +\omega _1|^2}-\frac{\omega _1^j}{|\omega _1|^2}\Bigg |=\frac{|\omega ^j|\omega _1|^2-\omega _1^j|\omega |^2-\omega _1^j2\langle \omega ,\omega _1\rangle |}{|\omega +\omega _1|^2|\omega _1|^2}\\  &   \quad \lesssim |\omega ||\omega +\omega _1|^{-2}+|\omega |^2|\omega _1|^{-1}|\omega +\omega _1|^{-2}. \end{aligned}$$

This yields the claim.

$$\square $$

We apply the following summation estimates repeatedly to establish the regularities of our diagrams.

#### Lemma C.2

It holds that uniformly in $\delta >0$,

C.1 $$\begin{aligned} \sum _{\begin{array}{c} k\in \mathbb {Z}^2\\ |k|\le \delta ^{-1} \end{array}}1\lesssim (1\vee \delta ^{-2}) \end{aligned}$$

and

C.2 $$\begin{aligned} \sum _{\begin{array}{c} k\in \mathbb {Z}^2\setminus \{0\}\\ |k|\le \delta ^{-1} \end{array}}|k|^{-2}\lesssim (1\vee \log (\delta ^{-1})). \end{aligned}$$

What is more, for any $\alpha >2$,

C.3 $$\begin{aligned} \sum _{k\in \mathbb {Z}^2\setminus \{0\}}|k|^{-\alpha }\lesssim _{\alpha }1. \end{aligned}$$

We make repeated use of the following convolution estimate to construct non-linear objects.

#### Lemma C.3

([55, Lem. 3.10], [44, Lem. 5 & Lem. 6])

Let $\alpha ,\beta \in \mathbb {R}$ be such that $\alpha +\beta >2$. We have uniformly in $\omega \in \mathbb {Z}^2$,

$$\begin{aligned} \sum _{\begin{array}{c} \omega _1\in \mathbb {Z}^{2}\setminus \{0,\omega \}\\ \omega _1\sim (\omega -\omega _1) \end{array}}|\omega _1|^{-\alpha }|\omega -\omega _1|^{-\beta }\lesssim _{\alpha +\beta }(1\vee |\omega |)^{-\alpha -\beta +2}. \end{aligned}$$

If in addition $\alpha ,\beta <2$, then we have uniformly in $\omega \in \mathbb {Z}^2$,

$$\begin{aligned} \sum _{\omega _1\in \mathbb {Z}^{2}\setminus \{0,\omega \}}|\omega _1|^{-\alpha }|\omega -\omega _1|^{-\beta }\lesssim _{\alpha ,\beta ,\alpha +\beta }(1\vee |\omega |)^{-\alpha -\beta +2}. \end{aligned}$$

The next convolution result is useful for estimating correlated frequencies $\omega \not =\omega '$.

#### Lemma C.4

Let $\alpha ,\beta ,\gamma \in (0,2)$ be such that $\alpha +\gamma >2$ and $\beta +\gamma >2$. We have uniformly in $\omega ,\omega '\in \mathbb {Z}^2\setminus \{0\}$ such that $\omega \ne \omega '$,

$$\begin{aligned}  &   \sum _{\omega _1\in \mathbb {Z}^2\setminus \{0,\omega ,\omega '\}}|\omega -\omega _1|^{-\alpha }|\omega '-\omega _1|^{-\beta }|\omega _1|^{-\gamma }\\  &   \quad \lesssim |\omega -\omega '|^{-\beta }|\omega |^{-\alpha -\gamma +2}+|\omega -\omega '|^{-\alpha }|\omega '|^{-\beta -\gamma +2}. \end{aligned}$$

#### Proof

The proof follows by two applications of Lemma C.3, one in the case $|\omega -\omega _1|\le |\omega -\omega '|/2$ and the other in the complement. $\square$

To derive finer estimates, it is useful to introduce a discrete paraproduct analogue to extend Lemma C.3.

#### Lemma C.5

Let $\alpha ,\beta \in \mathbb {R}$ be such that $\alpha >2$ and $\beta \ge 0$. We have uniformly in $\omega \in \mathbb {Z}^2\setminus \{0\}$,

$$\begin{aligned} \sum _{\begin{array}{c} \omega _1\in \mathbb {Z}^{2}\setminus \{0,\omega \}\\ \omega _1\precsim (\omega -\omega _1) \end{array}}|\omega _1|^{-\alpha }|\omega -\omega _1|^{-\beta }\lesssim _{\alpha }|\omega |^{-\beta }. \end{aligned}$$

The proof is immediate by (C.3) and the bound induced by (1.8).

### C.2 Double sum estimates

When we take the Fourier transform of the noise $\sigma \varvec{\xi }$, it generates convolutions of ${\widehat{\sigma }}(t,\omega -m_1)$ against $\textrm{d}W^{j_1}(t,m_1)$ in $m_1\in \mathbb {Z}^{2}$. We also generate convolutions in $\omega _k\in \mathbb {Z}^{2}$ by constructing non-linear objects such as . Those steps lead to double sums over $\omega _k$ and $m_k$ that do not factorize. In this section, we estimate those sums.

We apply the following estimate in Sect. 2.5 to construct .

#### Lemma C.6

Let $\gamma \in [0,1/2)$ and $\varepsilon \in (0,1-2\gamma )$. Then uniformly in $\omega ,\omega _1\in \mathbb {Z}^{2}$, it holds that

$$\begin{aligned} \begin{aligned}&\sum _{\begin{array}{c} \omega _4\in \mathbb {Z}^{2}\setminus \{0,\omega ,\omega -\omega _1\}\\ (\omega -\omega _4)\sim \omega _4 \end{array}}|\omega -\omega _4|^{-2}(1+|\omega ||\omega _4|^{-1})|\omega _4|^{2\gamma }|\omega -\omega _1-\omega _4|^{-1}\\&\quad \times \sum _{m_2\in \mathbb {Z}^{2}}(1+|\omega -\omega _1-\omega _4-m_2|^2)^{-1}(1+|\omega _4+m_2|^2)^{-1}\\&\lesssim (1\vee |\omega -\omega _1|)^{-2+\varepsilon }(1\vee |\omega |)^{-1+2\gamma +\varepsilon }. \end{aligned} \end{aligned}$$

#### Proof

We decompose the sum over $m_2\in \mathbb {Z}^{2}$ into the regions $m_2=\omega -\omega _1-\omega _4$, $m_2=-\omega _4$ and $m_2\in \mathbb {Z}^{2}\setminus \{\omega -\omega _1-\omega _4,-\omega _4\}$.

We only give the bound that involves the sum over $m_2\in \mathbb {Z}^{2}\setminus \{\omega -\omega _1-\omega _4,-\omega _4\}$. We estimate

$$\begin{aligned} \begin{aligned}&\sum _{m_2\in \mathbb {Z}^{2}\setminus \{\omega -\omega _1-\omega _4,-\omega _4\}}(1+|\omega -\omega _1-\omega _4-m_2|^{2})^{-1}(1+|\omega _4+m_2|^{2})^{-1}\\&\le \sum _{m_2\in \mathbb {Z}^{2}\setminus \{\omega -\omega _1-\omega _4,-\omega _4\}}|\omega -\omega _1-\omega _4-m_2|^{-2}|\omega _4+m_2|^{-2}. \end{aligned} \end{aligned}$$

Let $\varepsilon \in (0,1)$. We can then apply Lemma C.3,

$$\begin{aligned} \begin{aligned}&\sum _{\begin{array}{c} \omega _4\in \mathbb {Z}^{2}\setminus \{0,\omega ,\omega -\omega _1\}\\ (\omega -\omega _4)\sim \omega _4 \end{array}}|\omega -\omega _4|^{-2}(1+|\omega ||\omega _4|^{-1})|\omega _4|^{2\gamma }|\omega -\omega _1-\omega _4|^{-1}\\&\quad \times \sum _{m_2\in \mathbb {Z}^{2}\setminus \{\omega -\omega _1-\omega _4,-\omega _4\}}|\omega -\omega _1-\omega _4-m_2|^{-2}|\omega _4+m_2|^{-2}\\&\lesssim (1\vee |\omega -\omega _1|)^{-2+2\varepsilon }\sum _{\begin{array}{c} \omega _4\in \mathbb {Z}^{2}\setminus \{0,\omega ,\omega -\omega _1\}\\ (\omega -\omega _4)\sim \omega _4 \end{array}}|\omega -\omega _4|^{-2}(1+|\omega ||\omega _4|^{-1})|\omega _4|^{2\gamma }|\omega -\omega _1-\omega _4|^{-1}. \end{aligned} \end{aligned}$$

Let $p\in (1,\infty )$, $q=p/(p-1)$, $\delta >0$. By Hölder’s inequality,

$$\begin{aligned} \begin{aligned}&\sum _{\begin{array}{c} \omega _4\in \mathbb {Z}^{2}\setminus \{0,\omega ,\omega -\omega _1\}\\ (\omega -\omega _4)\sim \omega _4 \end{array}}|\omega -\omega _4|^{-2}(1+|\omega ||\omega _4|^{-1})|\omega _4|^{2\gamma }|\omega -\omega _1-\omega _4|^{-1}\\&\le \Big (\sum _{\begin{array}{c} \omega _4\in \mathbb {Z}^{2}\setminus \{0,\omega ,\omega -\omega _1\}\\ (\omega -\omega _4)\sim \omega _4 \end{array}}|\omega -\omega _4|^{-2p}|\omega _4|^{\delta p}(1+|\omega ||\omega _4|^{-1})^{p}\Big )^{1/p}\\&\quad \times \Big (\sum _{\omega _4\in \mathbb {Z}^{2}\setminus \{0,\omega ,\omega -\omega _1\}}|\omega _4|^{-\delta q}|\omega _4|^{2\gamma q}|\omega -\omega _1-\omega _4|^{-q}\Big )^{1/q}. \end{aligned} \end{aligned}$$

We assume $\gamma \in [0,1/2)$, $2<p$ and $1-2/p+2\gamma<\delta <2-2/p$. It follows

$$\begin{aligned} p(2-\delta )>2,\qquad q(\delta -2\gamma )<2,\qquad q<2,\qquad q(\delta -2\gamma +1)>2. \end{aligned}$$

Consequently, by Lemma C.3,

$$\begin{aligned} \begin{aligned}&\Big (\sum _{\begin{array}{c} \omega _4\in \mathbb {Z}^{2}\setminus \{0,\omega ,\omega -\omega _1\}\\ (\omega -\omega _4)\sim \omega _4 \end{array}}|\omega -\omega _4|^{-2p}|\omega _4|^{\delta p}(1+|\omega ||\omega _4|^{-1})^{p}\Big )^{1/p}\\&\quad \times \Big (\sum _{\omega _4\in \mathbb {Z}^{2}\setminus \{0,\omega ,\omega -\omega _1\}}|\omega _4|^{-\delta q}|\omega _4|^{2\gamma q}|\omega -\omega _1-\omega _4|^{-q}\Big )^{1/q}\\&\lesssim (1\vee |\omega |)^{-2+\delta +2/p}(1\vee |\omega -\omega _1|)^{-\delta +2\gamma -1+2/q}. \end{aligned} \end{aligned}$$

Let $\varepsilon \in (0,1-2\gamma )$. We can now set $\delta =1-2/p+2\gamma +\varepsilon$ to conclude

$$\begin{aligned}  &   \sum _{\begin{array}{c} \omega _4\in \mathbb {Z}^{2}\setminus \{0,\omega ,\omega -\omega _1\}\\ (\omega -\omega _4)\sim \omega _4 \end{array}}|\omega -\omega _4|^{-2}(1+|\omega ||\omega _4|^{-1})|\omega _4|^{2\gamma }|\omega -\omega _1-\omega _4|^{-1}\\  &   \quad \lesssim (1\vee |\omega |)^{-1+2\gamma +\varepsilon }(1\vee |\omega -\omega _1|)^{-\varepsilon }. \end{aligned}$$

This yields the claim. $\square$

We apply the following estimate in Sect. 2.6 to construct ,  and . We use the restriction $|m_1|\le \delta ^{-1}$ induced by the cut-off $\varphi (\delta m_1)$ to establish a bound in terms of $\log (\delta ^{-1})$.

#### Lemma C.7

Let $\varepsilon \in (0,1/2)$ and $\delta >0$. Then uniformly in $\omega \in \mathbb {Z}^{2}\setminus \{0\}$ it holds that

C.4 $$\begin{aligned} \begin{aligned}&\sum _{\begin{array}{c} m_1\in \mathbb {Z}^{2}\\ |m_1|\le \delta ^{-1} \end{array}}\sum _{\omega _1\in \mathbb {Z}^{2}\setminus \{0,\omega \}}(1+|\omega _1-m_1|^{2})^{-1}(1+|\omega -\omega _1+m_1|^{2})^{-1}|\omega _1|^{-2}(1+|\omega ||\omega -\omega _1|^{-1})\\&\lesssim |\omega |^{-2+3\varepsilon }(1\vee \log (\delta ^{-1})). \end{aligned} \end{aligned}$$

#### Proof

To bound (C.4) it suffices to estimate the two parts

C.5 $$\begin{aligned} \sum _{\begin{array}{c} m_1\in \mathbb {Z}^{2}\\ |m_1|\le \delta ^{-1} \end{array}}\sum _{\omega _1\in \mathbb {Z}^{2}\setminus \{0,\omega \}}(1+|\omega _1-m_1|^{2})^{-1}(1+|\omega -\omega _1+m_1|^{2})^{-1}|\omega _1|^{-2} \end{aligned}$$

and

C.6 $$\begin{aligned} \sum _{\begin{array}{c} m_1\in \mathbb {Z}^{2}\\ |m_1|\le \delta ^{-1} \end{array}}\sum _{\omega _1\in \mathbb {Z}^{2}\setminus \{0,\omega \}}(1+|\omega _1-m_1|^{2})^{-1}(1+|\omega -\omega _1+m_1|^{2})^{-1}|\omega _1|^{-2}|\omega -\omega _1|^{-1}.\nonumber \\ \end{aligned}$$

Let us consider (C.5). We decompose the sum over $m_1$ into the regions $m_1=0$, $m_1=-\omega$ and $m_1\in \mathbb {Z}^{2}\setminus \{0,-\omega \}$.

The sum over $m_1\in \mathbb {Z}^{2}\setminus \{0,-\omega \}$ is given by

$$\begin{aligned} \sum _{\begin{array}{c} m_1\in \mathbb {Z}^{2}\setminus \{0,-\omega \}\\ |m_1|\le \delta ^{-1} \end{array}}\sum _{\omega _1\in \mathbb {Z}^{2}\setminus \{0,\omega \}}(1+|\omega _1-m_1|^{2})^{-1}(1+|\omega -\omega _1+m_1|^{2})^{-1}|\omega _1|^{-2}. \end{aligned}$$

The sum over $\omega _1\in \mathbb {Z}^{2}\setminus \{0,\omega \}$ can then be further decomposed into the regions $\omega _1=m_1$, $\omega _1=\omega +m_1$ and $\omega _1\in \mathbb {Z}^{2}\setminus \{0,\omega ,m_1,\omega +m_1\}$.

We only give the bound that involves the sums over $m_1\in \mathbb {Z}^{2}\setminus \{0,-\omega \}$ and $\omega _1\in \mathbb {Z}^{2}\setminus \{0,\omega ,m_1,\omega +m_1\}$. Using that $\omega _1\in \mathbb {Z}^{2}\setminus \{0,\omega ,m_1,\omega +m_1\}$, we may estimate

$$\begin{aligned} \begin{aligned}&\sum _{\begin{array}{c} m_1\in \mathbb {Z}^{2}\setminus \{0,-\omega \}\\ |m_1|\le \delta ^{-1} \end{array}}\sum _{\omega _1\in \mathbb {Z}^{2}\setminus \{0,\omega ,m_1,\omega +m_1\}}(1+|\omega _1-m_1|^{2})^{-1}(1+|\omega -\omega _1+m_1|^{2})^{-1}|\omega _1|^{-2}\\&\le \sum _{\begin{array}{c} m_1\in \mathbb {Z}^{2}\setminus \{0,-\omega \}\\ |m_1|\le \delta ^{-1} \end{array}}\sum _{\omega _1\in \mathbb {Z}^{2}\setminus \{0,\omega ,m_1,\omega +m_1\}}|\omega _1-m_1|^{-2}|\omega -\omega _1+m_1|^{-2}|\omega _1|^{-2}. \end{aligned} \end{aligned}$$

Introducing the dyadic partition of unity $(\varrho _{q})_{q\in \mathbb {N}_{-1}}$, we decompose this sum into

C.7 $$\begin{aligned}&\sum _{\begin{array}{c} m_1\in \mathbb {Z}^{2}\setminus \{0,-\omega \}\\ |m_1|\le \delta ^{-1} \end{array}}\sum _{\omega _1\in \mathbb {Z}^{2}\setminus \{0,\omega ,m_1,\omega +m_1\}}|\omega _1-m_1|^{-2}|\omega -\omega _1+m_1|^{-2}|\omega _1|^{-2}\nonumber \\&=\sum _{\begin{array}{c} m_1\in \mathbb {Z}^{2}\setminus \{0,-\omega \}\\ |m_1|\le \delta ^{-1} \end{array}}\sum _{\begin{array}{c} \omega _1\in \mathbb {Z}^{2}\setminus \{0,\omega ,m_1,\omega +m_1\}\\ \omega _1\precsim (\omega -\omega _1+m_1) \end{array}}|\omega _1-m_1|^{-2}|\omega -\omega _1+m_1|^{-2}|\omega _1|^{-2} \end{aligned}$$

C.8 $$\begin{aligned}&\quad +\sum _{\begin{array}{c} m_1\in \mathbb {Z}^{2}\setminus \{0,-\omega \}\\ |m_1|\le \delta ^{-1} \end{array}}\sum _{\begin{array}{c} \omega _1\in \mathbb {Z}^{2}\setminus \{0,\omega ,m_1,\omega +m_1\}\\ \omega _1\succsim (\omega -\omega _1+m_1) \end{array}}|\omega _1-m_1|^{-2}|\omega -\omega _1+m_1|^{-2}|\omega _1|^{-2}\end{aligned}$$

C.9 $$\begin{aligned}&\quad +\sum _{\begin{array}{c} m_1\in \mathbb {Z}^{2}\setminus \{0,-\omega \}\\ |m_1|\le \delta ^{-1} \end{array}}\sum _{\begin{array}{c} \omega _1\in \mathbb {Z}^{2}\setminus \{0,\omega ,m_1,\omega +m_1\}\\ \omega _1\sim (\omega -\omega _1+m_1) \end{array}}|\omega _1-m_1|^{-2}|\omega -\omega _1+m_1|^{-2}|\omega _1|^{-2}. \end{aligned}$$

Assume $\varepsilon <2/3$. The terms (C.7) and (C.9) can be estimated by two applications of Lemma C.3,

$$\begin{aligned} \begin{aligned}&\sum _{\begin{array}{c} m_1\in \mathbb {Z}^{2}\setminus \{0,-\omega \}\\ |m_1|\le \delta ^{-1} \end{array}}\sum _{\begin{array}{c} \omega _1\in \mathbb {Z}^{2}\setminus \{0,\omega ,m_1,\omega +m_1\}\\ |\omega _1|\le 64/9|\omega -\omega _1+m_1| \end{array}}|\omega _1-m_1|^{-2}|\omega -\omega _1+m_1|^{-2}|\omega _1|^{-2}\\&\lesssim \sum _{\begin{array}{c} m_1\in \mathbb {Z}^{2}\setminus \{0,-\omega \}\\ |m_1|\le \delta ^{-1} \end{array}}|\omega +m_1|^{-2}\sum _{\begin{array}{c} \omega _1\in \mathbb {Z}^{2}\setminus \{0,\omega ,m_1,\omega +m_1\}\\ |\omega _1|\le 64/9|\omega -\omega _1+m_1| \end{array}}|\omega _1-m_1|^{-2}|\omega _1|^{-2}\\&\lesssim \sum _{\begin{array}{c} m_1\in \mathbb {Z}^{2}\setminus \{0,-\omega \}\\ |m_1|\le \delta ^{-1} \end{array}}|\omega +m_1|^{-2}|m_1|^{-2+2\varepsilon }\lesssim |\omega |^{-2+3\varepsilon }. \end{aligned} \end{aligned}$$

The second term (C.8) can be estimated by Lemma C.3 and (C.2),

$$\begin{aligned} \begin{aligned}&\sum _{\begin{array}{c} m_1\in \mathbb {Z}^{2}\setminus \{0,-\omega \}\\ |m_1|\le \delta ^{-1} \end{array}}\sum _{\begin{array}{c} \omega _1\in \mathbb {Z}^{2}\setminus \{0,\omega ,m_1,\omega +m_1\}\\ \omega _1\succsim (\omega -\omega _1+m_1) \end{array}}|\omega _1-m_1|^{-2}|\omega -\omega _1+m_1|^{-2}|\omega _1|^{-2}\\&\lesssim \sum _{\begin{array}{c} m_1\in \mathbb {Z}^{2}\setminus \{0,-\omega \}\\ |m_1|\le \delta ^{-1} \end{array}}|\omega +m_1|^{-2}\sum _{\begin{array}{c} \omega _1\in \mathbb {Z}^{2}\setminus \{0,\omega ,m_1,\omega +m_1\}\\ \omega _1\succsim (\omega -\omega _1+m_1) \end{array}}|\omega _1-m_1|^{-2}|\omega -\omega _1+m_1|^{-2}\\&\lesssim |\omega |^{-2+2\varepsilon }\sum _{\begin{array}{c} m_1\in \mathbb {Z}^{2}\setminus \{0,-\omega \}\\ |m_1|\le \delta ^{-1} \end{array}}|\omega +m_1|^{-2}\lesssim |\omega |^{-2+3\varepsilon }(1\vee \log (\delta ^{-1})). \end{aligned} \end{aligned}$$

Decomposing (C.5) as discussed and bounding the resulting terms yields

$$\begin{aligned}  &   \sum _{\begin{array}{c} m_1\in \mathbb {Z}^{2}\\ |m_1|\le \delta ^{-1} \end{array}}\sum _{\omega _1\in \mathbb {Z}^{2}\setminus \{0,\omega \}}(1+|\omega _1-m_1|^{2})^{-1}(1+|\omega -\omega _1+m_1|^{2})^{-1}|\omega _1|^{-2}\\  &   \quad \lesssim |\omega |^{-2+3\varepsilon }(1\vee \log (\delta ^{-1})). \end{aligned}$$

Assume $\varepsilon \in (0,1/2)$. The bound on (C.6) then follows in a similar manner. $\square$

## D Glossary

In this glossary we collect our frequently-used symbols.

**Table:**

Table of distribution spaces, noise objects and Feynman diagrams		
Space	Description	Reference
$C^{\infty }$	The smooth, periodic functions on $\mathbb {T}^{2}$.	Section 1.1
$\mathcal {S}'$	The periodic distributions on $\mathbb {T}^{2}$.	Section 1.1
$\mathcal {B}_{p,q}^{\alpha }$	The completion of $C^{\infty }$ under the Besov-norm $\Vert \,\cdot \,\Vert _{\mathcal {B}^{\alpha }_{p,q}}$.	Definition A.1
$\mathcal {C}^{\alpha }$	The Hölder–Besov space $\mathcal {C}^{\alpha }=\mathcal {B}_{\infty ,\infty }^{\alpha }$.	Definition A.1
$\mathcal {H}^{2}$	The Sobolev space $\mathcal {H}^{2}=\mathcal {B}_{2,2}^{2}$.	Section 1.1
$C_{T}E$	The continuous functions $f:[0,T]\rightarrow E$.	Section 1.1
$C_{T}^{\kappa }E$	The $\kappa$-Hölder continuous functions $f:[0,T]\rightarrow E$.	Section 1.1
$C_{\eta ;T}E$	The continuous functions $f:(0,T]\rightarrow E$ with blow-up of at most $t^{-\eta }$.	Section 1.1
$C_{\eta ;T}^{\kappa }E$	The $\kappa$-Hölder functions $f:(0,T]\rightarrow E$ with blow-up of at most $t^{-\eta }$.	Section 1.1
$\mathscr {L}_{\eta ;T}^{\kappa }\mathcal {C}^{\alpha }$	The weighted interpolation space $\mathscr {L}_{\eta ;T}^{\kappa }\mathcal {C}^{\alpha }=C_{\eta ;T}^{\kappa }\mathcal {C}^{\alpha -2\kappa }\cap C_{\eta ;T}\mathcal {C}^{\alpha }$.	Definition A.3
$\mathcal {X}^{\alpha ,\kappa }_T$	The space of enhanced rough noises.	Definition 2.2
$\mathscr {D}_T$	The space of paracontrolled distributions.	Definition 3.4
	The normed vector space *F* equipped with a cemetery state .	Definition 3.16
$F^{\text {sol}}_{\eta ;T}$	The continuous that do not resurrect, with weight at 0.	Definition 3.19
$\varvec{\xi }$	The vector-valued space-time white-noise $\varvec{\xi }=(\xi ^1,\xi ^2)$	(2.1)
	$$=\nabla \cdot \mathcal {I}[\sigma \varvec{\xi }]$$	(2.2)
		Section 2.2
		Section 2.2
		Section 2.2
		Section 2.2
		Section 2.2
		Section 2.2
	$$=\nabla \cdot \mathcal {I}[\sigma (\psi _{\delta }*\varvec{\xi })]$$	(2.4)
		Section 2.2
		Section 2.2
	(2.7) applied to	Section 2.4
	(2.7) applied to	Section 2.5

## Footnotes

1 See [54] for a related model.

2 Here $\mathcal {C}^{\alpha }_{\text {par}}$ denotes the set of space-time Hölder-regular distributions of parameter $\alpha \in \mathbb {R}$ equipped with the parabolic scaling, i.e. regularity in time counts twice as much as regularity in space. These spaces are not used beyond the introduction and so we refer to [26, Lem. 2.12 & Def. 3.7] for an example definition. The shorthand ${\alpha \pm }$ is used to denote $\alpha \pm \varepsilon$ for any $\varepsilon >0$ but fixed.

3 The remainder $(\nabla \Phi _w)^{\#}$ can be expressed in terms of $\nabla \Phi _{w^{\#}}$ and a more regular commutator, cf. Lemma A.9. Hence, the paracontrolled Ansatz for *w* implies the Ansatz for $\nabla \Phi _{w}$. In fact in Sect. 3 we make use of an equivalent Ansatz which makes certain technical steps easier but is less clear to present—see Remark 3.5.

## Code

N/A
